# Oral mucositis risk with EGFR tyrosine kinase inhibitor monotherapies and combination regimens in non-small cell lung cancer: a systematic review and network meta-analysis

**DOI:** 10.3389/fimmu.2026.1957825

**Published:** 2026-09-18

**Authors:** Huiwen Sun, Yifan Yao, Xinyu Liu, Ruiyi Qiu, Jinhan Chen, Gaochenxi Zhang, Qijin Shu

**Affiliations:** 1 Department of Oncology, The First Affiliated Hospital of Zhejiang Chinese Medical University (Zhejiang Provincial Hospital of Chinese Medicine), Hangzhou, Zhejiang, China; 2 The First School of Clinical Medicine, Zhejiang Chinese Medical University, Hangzhou, Zhejiang, China

**Keywords:** adverse events, combination therapy, epidermal growth factor receptor tyrosine kinase inhibitors (EGFR TKIs), network meta-analysis, non-small cell lung cancer, oral mucositis

## Abstract

**Introduction and aims:**

Oral mucositis (OM) impairs quality of life, necessitates dose modification, and disrupts treatment continuity in patients receiving EGFR tyrosine kinase inhibitors (EGFR-TKIs), yet comparative OM risks across monotherapies and combination regimens remain poorly defined.

**Methods:**

PubMed, Embase, Web of Science, the Cochrane Library, and ClinicalTrials.gov were searched from inception to June 13, 2026, for phase II or III randomized controlled trials. Bayesian random-effects network meta-analyses estimated odds ratios (ORs) with 95% credible intervals (CrIs) for any-grade and grade ≥3 OM. Treatments were ranked using the surface under the cumulative ranking curve (SUCRA).

**Results:**

Sixty-three trials involving 21,845 participants were included. Compared with chemotherapy, higher odds of any-grade OM were observed with afatinib plus cetuximab (OR 11.97, 95% CrI 2.27–65.45), afatinib (OR 9.73, 95% CrI 3.75–26.50), dacomitinib (OR 5.12, 95% CrI 1.71–16.00), and erlotinib plus chemotherapy (OR 2.84, 95% CrI 1.27–6.53), with low-, high-, moderate-, and very-low-certainty evidence, respectively; for grade ≥3 OM, with afatinib plus cetuximab (OR 13.91, 95% CrI 1.55–199.26), afatinib (OR 8.80, 95% CrI 1.37–100.11), and mobocertinib (OR 8.12, 95% CrI 1.14–97.77), all supported by moderate-certainty evidence. Afatinib plus cetuximab ranked highest for both outcomes (SUCRA, 89.0% and 91.6%). Second-generation EGFR-TKIs generally ranked higher, whereas the third-generation agents osimertinib and furmonertinib ranked lower. Combination regimens often yielded higher point estimates than matched monotherapies, but most comparisons were inconclusive.

**Conclusions:**

OM risk varies substantially across EGFR-TKI regimens. Higher-risk regimens warrant strengthened baseline oral assessment, patient education, and monitoring during treatment. Given limited head-to-head evidence for novel agents/combinations and imprecision in some estimates, findings warrant further validation in standardized prospective studies.

**Systematic Review Registration:**

https://www.crd.york.ac.uk/PROSPERO/view/CRD420261449930, identifier CRD420261449930.

## 1 Introduction

Lung cancer remains the leading cause of cancer-related mortality worldwide, with non-small cell lung cancer (NSCLC) accounting for approximately 80–85% of cases (1, 2). Activating EGFR mutations occur in approximately 10–20% of White patients and up to 50% of Asian patients, making EGFR a key therapeutic target (3, 4). The successive development of three generations of EGFR-TKIs and EGFR-TKI-based combination regimens has substantially expanded treatment options for advanced NSCLC (5, 6). However, treatment-related toxicities remain a major challenge in clinical management. Common adverse events, such as rash, paronychia, and diarrhea, are well characterized, whereas rare but potentially fatal toxicities, including interstitial lung disease, severe hepatotoxicity, and cardiovascular complications, have also attracted substantial clinical and research attention (7, 8). By contrast, oral mucositis (OM), a common adverse event of EGFR-TKI therapy, has received limited attention, leaving its clinical burden potentially underestimated.

Oral mucositis (OM) is a clinically important oral toxicity of anticancer therapy. With the widespread use of molecularly targeted therapies, including EGFR-TKIs, the traditional concept of chemoradiotherapy-induced OM has expanded to include targeted therapy-associated oral mucosal injury (9, 10). Mild cases may present with mucosal sensitivity, burning, or localized erythema, and shallow ulcerative or aphthous-like lesions, whereas severe cases can progress to extensive erosions, ulceration, and pseudomembrane formation, causing intense pain and impaired eating, swallowing, or speech. These complications may compromise nutritional status and quality of life and lead to dose reduction, treatment delay, or interruption (11–14). Published phase III trials of EGFR-TKIs have reported incidences of up to 64.4% for any-grade OM and 4.4% for grade ≥3 OM in the experimental groups (15). Pharmacovigilance analyses, real-world retrospective studies, and case reports have further corroborated the clinical relevance of EGFR-TKI-associated oral mucosal inflammation, ulcerative lesions, and painful symptoms (13, 16–21). Accordingly, characterizing the incidence, comparative risk, and clinical consequences of EGFR-TKI-associated OM has direct clinical relevance.

However, current evidence on OM remains scattered across individual clinical trials, in which it is typically reported as a secondary safety outcome. Previous meta-analyses have largely focused on the overall toxicity profiles of EGFR-TKIs, with insufficient systematic investigation of oral mucosal toxicities (22, 23). Furthermore, direct head-to-head evidence comparing oral mucositis (OM) risk across individual EGFR-TKI monotherapies and combination regimens remains limited, leaving their relative toxicity profiles and rankings uncertain. We therefore conducted a systematic review and Bayesian network meta-analysis of randomized controlled trials to synthesize direct and indirect evidence, compare any-grade and grade ≥3 OM risks across EGFR-TKI monotherapy and combination regimens in advanced NSCLC, and quantitatively rank regimens by oral mucosal toxicity. This work aims to provide a reference for individualized treatment selection and risk-stratified oral toxicity monitoring.

## 2 Methods

This systematic review and Bayesian network meta-analysis was conducted in accordance with PRISMA-NMA and registered in PROSPERO (CRD420261449930) (24).

### 2.1 Eligibility criteria

Eligibility criteria were established according to the PICOS framework (Population, Intervention, Comparator, Outcomes, and Study design):

1. Population: Patients with pathologically or cytologically confirmed advanced NSCLC (stage III–IV or recurrent disease).
2. Interventions: Eligible experimental interventions included EGFR-TKI monotherapy—gefitinib, erlotinib, afatinib, dacomitinib, osimertinib, furmonertinib, aumolertinib, lazertinib, or mobocertinib—including an EGFR-TKI administered with a matched placebo, as well as EGFR-TKI–based combinations with chemotherapy, antiangiogenic agents, EGFR-directed antibodies, or other targeted agents.
3. Comparators: Eligible comparators included placebo, chemotherapy with or without placebo, EGFR-TKI monotherapy, or another eligible EGFR-TKI–based regimen. For network construction, because the primary aim was to compare OM risk across EGFR-TKI–based regimens rather than individual chemotherapy backbones, all chemotherapy regimens were grouped into a single chemotherapy node. Given the limited direct evidence across TKI regimens, separating chemotherapy backbones would have fragmented the network and reduced the precision of indirect comparisons.
4. Outcomes: The primary outcome was any-grade oral mucositis (OM), and the secondary outcome was grade ≥3 OM.
5. Study design: Phase II or III randomized controlled trials (RCTs).

Studies not meeting these criteria were excluded, including observational studies, single-arm studies, case reports, reviews, and dose-escalation trials. Trials enrolling fewer than 30 randomized participants in total were excluded because adverse-event estimates from very small trials were considered insufficiently precise and potentially unstable. Studies without reliably extractable OM event counts and corresponding denominators were also excluded. When multiple publications reported results from the same trial, complementary information was collated across reports, and the most complete and up-to-date report was used as the primary data source.

### 2.2 Data sources and extraction

A systematic search was conducted in PubMed, Embase, the Cochrane Library, Web of Science, and ClinicalTrials.gov from database inception to June 13, 2026. Relevant data were retrieved from full-text articles, **Supplementary Materials**, and all publicly available registry records; full search strategies for each database are detailed in **Supplementary Table S1**. Extracted data items included basic study characteristics, baseline patient profiles, intervention regimen details, outcome metrics, and AE grading system and version. These extracted characteristics served as potential effect modifiers for assessing transitivity across treatment comparisons. OM-related outcomes were identified according to the terminology used in the original reports. Events reported as “oral mucositis” or “stomatitis” were harmonized as OM, as these terms are widely interchangeable in oncology clinical reporting and have historically been grouped or directly mapped within the NCI toxicity classification system for oral mucosal toxicity. Literature screening and data extraction were independently performed by two investigators (Liu XY, Yao YF), with any discrepancies adjudicated by a senior researcher (Sun HW) via consensus discussion.

### 2.3 Risk of bias assessment

The revised Cochrane Risk of Bias tool for randomized trials (RoB 2) was used to assess the oral mucositis outcome (24). Two reviewers independently evaluated five domains: randomization, deviations from intended interventions, missing outcome data, outcome measurement, and selection of the reported result. Each outcome was classified as low risk, some concerns, or high risk. Disagreements were resolved by consensus or, when necessary, by a third reviewer. Summary plots were generated using the RoB 2 Excel tool.

### 2.4 Statistical analysis

Bayesian network meta-analyses were conducted in R using the gemtc package with JAGS (25). Arm-level event counts for any-grade and grade ≥3 oral mucositis (OM) were modeled using a binomial likelihood with a logit link. Relative treatment effects were reported as odds ratios (ORs) with 95% credible intervals (CrIs), with CrIs excluding 1 indicating evidence of a treatment difference. Random-effects models were used for the primary analyses, with fixed-effect models assessed using the deviance information criterion (DIC) and residual deviance.

For any-grade OM, four chains were run with 20,000 adaptation iterations followed by 50,000 sampling iterations without thinning. For grade ≥3 OM, four chains were run with 50,000 adaptation iterations followed by 200,000 sampling iterations, with a thinning interval of 10 (26, 27). Convergence was assessed using trace and density plots and the Brooks–Gelman–Rubin diagnostic. Between-study heterogeneity was summarized using the posterior distribution of the between-study standard deviation, τ. Global inconsistency was assessed by comparing consistency and unrelated mean-effects models, and local inconsistency was evaluated using node-splitting analyses.

For grade ≥3 OM, double-zero trials were excluded because they provide no information on relative ORs. Given the rarity of the outcome and potential data separation, weakly informative normal priors were assigned to the basic treatment effects on the log-OR scale $\left[N(0,2.82^{2})\right]$ (28–31), and a half-normal prior with a scale parameter of 0.5 was assigned to τ (28, 29, 32, 33). Consequently, the rare-event analysis was informed only by trials with at least one event, while the weakly informative priors helped stabilize sparse-data estimates and reduce instability from separation.

Treatment rankings were summarized using the surface under the cumulative ranking curve (SUCRA), with higher values indicating a greater risk of OM. Exploratory subgroup analyses for any-grade OM were conducted according to treatment line and population composition. Sensitivity analyses used alternative heterogeneity priors for any-grade OM and alternative heterogeneity and treatment-effect priors and a fixed-effect model for grade ≥3 OM.

### 2.5 Assessment of certainty of evidence

The certainty of evidence for each network estimate was assessed using the CINeMA framework across six domains: within-study bias, reporting bias, indirectness, imprecision, heterogeneity, and incoherence (34). Certainty was rated as high, moderate, low, or very low. Disagreements were resolved by consensus or, if necessary, by a third reviewer.

## 3 Results

### 3.1 Study selection and characteristics

The search identified 8,615 records. After duplicate removal and screening, 471 reports underwent full-text assessment, and 63 randomized controlled trials (RCTs) involving 21,845 randomized participants were included in the systematic review and network meta-analysis. The study-selection process is shown in **Figure 1**.

**Figure 1:**
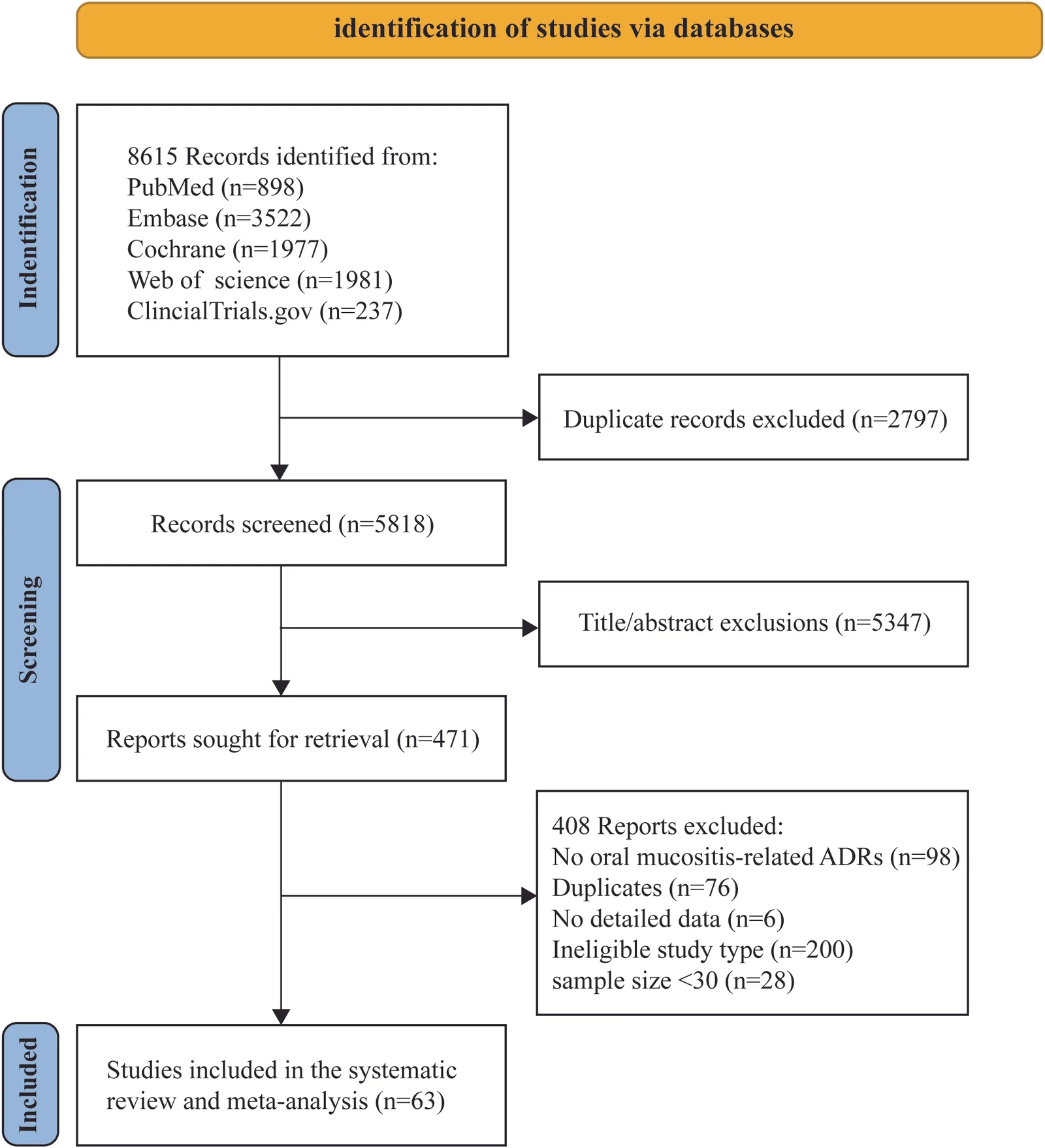
PRISMA flow diagram of literature search and study inclusion process.

The trials were published between 2005 and 2025 and included 25 phase II trials, 37 phase III trials, and one phase II/III trial; one used a three-arm design. Thirty-six trials evaluated first-line treatment, 26 evaluated second-line or later treatment, and one did not specify the treatment line. Twenty-six trials enrolled exclusively Asian populations, and most involved patients with advanced, metastatic, or recurrent NSCLC. The network comprised 24 treatment nodes, including nine EGFR-TKI monotherapies, 13 EGFR-TKI–based combinations, chemotherapy, and placebo. Any-grade and grade ≥3 oral mucositis were reported in 61 and 50 trials, respectively. Detailed characteristics are provided in **Supplementary Table S2**.

While baseline characteristics were not fully homogeneous across included trials, all were phase II/III randomized trials of patients with advanced or recurrent NSCLC, with treatment-related OM assessed under broadly comparable systemic treatment settings. Transitivity was therefore considered clinically plausible, though residual differences in potential effect modifiers could not be ruled out.

### 3.2 Risk of bias and certainty of evidence

Among the 63 included RCTs, 28 (44.4%) were judged to be at low overall risk of bias and 35 (55.6%) raised some concerns. Concerns mainly involved outcome measurement and the randomization process. Detailed assessments are presented in **Supplementary Figure S1**. The certainty of evidence ranged from high to very low across network comparisons according to the CINeMA framework. Certainty ratings for key comparisons are reported alongside the corresponding effect estimates in Sections 3.3 and 3.4, with detailed assessments for any-grade and grade ≥3 OM provided in **Supplementary Figure S2**.

### 3.3 Any-grade oral mucositis

The network for any-grade OM included 61 RCTs involving 20,881 participants and 24 treatment nodes (**Figure 2A**). Compared with chemotherapy, afatinib plus cetuximab (OR 11.97, 95% CrI 2.27–65.45), afatinib (OR 9.73, 95% CrI 3.75–26.50), dacomitinib (OR 5.12, 95% CrI 1.71–16.00), and erlotinib plus chemotherapy (OR 2.84, 95% CrI 1.27–6.53) were associated with higher odds of any-grade OM; these comparisons were supported by low-, high-, moderate-, and very-low-certainty evidence, respectively. In contrast, placebo was associated with lower odds (OR 0.25, 95% CrI 0.07–0.94; low-certainty evidence) (**Figure 3A**; **Supplementary Figure S2**).

**Figure 2:**
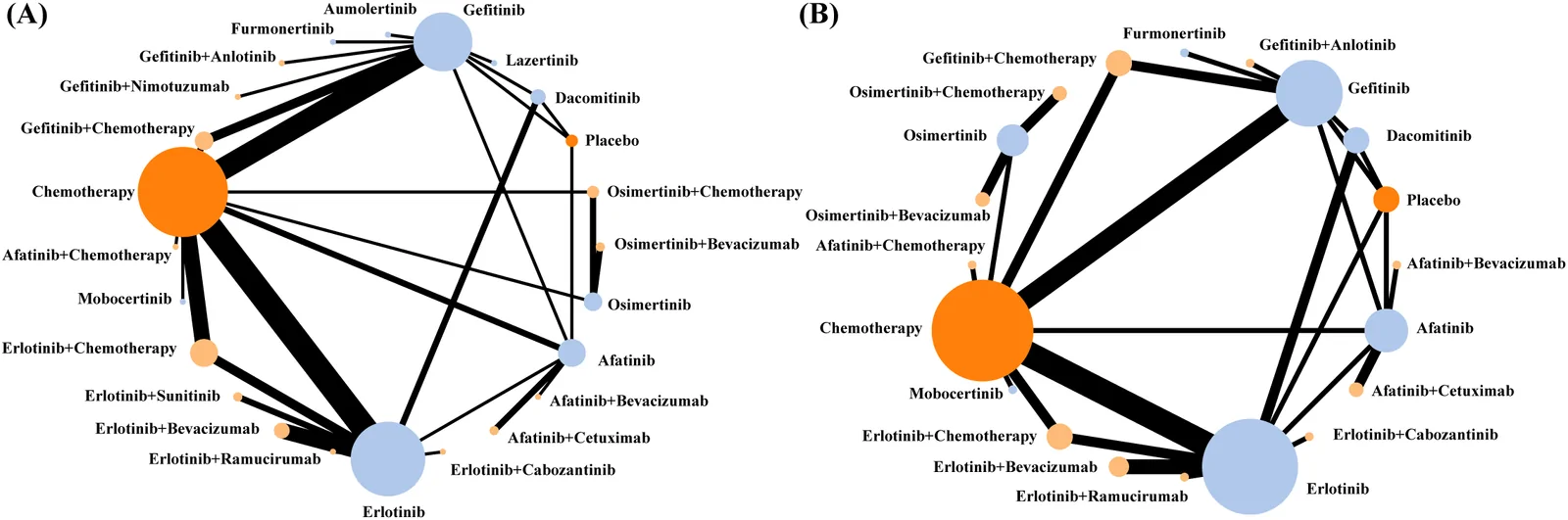
Network diagrams of treatment comparisons for any-grade **(A)** and grade ≥3 **(B)** oral mucositis. Each node represents a treatment regimen, with node size proportional to the number of participants assigned to that regimen. Lines indicate direct comparisons, and line width is proportional to the number of trials informing each comparison. Blue nodes represent EGFR-TKI monotherapies, orange nodes represent placebo or chemotherapy, and light-orange nodes represent EGFR-TKI–based combination regimens.

**Figure 3:**
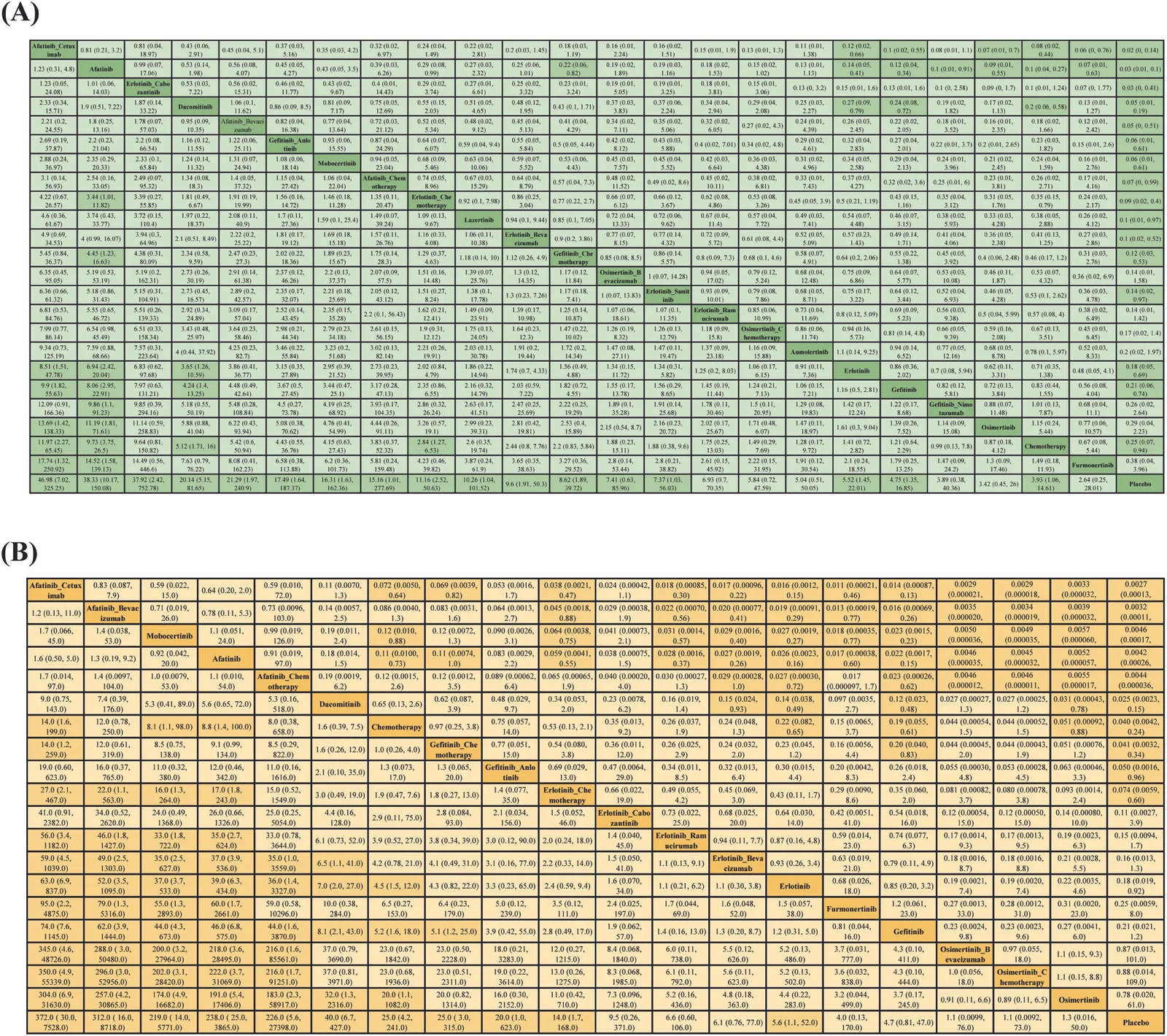
League tables of comparative risks of any-grade **(A)** and grade ≥3 **(B)** oral mucositis across EGFR-TKI monotherapies and combination regimens. Results are presented as odds ratios (ORs) with 95% credible intervals (CrIs). Odds ratios greater than 1 indicate a higher OM risk for the treatment listed in the row than for the treatment listed in the column.

Among EGFR-TKI monotherapies, second-generation agents ranked highest for OM risk and first-generation agents occupied intermediate positions; greater variation was observed among third-generation and other newer agents, with osimertinib and furmonertinib ranking lowest. Afatinib was associated with higher odds of OM than erlotinib, gefitinib, osimertinib, and furmonertinib. Dacomitinib was associated with higher odds than erlotinib and gefitinib. For combination regimens, the point estimates were higher than those of the corresponding monotherapies for all erlotinib-based combinations, gefitinib plus anlotinib or chemotherapy, and osimertinib plus bevacizumab or chemotherapy. However, all 95% CrIs for combination-versus-monotherapy comparisons included 1, and no clear differences were observed among the combination regimens (**Figure 3A**).

Based on SUCRA values, afatinib plus cetuximab (SUCRA, 89.0%), afatinib (SUCRA, 88.4%), and erlotinib plus cabozantinib (SUCRA, 81.2%) ranked highest for any-grade OM risk. Furmonertinib (SUCRA, 21.0%) and placebo (SUCRA, 3.5%) ranked second lowest and lowest overall, respectively (**Figures 4A, B**, **Supplementary Table S3**).

**Figure 4:**
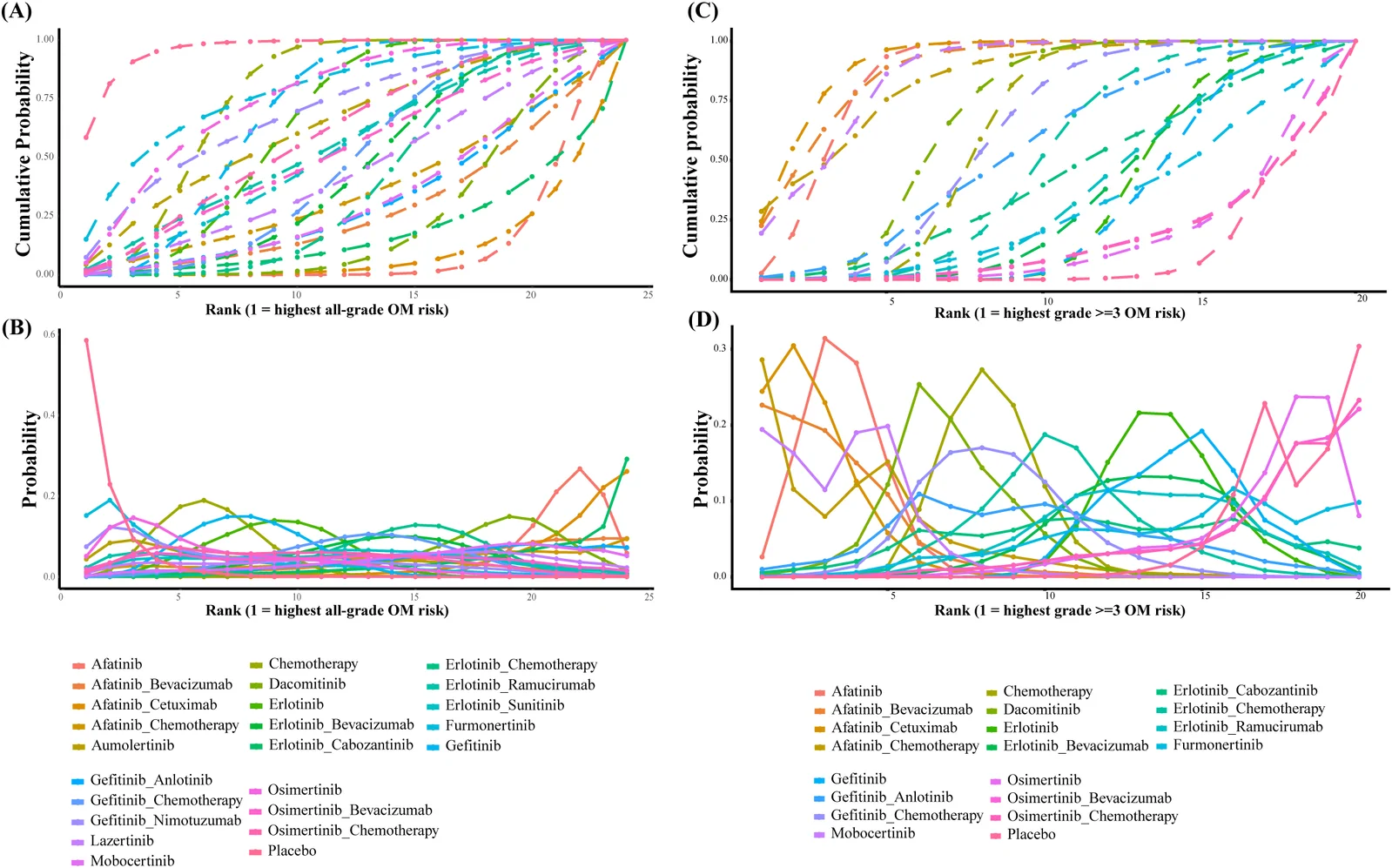
Ranking probabilities of EGFR-TKI–based regimens for any-grade and grade ≥3 oral mucositis. **(A, B)** Cumulative ranking curves and rank probability plots for any-grade oral mucositis; **(C, D)** cumulative ranking curves and rank probability plots for grade ≥3 oral mucositis. Rank 1 represents the highest estimated OM risk.

### 3.4 Grade ≥3 oral mucositis

Grade ≥3 OM was reported in 50 RCTs. After the exclusion of eight double-zero trials, 42 RCTs involving 15,903 participants and 20 treatment nodes were included in the network meta-analysis (**Figure 2B**). Compared with chemotherapy, afatinib plus cetuximab (OR 13.91, 95% CrI 1.55–199.26), afatinib (OR 8.80, 95% CrI 1.37–100.11), and mobocertinib (OR 8.12, 95% CrI 1.14–97.77) were associated with higher odds of grade ≥3 OM; all three comparisons were supported by moderate-certainty evidence according to CINeMA. In contrast, erlotinib (OR 0.22, 95% CrI 0.08–0.65; very-low certainty), gefitinib (OR 0.19, 95% CrI 0.05–0.61; very-low certainty), osimertinib (OR 0.05, 95% CrI 0.001–0.88; very-low certainty), and placebo (OR 0.04, 95% CrI 0.004–0.24; moderate certainty) were associated with lower odds (**Figure 3B**; **Supplementary Figure S2**).

Among EGFR-TKI monotherapies, both afatinib and mobocertinib were associated with higher odds of grade ≥3 OM than erlotinib, gefitinib, osimertinib, and furmonertinib, whereas dacomitinib was associated with higher odds than erlotinib, gefitinib, and osimertinib. Among matched combination-versus-monotherapy comparisons, only gefitinib plus chemotherapy was associated with higher odds than gefitinib alone; no clear differences were observed for the remaining combinations (**Figure 3B**).

Afatinib plus cetuximab ranked highest for grade ≥3 OM risk according to SUCRA (91.6%), followed by afatinib plus bevacizumab (88.4%), mobocertinib (86.4%), and afatinib (86.3%). Placebo ranked lowest (10.2%), while osimertinib and its combination regimens also ranked near the lower end (16.6%–16.9%). Given the rarity of severe events and the imprecision of several estimates, particularly those involving combinations, the high-ranking results should be interpreted cautiously (**Figures 4C, D**, **Supplementary Table S3**).

### 3.5 Model diagnostics and sensitivity analyses

For any-grade OM, the random-effects model provided a substantially better fit than the fixed-effect model (DIC 239.9 vs 386.1). For grade ≥3 OM, the two models showed comparable fit (DIC 139.7 vs 141.3). The posterior median between-study standard deviations for the two outcomes were 0.89 (95% CrI 0.65–1.24) and 0.36 (95% CrI 0.02–0.95), respectively. Comparisons between the consistency and unrelated mean-effects models, together with node-splitting analyses, indicated no important inconsistency. Both primary models showed satisfactory convergence, with Brooks–Gelman–Rubin potential scale reduction factors close to 1.00. Outcome-specific sensitivity analyses using alternative prior distributions and model specifications yielded broadly consistent findings, supporting the robustness of the primary results (**Supplementary Figures S3**, **S4**, **Supplementary Tables S4**-**S8**).

### 3.6 Exploratory subgroup analysis

Exploratory subgroup analyses showed that, among treatments shared with the primary network, the overall risk rankings were broadly consistent with the primary analysis in the first-line, exclusively Asian, and non-exclusively Asian subgroups. The second-line or later subgroup (≥2L) also retained some of the principal high-risk signals, although treatment rankings varied more substantially. Heterogeneity was lower in the first-line and exclusively Asian subgroups but higher in the ≥2L and non-exclusively Asian subgroups (first-line, τ=0.16; ≥2L, τ=1.41; exclusively Asian, τ=0.43; non-exclusively Asian, τ=1.12), suggesting that treatment line and study-population composition may be associated with the observed heterogeneity (**Supplementary Figures S5**, **S6**, **Supplementary Tables S9**, **S10**). Given the limited size of several subgroup networks, observed ranking differences may reflect imprecision rather than true effect modification and should therefore be interpreted cautiously as exploratory findings.

## 4 Discussion

This Bayesian network meta-analysis identified substantial differences in OM risk across EGFR-TKI regimens. The two second-generation TKIs, afatinib and dacomitinib, ranked toward the higher-risk end for both any-grade and grade ≥3 OM, whereas the newer agent mobocertinib showed a higher estimated risk signal for grade ≥3 OM. By contrast, the third-generation TKIs osimertinib and furmonertinib showed relatively low risks of any-grade OM, and osimertinib also ranked low for grade ≥3 OM. Several combinations yielded higher point estimates than matched monotherapies. Afatinib plus cetuximab showed the highest estimated risk. Meanwhile, risk estimates and SUCRA rankings generally shifted upward for first-generation EGFR-TKIs combined with chemotherapy, antiangiogenic agents, or multitargeted inhibitors, and for osimertinib combined with bevacizumab or chemotherapy.

The ErbB receptor family (EGFR, HER2, HER3, HER4) forms an integrated signaling network via homo- and heterodimerization, coordinately regulating epithelial proliferation, migration and survival (35–39). EGF–EGFR signaling drives oral epithelial proliferation, migration and post-injury re-epithelialization (37, 38, 40); HER4 sustains epithelial survival under inflammatory stress via AKT-mediated anti-apoptotic signaling (41), while HER2, the preferred dimerization partner of EGFR and HER4, amplifies these signals (42). EGFR blockade disrupts epithelial turnover and mucosal barrier repair, increases keratinocyte apoptosis (43), and dysregulates local chemokine expression and inflammatory responses (44). Compromised barrier integrity permits microbial colonization, which further amplifies tissue injury and drives mucosal erosion and ulceration (45); concurrent HER2/HER4 inhibition additionally impairs residual and compensatory epithelial repair.

Second-generation irreversible pan-ErbB inhibitors afatinib and dacomitinib target both wild-type and mutant EGFR while durably blocking HER2/HER4 signaling; this broad (46, 47), sustained suppression of epithelial proliferative, migratory and survival pathways may partly explain the higher OM risk observed with these agents. First-generation reversible EGFR-TKIs gefitinib and erlotinib primarily target EGFR, with residual wild-type activity but no pan-ErbB blockade (2, 7), conferring lower OM risk than second-generation agents. Third-generation EGFR-TKIs osimertinib and furmonertinib are highly selective for sensitizing and T790M resistance mutations, with relative sparing of wild-type EGFR signaling (48), minimizing on-target off-tumor damage to normal oral epithelium and consistent with their lowest OM risk profile.

The elevated oral mucositis (OM) risk with combination regimens may reflect superimposed distinct mucosal injury pathways. Chemotherapy directly disrupts the oral epithelial barrier via DNA damage, oxidative stress and NF-κB-mediated inflammatory cascades (45); cetuximab blocks extracellular EGFR ligand binding and receptor activation (49); anti-angiogenic agents and multi-target TKIs delay mucosal re-epithelialization and wound closure by suppressing VEGF/VEGFR-dependent microangiogenesis and tissue repair (50, 51). These pathways combine with EGFR-TKI epithelial suppression to exacerbate mucosal damage. Given sparse direct comparative data and imprecise effect estimates, these mechanistic explanations remain speculative.

Our findings accord with prior pharmacovigilance studies demonstrating significant OM signals for second-generation EGFR-TKIs (52–54). However, spontaneous-reporting databases lack reliable exposure denominators and therefore cannot accurately estimate incidence. Existing network meta-analyses also indicated relatively high overall toxicity for afatinib and dacomitinib, but treated OM only as a secondary safety endpoint, without severity stratification or evaluation of combination regimens (55, 56). Our study addresses these limitations in three key aspects. First, only phase II/III randomized controlled trials were included, with systematic assessment of heterogeneity, inconsistency and robustness via sensitivity analyses. Second, a broader range of EGFR-TKI monotherapies and combination regimens were integrated into a unified evidence network. Third, any-grade and grade ≥3 OM were separately analyzed and quantitatively ranked by SUCRA, providing more granular evidence to inform risk stratification and clinical monitoring.

Recognition of oral mucositis (OM) risk is essential to pretreatment assessment and longitudinal safety monitoring of EGFR-TKI therapy, although regimen selection should remain guided by the driver mutation subtype, expected antitumor benefit, and overall toxicity profile (57, 58). Regimens showing higher-risk signals in the present study may warrant closer baseline oral assessment and monitoring, with attention to pre-existing oral lesions or infection, denture-related irritation, and impaired immune status (59–61). Current mucosal-injury guidelines and EGFR-TKI adverse-event consensus recommendations emphasize basic oral care, patient education, and early recognition; pain, erythema, ulceration, or impaired oral intake should prompt timely reporting, CTCAE-based grading, and exclusion of infection or alternative oral pathology. Grade 1 OM can generally be managed with local care without dose modification; persistent or intolerable grade 2 OM may warrant label-directed interruption or dose reduction with intensified symptomatic treatment; and grade ≥3 OM requires treatment interruption, enhanced analgesia, hydration or nutritional support, and management of concomitant infection, with dose-reduced rechallenge considered after recovery to grade ≤1 (57, 58, 62). These clinical recommendations are informed by existing guidelines and expert consensus rather than by the comparative estimates of this network meta-analysis.

Despite these clinical implications, this study has several limitations. First, OM was not a primary safety endpoint in most included trials, and suboptimal randomization and outcome assessment in some studies may introduce measurement and selective reporting bias. Second, evidence for several novel agents and combination regimens was sparse and largely indirect. Potential effect modifiers—including treatment line, geographic/population composition, EGFR mutation status, baseline patient characteristics, and treatment duration—vary across trials and may impair the comparability of some indirect comparisons. Pooling distinct chemotherapy backbones into a single node also cannot fully rule out differences in baseline OM risk across regimens. Such clinical heterogeneity may lead to residual between-study heterogeneity and uncertainty in the transitivity assumption, although inconsistency testing and sensitivity analyses showed broadly stable results. Finally, sparse grade ≥3 OM events across multiple nodes yielded wide 95% CrIs spanning the null, warranting cautious interpretation of effect estimates and SUCRA rankings.

## 5 Conclusions

OM risk varied substantially across EGFR-TKI regimens. Afatinib and dacomitinib showed higher-risk profiles for any-grade OM (vs chemo; CINeMA: high/moderate, respectively), while afatinib and mobocertinib showed prominent grade ≥3 signals (vs chemo; both moderate). Osimertinib and furmonertinib generally ranked lower. Several combination regimens also showed increased risk, particularly afatinib plus cetuximab (vs chemo; low/moderate for any-grade/grade ≥3 OM). These findings may inform risk stratification and oral monitoring but require confirmation in standardized prospective studies.

## Data Availability

The original contributions presented in the study are included in the article/**Supplementary Material**. Further inquiries can be directed to the corresponding authors.
