# Supplementary material for: Oral mucositis risk with EGFR tyrosine kinase inhibitor monotherapies and combination regimens in non-small cell lung cancer: a systematic review and network meta-analysis
Source: Front Immunol. 2026 Sep 18;17:1957825. doi: 10.3389/fimmu.2026.1957825 (PMC13631102; doi:10.3389/fimmu.2026.1957825)
Supplement: Supplementary file 1 [file DataSheet1.pdf]

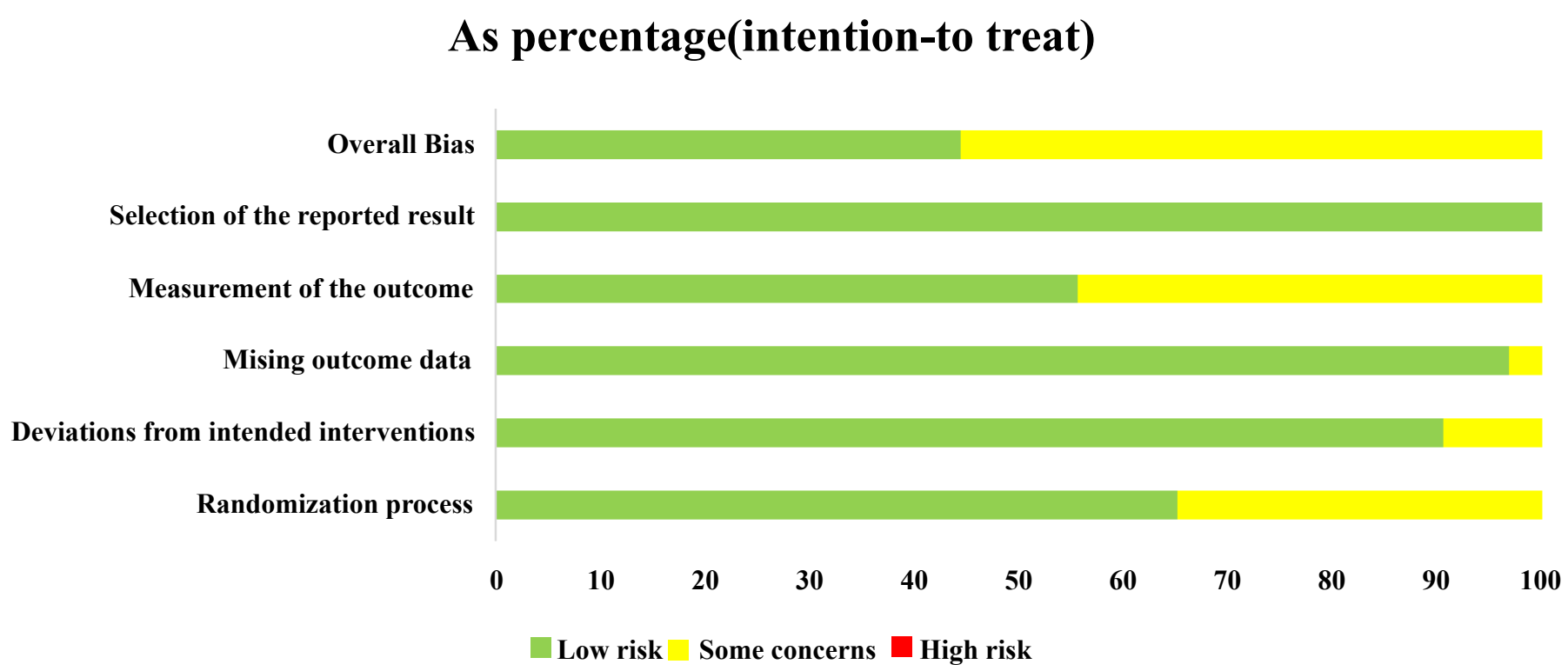


Risk of bias domains

| Study | D1 | D2 | D3 | D4 | D5 | Overall |
|---|---|---|---|---|---|---|
| Yi-Long Wu 2014 | + | + | + | + | + | + |
| Lecia V Sequist 2023 | + | + | + | + | + | + |
| Jean-Charles Soria 2015a | + | + | + | + | + | + |
| L Paz-Ares 2017 | ! | + | + | + | + | ! |
| Vincent A Mille 2012 | + | + | + | + | + | + |
| M Schuler 2016 | + | + | + | + | + | + |
| Shun Lu 2022 | + | + | + | + | + | + |
| Suresh S Ramalingam 2012 | ! | + | + | ! | + | ! |
| Suresh S Ramalingam 2014 | + | + | + | + | + | + |
| Yi-Long Wu 2017 | + | + | + | + | + | + |
| Peter M Ellis 2014 | + | + | + | + | + | + |
| Rogerio Lilenbaum 2008 | ! | + | + | ! | + | ! |
| Caicun Zhou 2011 | + | + | + | ! | + | ! |
| Karen Kelly 2012 | ! | + | + | + | + | ! |
| Tudor Ciuleanu 2012 | + | ! | + | + | + | ! |
| Y-L Wu 2015 | + | + | + | ! | + | ! |
| NCT01652469 | ! | + | + | ! | + | ! |
| NCT00126581 | ! | ! | + | ! | + | ! |
| Siow Ming Lee 2012 | + | + | + | + | + | + |
| Christian Dittrich 2014 | + | ! | + | ! | + | ! |
| Tony S K Mok 2009 | + | + | + | + | + | + |
| NCT01998919 | + | + | + | + | + | + |
| Yi-Long Wu 2013 | + | + | + | + | + | + |
| NCT00294762 | ! | ! | + | + | + | ! |
| Dae Ho Lee 2013 | + | + | + | ! | + | ! |
| Yuankai Shi 2022 | + | + | + | + | + | + |
| Riichiroh Maruyama 2008 | ! | + | + | + | + | ! |
| Edward S Kim 2008 | + | + | + | + | + | + |
| Tony S Mok 2009 | + | + | + | ! | + | ! |
| Tetsuya Mitsudomi 2010 | + | + | + | ! | + | ! |
| Dae Ho Lee 2010 | ! | ! | ! | ! | + | ! |
| Makoto Maemondo 2010 | ! | + | + | + | + | ! |
| Young Saing Kim 2016 | ! | + | + | ! | + | ! |
| Yukio Hosomi 2020 | ! | + | + | ! | + | ! |
| Nick Thatcher 2005 | + | + | + | + | + | + |

| Study | D1 | D2 | D3 | D4 | D5 | Overall |
|---|---|---|---|---|---|---|
| Hui Yu 2014 | ! | ! | ! | ! | + | ! |
| Jean-Charles Soria 2015b | + | + | + | + | + | + |
| Chaolun An 2016 | ! | + | + | + | + | ! |
| James Chih-Hsin Yang 2014 | + | + | + | ! | + | ! |
| Ying Cheng 2016 | + | + | + | ! | + | ! |
| Byoung Chul Cho 2023 | + | + | + | + | + | + |
| Pasi A Jänne 2025 | + | + | + | + | + | + |
| Tony S Mok 2017 | ! | + | + | ! | + | ! |
| N Peled 2025 | + | + | + | + | + | + |
| Kentaro Tanaka 2021 | + | + | + | ! | + | ! |
| David Planchard 2023 | + | + | + | ! | + | ! |
| NCT01196078 | ! | + | + | ! | + | ! |
| Takashi Ninomiya 2023 | ! | + | + | ! | + | ! |
| Sarah B Goldberg 2020 | + | + | + | ! | + | ! |
| Alexis B Cortot 2021 | + | + | + | ! | + | ! |
| Hua-Qiang Zhou 2024 | + | + | + | + | + | + |
| Joel W Neal 2017 | ! | + | + | ! | + | ! |
| Roy S Herbst 2011 | + | + | + | + | + | + |
| Haruhiro Saito 2019 | + | + | + | + | + | + |
| Youngjoo Lee 2023 | ! | + | + | ! | + | ! |
| Maria Carmela Piccirillo 2022 | + | + | + | + | + | + |
| Thomas E Stinchcomb 2019 | ! | + | + | ! | + | ! |
| R A Soo 2022 | ! | + | + | ! | + | ! |
| Hirotsugu Kenmots 2022 | + | + | + | + | + | + |
| Kazuhiko Nakagawa 2019 | + | + | + | + | + | + |
| H J M Groen 2013 | + | + | + | + | + | + |
| Giorgio V Scagliotti 2012 | + | + | + | + | + | + |
| Hye Ryun Kim 2017 | ! | + | + | ! | + | ! |

+ Low risk
! Some concerns
- High risk

D1 Randomisation process
D2 Deviations from the intended intervent
D3 Missing outcome data
D4 Measurement of the outcome
D5 Selection of the reported result

**Supplementary Figure S1. Risk-of-bias assessments of the included randomized controlled trials.**
