# Supplementary material for: Oral mucositis risk with EGFR tyrosine kinase inhibitor monotherapies and combination regimens in non-small cell lung cancer: a systematic review and network meta-analysis
Source: Front Immunol. 2026 Sep 18;17:1957825. doi: 10.3389/fimmu.2026.1957825 (PMC13631102; doi:10.3389/fimmu.2026.1957825)
Supplement: Supplementary file 2 [file DataSheet2.pdf]

Evaluation of the certainty of evidence using CINEMA framework for all-grade oral mucositis								
**Comparison**	**Number of studies**	**Within-study bias**	**Reporting bias**	**Indirectness**	**Imprecision**	**Heterogeneity**	**Incoherence**	**Confidence rating**
Afatinib:Afatinib+Bevacizumab	1	Some concerns	Low risk	No concerns	Major concerns	No concerns	Some concerns	**Very low**
Afatinib:Afatinib+Cetuximab	2	Some concerns	Low risk	No concerns	Major concerns	No concerns	Some concerns	**Very low**
Afatinib:Chemotherapy	2	Low risk	Low risk	No concerns	No concerns	No concerns	No concerns	**High**
Afatinib:Erlotinib	1	Low risk	Low risk	No concerns	No concerns	No concerns	No concerns	**High**
Afatinib:Gefitinib	1	Some concerns	Low risk	No concerns	No concerns	No concerns	No concerns	**Moderate**
Afatinib:Placebo	1	Low risk	Low risk	No concerns	No concerns	No concerns	No concerns	**High**
Afatinib+Chemotherapy:Chemotherapy	1	Low risk	Low risk	No concerns	Major concerns	No concerns	Some concerns	**Low**
Aumolertinib:Gefitinib	1	Low risk	Low risk	No concerns	Major concerns	No concerns	Some concerns	**Low**
Chemotherapy:Erlotinib	8	Some concerns	Low risk	No concerns	Major concerns	No concerns	Some concerns	**Very low**
Chemotherapy:Erlotinib+Chemotherapy	5	Some concerns	Low risk	No concerns	No concerns	Major concerns	Major concerns	**Very low**
Chemotherapy:Gefitinib	7	Some concerns	Low risk	No concerns	Major concerns	No concerns	No concerns	**Very low**
Chemotherapy:Gefitinib+Chemotherapy	2	Some concerns	Low risk	No concerns	No concerns	Major concerns	No concerns	**Very low**
Chemotherapy:Mobocertinib	1	Low risk	Low risk	No concerns	No concerns	Major concerns	Some concerns	**Low**
Chemotherapy:Osimertinib	1	Some concerns	Low risk	No concerns	Major concerns	No concerns	No concerns	**Very low**
Chemotherapy:Osimertinib+Chemotherapy	1	Some concerns	Low risk	No concerns	Major concerns	No concerns	No concerns	**Very low**
Dacomitinib:Erlotinib	2	Low risk	Low risk	No concerns	No concerns	Some concerns	No concerns	**Moderate**
Dacomitinib:Gefitinib	1	Low risk	Low risk	No concerns	No concerns	Major concerns	No concerns	**Low**
Dacomitinib:Placebo	1	Low risk	Low risk	No concerns	No concerns	No concerns	No concerns	**High**
Erlotinib:Erlotinib+Bevacizumab	5	Low risk	Low risk	No concerns	Some concerns	Some concerns	Some concerns	**Moderate**
Erlotinib:Erlotinib+Cabozantinib	1	Some concerns	Low risk	No concerns	Major concerns	No concerns	Some concerns	**Very low**
Erlotinib:Erlotinib+Chemotherapy	3	Some concerns	Low risk	No concerns	No concerns	Major concerns	No concerns	**Very low**
Erlotinib:Erlotinib+Ramucirumab	1	Low risk	Low risk	No concerns	Major concerns	No concerns	Some concerns	**Low**
Erlotinib:Erlotinib+Sunitinib	2	Low risk	Low risk	No concerns	Major concerns	No concerns	Some concerns	**Low**
Furmonertinib:Gefitinib	1	Some concerns	Low risk	No concerns	Major concerns	No concerns	Some concerns	**Very low**
Gefitinib:Gefitinib+Anlotinib	1	Low risk	Low risk	No concerns	Major concerns	No concerns	Some concerns	**Low**
Gefitinib:Gefitinib+Chemotherapy	3	Some concerns	Low risk	No concerns	Some concerns	Some concerns	No concerns	**Low**
Gefitinib:Gefitinib+Nimotuzumab	1	Some concerns	Low risk	No concerns	Major concerns	No concerns	Some concerns	**Very low**
Gefitinib:Lazertinib	1	Low risk	Low risk	No concerns	Major concerns	No concerns	Some concerns	**Low**
Gefitinib:Placebo	1	Low risk	Low risk	No concerns	No concerns	Some concerns	Major concerns	**Low**
Osimertinib:Osimertinib+Bevacizumab	2	Low risk	Low risk	No concerns	Major concerns	No concerns	Some concerns	**Low**
Osimertinib:Osimertinib+Chemotherapy	2	Some concerns	Low risk	No concerns	Major concerns	No concerns	No concerns	**Very low**
Afatinib:Afatinib+Chemotherapy	0	Low risk	Low risk	No concerns	Major concerns	No concerns	Some concerns	**Low**
Afatinib:Aumolertinib	0	Low risk	Low risk	No concerns	No concerns	Some concerns	Some concerns	**Moderate**
Afatinib:Dacomitinib	0	Low risk	Low risk	No concerns	Major concerns	No concerns	Some concerns	**Low**
Afatinib:Erlotinib+Bevacizumab	0	Low risk	Low risk	No concerns	No concerns	Major concerns	Some concerns	**Low**
Afatinib:Erlotinib+Cabozantinib	0	Some concerns	Low risk	No concerns	Major concerns	No concerns	Some concerns	**Very low**
Afatinib:Erlotinib+Chemotherapy	0	Low risk	Low risk	No concerns	No concerns	Major concerns	Some concerns	**Low**
Afatinib:Erlotinib+Ramucirumab	0	Low risk	Low risk	No concerns	No concerns	Major concerns	Some concerns	**Low**
Afatinib:Erlotinib+Sunitinib	0	Low risk	Low risk	No concerns	No concerns	Major concerns	Some concerns	**Low**
Afatinib:Furmonertinib	0	Some concerns	Low risk	No concerns	No concerns	No concerns	Some concerns	**Low**
Afatinib:Gefitinib+Anlotinib	0	Low risk	Low risk	No concerns	Major concerns	No concerns	Some concerns	**Low**
Afatinib:Gefitinib+Chemotherapy	0	Some concerns	Low risk	No concerns	No concerns	Major concerns	Some concerns	**Very low**
Afatinib:Gefitinib+Nimotuzumab	0	Some concerns	Low risk	No concerns	No concerns	No concerns	Some concerns	**Low**
Afatinib:Lazertinib	0	Low risk	Low risk	No concerns	Major concerns	No concerns	Some concerns	**Low**
Afatinib:Mobocertinib	0	Low risk	Low risk	No concerns	Major concerns	No concerns	Some concerns	**Low**
Afatinib:Osimertinib	0	Some concerns	Low risk	No concerns	No concerns	No concerns	Some concerns	**Low**
Afatinib:Osimertinib+Bevacizumab	0	Some concerns	Low risk	No concerns	Major concerns	No concerns	Some concerns	**Very low**
Afatinib:Osimertinib+Chemotherapy	0	Low risk	Low risk	No concerns	No concerns	Major concerns	Some concerns	**Low**
Afatinib+Bevacizumab:Afatinib+Cetuximab	0	Some concerns	Low risk	No concerns	Major concerns	No concerns	Some concerns	**Very low**
Afatinib+Bevacizumab:Afatinib+Chemotherapy	0	Low risk	Low risk	No concerns	Major concerns	No concerns	Some concerns	**Low**
Afatinib+Bevacizumab:Aumolertinib	0	Low risk	Low risk	No concerns	Major concerns	No concerns	Some concerns	**Low**
Afatinib+Bevacizumab:Chemotherapy	0	Some concerns	Low risk	No concerns	Some concerns	Some concerns	Some concerns	**Low**
Afatinib+Bevacizumab:Dacomitinib	0	Some concerns	Low risk	No concerns	Major concerns	No concerns	Some concerns	**Very low**
Afatinib+Bevacizumab:Erlotinib	0	Some concerns	Low risk	No concerns	Major concerns	No concerns	Some concerns	**Very low**
Afatinib+Bevacizumab:Erlotinib+Bevacizumab	0	Some concerns	Low risk	No concerns	Major concerns	No concerns	Some concerns	**Very low**
Afatinib+Bevacizumab:Erlotinib+Cabozantinib	0	Some concerns	Low risk	No concerns	Major concerns	No concerns	Some concerns	**Very low**
Afatinib+Bevacizumab:Erlotinib+Chemotherapy	0	Some concerns	Low risk	No concerns	Major concerns	No concerns	Some concerns	**Very low**
Afatinib+Bevacizumab:Erlotinib+Ramucirumab	0	Low risk	Low risk	No concerns	Major concerns	No concerns	Some concerns	**Low**
Afatinib+Bevacizumab:Erlotinib+Sunitinib	0	Low risk	Low risk	No concerns	Major concerns	No concerns	Some concerns	**Low**
Afatinib+Bevacizumab:Furmonertinib	0	Some concerns	Low risk	No concerns	Major concerns	No concerns	Some concerns	**Very low**
Afatinib+Bevacizumab:Gefitinib	0	Some concerns	Low risk	No concerns	Major concerns	No concerns	Some concerns	**Very low**
Afatinib+Bevacizumab:Gefitinib+Anlotinib	0	Low risk	Low risk	No concerns	Major concerns	No concerns	Some concerns	**Low**
Afatinib+Bevacizumab:Gefitinib+Chemotherapy	0	Some concerns	Low risk	No concerns	Major concerns	No concerns	Some concerns	**Very low**
Afatinib+Bevacizumab:Gefitinib+Nimotuzumab	0	Some concerns	Low risk	No concerns	Major concerns	No concerns	Some concerns	**Very low**
Afatinib+Bevacizumab:Lazertinib	0	Low risk	Low risk	No concerns	Major concerns	No concerns	Some concerns	**Low**
Afatinib+Bevacizumab:Mobocertinib	0	Low risk	Low risk	No concerns	Major concerns	No concerns	Some concerns	**Low**
Afatinib+Bevacizumab:Osimertinib	0	Some concerns	Low risk	No concerns	Major concerns	No concerns	Some concerns	**Very low**
Afatinib+Bevacizumab:Osimertinib+Bevacizumab	0	Some concerns	Low risk	No concerns	Major concerns	No concerns	Some concerns	**Very low**
Afatinib+Bevacizumab:Osimertinib+Chemotherapy	0	Some concerns	Low risk	No concerns	Major concerns	No concerns	Some concerns	**Very low**
Afatinib+Bevacizumab:Placebo	0	Low risk	Low risk	No concerns	No concerns	No concerns	Some concerns	**Moderate**
Afatinib+Cetuximab:Afatinib+Chemotherapy	0	Low risk	Low risk	No concerns	Major concerns	No concerns	Some concerns	**Low**
Afatinib+Cetuximab:Aumolertinib	0	Low risk	Low risk	No concerns	No concerns	Major concerns	Some concerns	**Low**
Afatinib+Cetuximab:Chemotherapy	0	Some concerns	Low risk	No concerns	No concerns	No concerns	Some concerns	**Low**
Afatinib+Cetuximab:Dacomitinib	0	Some concerns	Low risk	No concerns	Major concerns	No concerns	Some concerns	**Very low**
Afatinib+Cetuximab:Erlotinib	0	Some concerns	Low risk	No concerns	No concerns	No concerns	Some concerns	**Low**
Afatinib+Cetuximab:Erlotinib+Bevacizumab	0	Some concerns	Low risk	No concerns	No concerns	Major concerns	Some concerns	**Very low**
Afatinib+Cetuximab:Erlotinib+Cabozantinib	0	Some concerns	Low risk	No concerns	Major concerns	No concerns	Some concerns	**Very low**
Afatinib+Cetuximab:Erlotinib+Chemotherapy	0	Some concerns	Low risk	No concerns	No concerns	Major concerns	Some concerns	**Very low**
Afatinib+Cetuximab:Erlotinib+Ramucirumab	0	Low risk	Low risk	No concerns	No concerns	Major concerns	Some concerns	**Low**
Afatinib+Cetuximab:Erlotinib+Sunitinib	0	Low risk	Low risk	No concerns	No concerns	Major concerns	Some concerns	**Low**
Afatinib+Cetuximab:Furmonertinib	0	Some concerns	Low risk	No concerns	No concerns	No concerns	Some concerns	**Low**
Afatinib+Cetuximab:Gefitinib	0	Some concerns	Low risk	No concerns	No concerns	No concerns	Some concerns	**Low**
Afatinib+Cetuximab:Gefitinib+Anlotinib	0	Low risk	Low risk	No concerns	Major concerns	No concerns	Some concerns	**Low**
Afatinib+Cetuximab:Gefitinib+Chemotherapy	0	Some concerns	Low risk	No concerns	No concerns	Major concerns	Some concerns	**Very low**
Afatinib+Cetuximab:Gefitinib+Nimotuzumab	0	Some concerns	Low risk	No concerns	No concerns	Some concerns	Some concerns	**Low**
Afatinib+Cetuximab:Lazertinib	0	Low risk	Low risk	No concerns	Major concerns	No concerns	Some concerns	**Low**
Afatinib+Cetuximab:Mobocertinib	0	Low risk	Low risk	No concerns	Major concerns	No concerns	Some concerns	**Low**
Afatinib+Cetuximab:Osimertinib	0	Some concerns	Low risk	No concerns	No concerns	No concerns	Some concerns	**Low**
Afatinib+Cetuximab:Osimertinib+Bevacizumab	0	Some concerns	Low risk	No concerns	Major concerns	No concerns	Some concerns	**Very low**
Afatinib+Cetuximab:Osimertinib+Chemotherapy	0	Some concerns	Low risk	No concerns	No concerns	Major concerns	Some concerns	**Very low**

Afatinib+Cetuximab:Placebo	0	Low risk	Low risk	No concerns	No concerns	No concerns	Some concerns	**Moderate**
Afatinib+Chemotherapy:Aumolertinib	0	Low risk	Low risk	No concerns	Major concerns	No concerns	Some concerns	**Low**
Afatinib+Chemotherapy:Dacomitinib	0	Low risk	Low risk	No concerns	Major concerns	No concerns	Some concerns	**Low**
Afatinib+Chemotherapy:Erlotinib	0	Low risk	Low risk	No concerns	Major concerns	No concerns	Some concerns	**Low**
Afatinib+Chemotherapy:Erlotinib+Bevacizumab	0	Low risk	Low risk	No concerns	Major concerns	No concerns	Some concerns	**Low**
Afatinib+Chemotherapy:Erlotinib+Cabozantinib	0	Some concerns	Low risk	No concerns	Major concerns	No concerns	Some concerns	**Very low**
Afatinib+Chemotherapy:Erlotinib+Chemotherapy	0	Low risk	Low risk	No concerns	Major concerns	No concerns	Some concerns	**Low**
Afatinib+Chemotherapy:Erlotinib+Ramucirumab	0	Low risk	Low risk	No concerns	Major concerns	No concerns	Some concerns	**Low**
Afatinib+Chemotherapy:Erlotinib+Sunitinib	0	Low risk	Low risk	No concerns	Major concerns	No concerns	Some concerns	**Low**
Afatinib+Chemotherapy:Furmonertinib	0	Some concerns	Low risk	No concerns	Major concerns	No concerns	Some concerns	**Very low**
Afatinib+Chemotherapy:Gefitinib	0	Low risk	Low risk	No concerns	Major concerns	No concerns	Some concerns	**Low**
Afatinib+Chemotherapy:Gefitinib+Anlotinib	0	Low risk	Low risk	No concerns	Major concerns	No concerns	Some concerns	**Low**
Afatinib+Chemotherapy:Gefitinib+Chemotherapy	0	Low risk	Low risk	No concerns	Major concerns	No concerns	Some concerns	**Low**
Afatinib+Chemotherapy:Gefitinib+Nimotuzumab	0	Some concerns	Low risk	No concerns	Major concerns	No concerns	Some concerns	**Very low**
Afatinib+Chemotherapy:Lazertinib	0	Low risk	Low risk	No concerns	Major concerns	No concerns	Some concerns	**Low**
Afatinib+Chemotherapy:Mobocertinib	0	Low risk	Low risk	No concerns	Major concerns	No concerns	Some concerns	**Low**
Afatinib+Chemotherapy:Osimertinib	0	Low risk	Low risk	No concerns	Major concerns	No concerns	Some concerns	**Low**
Afatinib+Chemotherapy:Osimertinib+Bevacizumab	0	Low risk	Low risk	No concerns	Major concerns	No concerns	Some concerns	**Low**
Afatinib+Chemotherapy:Osimertinib+Chemotherapy	0	Low risk	Low risk	No concerns	Major concerns	No concerns	Some concerns	**Low**
Afatinib+Chemotherapy:Placebo	0	Low risk	Low risk	No concerns	No concerns	Some concerns	Some concerns	**Moderate**
Aumolertinib:Chemotherapy	0	Low risk	Low risk	No concerns	Major concerns	No concerns	Some concerns	**Low**
Aumolertinib:Dacomitinib	0	Low risk	Low risk	No concerns	Major concerns	No concerns	Some concerns	**Low**
Aumolertinib:Erlotinib	0	Low risk	Low risk	No concerns	Major concerns	No concerns	Some concerns	**Low**
Aumolertinib:Erlotinib+Bevacizumab	0	Low risk	Low risk	No concerns	Major concerns	No concerns	Some concerns	**Low**
Aumolertinib:Erlotinib+Cabozantinib	0	Some concerns	Low risk	No concerns	Major concerns	No concerns	Some concerns	**Very low**
Aumolertinib:Erlotinib+Chemotherapy	0	Low risk	Low risk	No concerns	Major concerns	No concerns	Some concerns	**Low**
Aumolertinib:Erlotinib+Ramucirumab	0	Low risk	Low risk	No concerns	Major concerns	No concerns	Some concerns	**Low**
Aumolertinib:Erlotinib+Sunitinib	0	Low risk	Low risk	No concerns	Major concerns	No concerns	Some concerns	**Low**
Aumolertinib:Furmonertinib	0	Some concerns	Low risk	No concerns	Major concerns	No concerns	Some concerns	**Very low**
Aumolertinib:Gefitinib+Anlotinib	0	Low risk	Low risk	No concerns	Major concerns	No concerns	Some concerns	**Low**
Aumolertinib:Gefitinib+Chemotherapy	0	Low risk	Low risk	No concerns	Major concerns	No concerns	Some concerns	**Low**
Aumolertinib:Gefitinib+Nimotuzumab	0	Some concerns	Low risk	No concerns	Major concerns	No concerns	Some concerns	**Very low**
Aumolertinib:Lazertinib	0	Low risk	Low risk	No concerns	Major concerns	No concerns	Some concerns	**Low**
Aumolertinib:Mobocertinib	0	Low risk	Low risk	No concerns	Major concerns	No concerns	Some concerns	**Low**
Aumolertinib:Osimertinib	0	Some concerns	Low risk	No concerns	Major concerns	No concerns	Some concerns	**Very low**
Aumolertinib:Osimertinib+Bevacizumab	0	Some concerns	Low risk	No concerns	Major concerns	No concerns	Some concerns	**Very low**
Aumolertinib:Osimertinib+Chemotherapy	0	Low risk	Low risk	No concerns	Major concerns	No concerns	Some concerns	**Low**
Aumolertinib:Placebo	0	Low risk	Low risk	No concerns	Some concerns	Some concerns	Some concerns	**Moderate**
Chemotherapy:Dacomitinib	0	Low risk	Low risk	No concerns	No concerns	Some concerns	Some concerns	**Moderate**
Chemotherapy:Erlotinib+Bevacizumab	0	Some concerns	Low risk	No concerns	Some concerns	Some concerns	Some concerns	**Low**
Chemotherapy:Erlotinib+Cabozantinib	0	Some concerns	Low risk	No concerns	Some concerns	Some concerns	Some concerns	**Low**
Chemotherapy:Erlotinib+Ramucirumab	0	Low risk	Low risk	No concerns	Major concerns	No concerns	Some concerns	**Low**
Chemotherapy:Erlotinib+Sunitinib	0	Low risk	Low risk	No concerns	Major concerns	No concerns	Some concerns	**Low**
Chemotherapy:Furmonertinib	0	Some concerns	Low risk	No concerns	Major concerns	No concerns	Some concerns	**Very low**
Chemotherapy:Gefitinib+Anlotinib	0	Low risk	Low risk	No concerns	Some concerns	Some concerns	Some concerns	**Moderate**
Chemotherapy:Gefitinib+Nimotuzumab	0	Some concerns	Low risk	No concerns	Major concerns	No concerns	Some concerns	**Very low**
Chemotherapy:Lazertinib	0	Low risk	Low risk	No concerns	Major concerns	No concerns	Some concerns	**Low**
Chemotherapy:Osimertinib+Bevacizumab	0	Some concerns	Low risk	No concerns	Major concerns	No concerns	Some concerns	**Very low**
Chemotherapy:Placebo	0	Low risk	Low risk	No concerns	No concerns	Major concerns	Some concerns	**Low**
Dacomitinib:Erlotinib+Bevacizumab	0	Low risk	Low risk	No concerns	Major concerns	No concerns	Some concerns	**Low**
Dacomitinib:Erlotinib+Cabozantinib	0	Some concerns	Low risk	No concerns	Major concerns	No concerns	Some concerns	**Very low**
Dacomitinib:Erlotinib+Chemotherapy	0	Some concerns	Low risk	No concerns	Major concerns	No concerns	Some concerns	**Very low**
Dacomitinib:Erlotinib+Ramucirumab	0	Low risk	Low risk	No concerns	Major concerns	No concerns	Some concerns	**Low**
Dacomitinib:Erlotinib+Sunitinib	0	Low risk	Low risk	No concerns	Major concerns	No concerns	Some concerns	**Low**
Dacomitinib:Furmonertinib	0	Some concerns	Low risk	No concerns	No concerns	Major concerns	Some concerns	**Very low**
Dacomitinib:Gefitinib+Anlotinib	0	Low risk	Low risk	No concerns	Major concerns	No concerns	Some concerns	**Low**
Dacomitinib:Gefitinib+Chemotherapy	0	Some concerns	Low risk	No concerns	Major concerns	No concerns	Some concerns	**Very low**
Dacomitinib:Gefitinib+Nimotuzumab	0	Some concerns	Low risk	No concerns	Some concerns	Some concerns	Some concerns	**Low**
Dacomitinib:Lazertinib	0	Low risk	Low risk	No concerns	Major concerns	No concerns	Some concerns	**Low**
Dacomitinib:Mobocertinib	0	Low risk	Low risk	No concerns	Major concerns	No concerns	Some concerns	**Low**
Dacomitinib:Osimertinib	0	Some concerns	Low risk	No concerns	No concerns	Major concerns	Some concerns	**Very low**
Dacomitinib:Osimertinib+Bevacizumab	0	Some concerns	Low risk	No concerns	Major concerns	No concerns	Some concerns	**Very low**
Dacomitinib:Osimertinib+Chemotherapy	0	Some concerns	Low risk	No concerns	Major concerns	No concerns	Some concerns	**Very low**
Erlotinib:Furmonertinib	0	Some concerns	Low risk	No concerns	Major concerns	No concerns	Some concerns	**Very low**
Erlotinib:Gefitinib	0	Some concerns	Low risk	No concerns	Major concerns	No concerns	Some concerns	**Very low**
Erlotinib:Gefitinib+Anlotinib	0	Low risk	Low risk	No concerns	Major concerns	No concerns	Some concerns	**Low**
Erlotinib:Gefitinib+Chemotherapy	0	Some concerns	Low risk	No concerns	Major concerns	No concerns	Some concerns	**Very low**
Erlotinib:Gefitinib+Nimotuzumab	0	Some concerns	Low risk	No concerns	Major concerns	No concerns	Some concerns	**Very low**
Erlotinib:Lazertinib	0	Low risk	Low risk	No concerns	Major concerns	No concerns	Some concerns	**Low**
Erlotinib:Mobocertinib	0	Low risk	Low risk	No concerns	Major concerns	No concerns	Some concerns	**Low**
Erlotinib:Osimertinib	0	Some concerns	Low risk	No concerns	Major concerns	No concerns	Some concerns	**Very low**
Erlotinib:Osimertinib+Bevacizumab	0	Some concerns	Low risk	No concerns	Major concerns	No concerns	Some concerns	**Very low**
Erlotinib:Osimertinib+Chemotherapy	0	Some concerns	Low risk	No concerns	Major concerns	No concerns	Some concerns	**Very low**
Erlotinib:Placebo	0	Low risk	Low risk	No concerns	No concerns	Some concerns	Some concerns	**Moderate**
Erlotinib+Bevacizumab:Erlotinib+Cabozantinib	0	Some concerns	Low risk	No concerns	Major concerns	No concerns	Some concerns	**Very low**
Erlotinib+Bevacizumab:Erlotinib+Chemotherapy	0	Some concerns	Low risk	No concerns	Major concerns	No concerns	Some concerns	**Very low**
Erlotinib+Bevacizumab:Erlotinib+Ramucirumab	0	Low risk	Low risk	No concerns	Major concerns	No concerns	Some concerns	**Low**
Erlotinib+Bevacizumab:Erlotinib+Sunitinib	0	Low risk	Low risk	No concerns	Major concerns	No concerns	Some concerns	**Low**
Erlotinib+Bevacizumab:Furmonertinib	0	Some concerns	Low risk	No concerns	Major concerns	No concerns	Some concerns	**Very low**
Erlotinib+Bevacizumab:Gefitinib	0	Some concerns	Low risk	No concerns	Major concerns	No concerns	Some concerns	**Very low**
Erlotinib+Bevacizumab:Gefitinib+Anlotinib	0	Low risk	Low risk	No concerns	Major concerns	No concerns	Some concerns	**Low**
Erlotinib+Bevacizumab:Gefitinib+Chemotherapy	0	Some concerns	Low risk	No concerns	Major concerns	No concerns	Some concerns	**Very low**
Erlotinib+Bevacizumab:Gefitinib+Nimotuzumab	0	Some concerns	Low risk	No concerns	Major concerns	No concerns	Some concerns	**Very low**
Erlotinib+Bevacizumab:Lazertinib	0	Low risk	Low risk	No concerns	Major concerns	No concerns	Some concerns	**Low**
Erlotinib+Bevacizumab:Mobocertinib	0	Low risk	Low risk	No concerns	Major concerns	No concerns	Some concerns	**Low**
Erlotinib+Bevacizumab:Osimertinib	0	Some concerns	Low risk	No concerns	Major concerns	No concerns	Some concerns	**Very low**
Erlotinib+Bevacizumab:Osimertinib+Bevacizumab	0	Some concerns	Low risk	No concerns	Major concerns	No concerns	Some concerns	**Very low**
Erlotinib+Bevacizumab:Osimertinib+Chemotherapy	0	Some concerns	Low risk	No concerns	Major concerns	No concerns	Some concerns	**Very low**
Erlotinib+Bevacizumab:Placebo	0	Low risk	Low risk	No concerns	No concerns	No concerns	Some concerns	**Moderate**
Erlotinib+Cabozantinib:Erlotinib+Chemotherapy	0	Some concerns	Low risk	No concerns	Major concerns	No concerns	Some concerns	**Very low**
Erlotinib+Cabozantinib:Erlotinib+Ramucirumab	0	Some concerns	Low risk	No concerns	Major concerns	No concerns	Some concerns	**Very low**
Erlotinib+Cabozantinib:Erlotinib+Sunitinib	0	Some concerns	Low risk	No concerns	Major concerns	No concerns	Some concerns	**Very low**

Erlotinib+Cabozantinib:Furmonertinib	0	Some concerns	Low risk	No concerns	Major concerns	No concerns	Some concerns	Very low
Erlotinib+Cabozantinib:Gefitinib	0	Some concerns	Low risk	No concerns	Major concerns	No concerns	Some concerns	Very low
Erlotinib+Cabozantinib:Gefitinib+Anlotinib	0	Some concerns	Low risk	No concerns	Major concerns	No concerns	Some concerns	Very low
Erlotinib+Cabozantinib:Gefitinib+Chemotherapy	0	Some concerns	Low risk	No concerns	Major concerns	No concerns	Some concerns	Very low
Erlotinib+Cabozantinib:Gefitinib+Nimotuzumab	0	Some concerns	Low risk	No concerns	Major concerns	No concerns	Some concerns	Very low
Erlotinib+Cabozantinib:Lazertinib	0	Some concerns	Low risk	No concerns	Major concerns	No concerns	Some concerns	Very low
Erlotinib+Cabozantinib:Mobocertinib	0	Some concerns	Low risk	No concerns	Major concerns	No concerns	Some concerns	Very low
Erlotinib+Cabozantinib:Osimertinib	0	Some concerns	Low risk	No concerns	Major concerns	No concerns	Some concerns	Very low
Erlotinib+Cabozantinib:Osimertinib+Bevacizumab	0	Some concerns	Low risk	No concerns	Major concerns	No concerns	Some concerns	Very low
Erlotinib+Cabozantinib:Osimertinib+Chemotherapy	0	Some concerns	Low risk	No concerns	Major concerns	No concerns	Some concerns	Very low
Erlotinib+Cabozantinib:Placebo	0	Some concerns	Low risk	No concerns	No concerns	No concerns	Some concerns	Low
Erlotinib+Chemotherapy:Erlotinib+Ramucirumab	0	Low risk	Low risk	No concerns	Major concerns	No concerns	Some concerns	Low
Erlotinib+Chemotherapy:Erlotinib+Sunitinib	0	Low risk	Low risk	No concerns	Major concerns	No concerns	Some concerns	Low
Erlotinib+Chemotherapy:Furmonertinib	0	Some concerns	Low risk	No concerns	Major concerns	No concerns	Some concerns	Very low
Erlotinib+Chemotherapy:Gefitinib	0	Some concerns	Low risk	No concerns	Some concerns	Some concerns	Some concerns	Low
Erlotinib+Chemotherapy:Gefitinib+Anlotinib	0	Low risk	Low risk	No concerns	Major concerns	No concerns	Some concerns	Low
Erlotinib+Chemotherapy:Gefitinib+Chemotherapy	0	Some concerns	Low risk	No concerns	Major concerns	No concerns	Some concerns	Very low
Erlotinib+Chemotherapy:Gefitinib+Nimotuzumab	0	Some concerns	Low risk	No concerns	Major concerns	No concerns	Some concerns	Very low
Erlotinib+Chemotherapy:Lazertinib	0	Low risk	Low risk	No concerns	Major concerns	No concerns	Some concerns	Low
Erlotinib+Chemotherapy:Mobocertinib	0	Low risk	Low risk	No concerns	Major concerns	No concerns	Some concerns	Low
Erlotinib+Chemotherapy:Osimertinib	0	Some concerns	Low risk	No concerns	Major concerns	No concerns	Some concerns	Very low
Erlotinib+Chemotherapy:Osimertinib+Bevacizumab	0	Some concerns	Low risk	No concerns	Major concerns	No concerns	Some concerns	Very low
Erlotinib+Chemotherapy:Osimertinib+Chemotherapy	0	Some concerns	Low risk	No concerns	Major concerns	No concerns	Some concerns	Very low
Erlotinib+Chemotherapy:Placebo	0	Low risk	Low risk	No concerns	No concerns	No concerns	Some concerns	Moderate
Erlotinib+Ramucirumab:Erlotinib+Sunitinib	0	Low risk	Low risk	No concerns	Major concerns	No concerns	Some concerns	Low
Erlotinib+Ramucirumab:Furmonertinib	0	Some concerns	Low risk	No concerns	Major concerns	No concerns	Some concerns	Very low
Erlotinib+Ramucirumab:Gefitinib	0	Low risk	Low risk	No concerns	Major concerns	No concerns	Some concerns	Low
Erlotinib+Ramucirumab:Gefitinib+Anlotinib	0	Low risk	Low risk	No concerns	Major concerns	No concerns	Some concerns	Low
Erlotinib+Ramucirumab:Gefitinib+Chemotherapy	0	Some concerns	Low risk	No concerns	Major concerns	No concerns	Some concerns	Very low
Erlotinib+Ramucirumab:Gefitinib+Nimotuzumab	0	Some concerns	Low risk	No concerns	Major concerns	No concerns	Some concerns	Very low
Erlotinib+Ramucirumab:Lazertinib	0	Low risk	Low risk	No concerns	Major concerns	No concerns	Some concerns	Low
Erlotinib+Ramucirumab:Mobocertinib	0	Low risk	Low risk	No concerns	Major concerns	No concerns	Some concerns	Low
Erlotinib+Ramucirumab:Osimertinib	0	Some concerns	Low risk	No concerns	Major concerns	No concerns	Some concerns	Very low
Erlotinib+Ramucirumab:Osimertinib+Bevacizumab	0	Some concerns	Low risk	No concerns	Major concerns	No concerns	Some concerns	Very low
Erlotinib+Ramucirumab:Osimertinib+Chemotherapy	0	Low risk	Low risk	No concerns	Major concerns	No concerns	Some concerns	Low
Erlotinib+Ramucirumab:Placebo	0	Low risk	Low risk	No concerns	No concerns	Major concerns	Some concerns	Low
Erlotinib+Sunitinib:Furmonertinib	0	Some concerns	Low risk	No concerns	Major concerns	No concerns	Some concerns	Very low
Erlotinib+Sunitinib:Gefitinib	0	Low risk	Low risk	No concerns	Major concerns	No concerns	Some concerns	Low
Erlotinib+Sunitinib:Gefitinib+Anlotinib	0	Low risk	Low risk	No concerns	Major concerns	No concerns	Some concerns	Low
Erlotinib+Sunitinib:Gefitinib+Chemotherapy	0	Some concerns	Low risk	No concerns	Major concerns	No concerns	Some concerns	Very low
Erlotinib+Sunitinib:Gefitinib+Nimotuzumab	0	Some concerns	Low risk	No concerns	Major concerns	No concerns	Some concerns	Very low
Erlotinib+Sunitinib:Lazertinib	0	Low risk	Low risk	No concerns	Major concerns	No concerns	Some concerns	Low
Erlotinib+Sunitinib:Mobocertinib	0	Low risk	Low risk	No concerns	Major concerns	No concerns	Some concerns	Low
Erlotinib+Sunitinib:Osimertinib	0	Some concerns	Low risk	No concerns	Major concerns	No concerns	Some concerns	Very low
Erlotinib+Sunitinib:Osimertinib+Bevacizumab	0	Some concerns	Low risk	No concerns	Major concerns	No concerns	Some concerns	Very low
Erlotinib+Sunitinib:Osimertinib+Chemotherapy	0	Low risk	Low risk	No concerns	Major concerns	No concerns	Some concerns	Low
Erlotinib+Sunitinib:Placebo	0	Low risk	Low risk	No concerns	No concerns	Some concerns	Some concerns	Moderate
Furmonertinib:Gefitinib+Anlotinib	0	Some concerns	Low risk	No concerns	Major concerns	No concerns	Some concerns	Very low
Furmonertinib:Gefitinib+Chemotherapy	0	Some concerns	Low risk	No concerns	Major concerns	No concerns	Some concerns	Very low
Furmonertinib:Gefitinib+Nimotuzumab	0	Some concerns	Low risk	No concerns	Major concerns	No concerns	Some concerns	Very low
Furmonertinib:Lazertinib	0	Some concerns	Low risk	No concerns	Major concerns	No concerns	Some concerns	Very low
Furmonertinib:Mobocertinib	0	Some concerns	Low risk	No concerns	Major concerns	No concerns	Some concerns	Very low
Furmonertinib:Osimertinib	0	Some concerns	Low risk	No concerns	Major concerns	No concerns	Some concerns	Very low
Furmonertinib:Osimertinib+Bevacizumab	0	Some concerns	Low risk	No concerns	Major concerns	No concerns	Some concerns	Very low
Furmonertinib:Osimertinib+Chemotherapy	0	Some concerns	Low risk	No concerns	Major concerns	No concerns	Some concerns	Very low
Furmonertinib:Placebo	0	Some concerns	Low risk	No concerns	Major concerns	No concerns	Some concerns	Very low
Gefitinib:Mobocertinib	0	Low risk	Low risk	No concerns	Some concerns	Some concerns	Some concerns	Moderate
Gefitinib:Osimertinib	0	Some concerns	Low risk	No concerns	Major concerns	No concerns	Some concerns	Very low
Gefitinib:Osimertinib+Bevacizumab	0	Some concerns	Low risk	No concerns	Major concerns	No concerns	Some concerns	Very low
Gefitinib:Osimertinib+Chemotherapy	0	Some concerns	Low risk	No concerns	Major concerns	No concerns	Some concerns	Very low
Gefitinib+Anlotinib:Gefitinib+Chemotherapy	0	Low risk	Low risk	No concerns	Major concerns	No concerns	Some concerns	Low
Gefitinib+Anlotinib:Gefitinib+Nimotuzumab	0	Some concerns	Low risk	No concerns	Major concerns	No concerns	Some concerns	Very low
Gefitinib+Anlotinib:Lazertinib	0	Low risk	Low risk	No concerns	Major concerns	No concerns	Some concerns	Low
Gefitinib+Anlotinib:Mobocertinib	0	Low risk	Low risk	No concerns	Major concerns	No concerns	Some concerns	Low
Gefitinib+Anlotinib:Osimertinib	0	Some concerns	Low risk	No concerns	Major concerns	No concerns	Some concerns	Very low
Gefitinib+Anlotinib:Osimertinib+Bevacizumab	0	Some concerns	Low risk	No concerns	Major concerns	No concerns	Some concerns	Very low
Gefitinib+Anlotinib:Osimertinib+Chemotherapy	0	Low risk	Low risk	No concerns	Major concerns	No concerns	Some concerns	Low
Gefitinib+Anlotinib:Placebo	0	Low risk	Low risk	No concerns	No concerns	No concerns	Some concerns	Moderate
Gefitinib+Chemotherapy:Gefitinib+Nimotuzumab	0	Some concerns	Low risk	No concerns	Major concerns	No concerns	Some concerns	Very low
Gefitinib+Chemotherapy:Lazertinib	0	Low risk	Low risk	No concerns	Major concerns	No concerns	Some concerns	Low
Gefitinib+Chemotherapy:Mobocertinib	0	Low risk	Low risk	No concerns	Major concerns	No concerns	Some concerns	Low
Gefitinib+Chemotherapy:Osimertinib	0	Some concerns	Low risk	No concerns	Major concerns	No concerns	Some concerns	Very low
Gefitinib+Chemotherapy:Osimertinib+Bevacizumab	0	Some concerns	Low risk	No concerns	Major concerns	No concerns	Some concerns	Very low
Gefitinib+Chemotherapy:Osimertinib+Chemotherapy	0	Some concerns	Low risk	No concerns	Major concerns	No concerns	Some concerns	Very low
Gefitinib+Chemotherapy:Placebo	0	Low risk	Low risk	No concerns	No concerns	No concerns	Some concerns	Moderate
Gefitinib+Nimotuzumab:Lazertinib	0	Some concerns	Low risk	No concerns	Major concerns	No concerns	Some concerns	Very low
Gefitinib+Nimotuzumab:Mobocertinib	0	Some concerns	Low risk	No concerns	Major concerns	No concerns	Some concerns	Very low
Gefitinib+Nimotuzumab:Osimertinib	0	Some concerns	Low risk	No concerns	Major concerns	No concerns	Some concerns	Very low
Gefitinib+Nimotuzumab:Osimertinib+Bevacizumab	0	Some concerns	Low risk	No concerns	Major concerns	No concerns	Some concerns	Very low
Gefitinib+Nimotuzumab:Osimertinib+Chemotherapy	0	Some concerns	Low risk	No concerns	Major concerns	No concerns	Some concerns	Very low
Gefitinib+Nimotuzumab:Placebo	0	Some concerns	Low risk	No concerns	Major concerns	No concerns	Some concerns	Very low
Lazertinib:Mobocertinib	0	Low risk	Low risk	No concerns	Major concerns	No concerns	Some concerns	Low
Lazertinib:Osimertinib	0	Some concerns	Low risk	No concerns	Major concerns	No concerns	Some concerns	Very low
Lazertinib:Osimertinib+Bevacizumab	0	Some concerns	Low risk	No concerns	Major concerns	No concerns	Some concerns	Very low
Lazertinib:Osimertinib+Chemotherapy	0	Low risk	Low risk	No concerns	Major concerns	No concerns	Some concerns	Low
Lazertinib:Placebo	0	Low risk	Low risk	No concerns	No concerns	No concerns	Some concerns	Moderate
Mobocertinib:Osimertinib	0	Low risk	Low risk	No concerns	Major concerns	No concerns	Some concerns	Low
Mobocertinib:Osimertinib+Bevacizumab	0	Low risk	Low risk	No concerns	Major concerns	No concerns	Some concerns	Low
Mobocertinib:Osimertinib+Chemotherapy	0	Low risk	Low risk	No concerns	Major concerns	No concerns	Some concerns	Low
Mobocertinib:Placebo	0	Low risk	Low risk	No concerns	No concerns	No concerns	Some concerns	Moderate
Osimertinib:Placebo	0	Some concerns	Low risk	No concerns	Major concerns	No concerns	Some concerns	Very low
Osimertinib+Bevacizumab:Osimertinib+Chemotherapy	0	Some concerns	Low risk	No concerns	Major concerns	No concerns	Some concerns	Very low

Osimertinib+Bevacizumab:Placebo	0	Some concerns	Low risk	No concerns	No concerns	Major concerns	Some concerns	**Very low**
Osimertinib+Chemotherapy:Placebo	0	Low risk	Low risk	No concerns	No concerns	Major concerns	Some concerns	**Low**

Evaluation of the certainty of evidence using CINEMA framework for grade $\geq$3 oral mucositis

Comparison	Number of studies	Within-study bias	Reporting bias	Indirectness	Imprecision	Heterogeneity	Incoherence	Confidence rating
Afatinib:Afatinib+Bevacizumab	1	Some concerns	Low risk	No concerns	Major concerns	No concerns	No concerns	**Very low**
Afatinib:Afatinib+Cetuximab	2	Some concerns	Low risk	No concerns	Major concerns	No concerns	No concerns	**Very low**
Afatinib:Chemotherapy	1	Low risk	Low risk	No concerns	Some concerns	No concerns	No concerns	**Moderate**
Afatinib:Erlotinib	1	Low risk	Low risk	No concerns	No concerns	No concerns	No concerns	**High**
Afatinib:Gefitinib	1	Some concerns	Low risk	No concerns	No concerns	No concerns	No concerns	**Moderate**
Afatinib:Placebo	1	Low risk	Low risk	No concerns	No concerns	No concerns	No concerns	**High**
Afatinib+Chemotherapy:Chemotherapy	1	Low risk	Low risk	No concerns	Major concerns	No concerns	No concerns	**Low**
Chemotherapy:Erlotinib	5	Some concerns	Low risk	No concerns	Major concerns	No concerns	No concerns	**Very low**
Chemotherapy:Erlotinib+Chemotherapy	2	Some concerns	Low risk	No concerns	Major concerns	No concerns	No concerns	**Very low**
Chemotherapy:Gefitinib	4	Some concerns	Low risk	No concerns	Major concerns	No concerns	No concerns	**Very low**
Chemotherapy:Gefitinib+Chemotherapy	2	Some concerns	Low risk	No concerns	Major concerns	No concerns	No concerns	**Very low**
Chemotherapy:Mobocertinib	1	Low risk	Low risk	No concerns	Some concerns	Some concerns	No concerns	**Moderate**
Chemotherapy:Osimertinib	1	Some concerns	Low risk	No concerns	Major concerns	No concerns	No concerns	**Very low**
Dacomitinib:Erlotinib	2	Low risk	Low risk	No concerns	No concerns	No concerns	No concerns	**High**
Dacomitinib:Gefitinib	1	Low risk	Low risk	No concerns	No concerns	No concerns	No concerns	**High**
Dacomitinib:Placebo	1	Low risk	Low risk	No concerns	No concerns	No concerns	No concerns	**High**
Erlotinib:Erlotinib+Bevacizumab	3	Low risk	Low risk	No concerns	Major concerns	No concerns	No concerns	**Low**
Erlotinib:Erlotinib+Cabozantinib	1	Some concerns	Low risk	No concerns	Major concerns	No concerns	No concerns	**Very low**
Erlotinib:Erlotinib+Chemotherapy	2	Some concerns	Low risk	No concerns	Major concerns	No concerns	No concerns	**Very low**
Erlotinib:Erlotinib+Ramucirumab	1	Low risk	Low risk	No concerns	Some concerns	No concerns	No concerns	**Moderate**
Erlotinib:Placebo	1	Low risk	Low risk	No concerns	Major concerns	No concerns	No concerns	**Low**
Furmonertinib:Gefitinib	1	Low risk	Low risk	No concerns	Major concerns	No concerns	No concerns	**Low**
Gefitinib:Gefitinib+Anlotinib	1	Low risk	Low risk	No concerns	Major concerns	No concerns	No concerns	**Low**
Gefitinib:Gefitinib+Chemotherapy	2	Some concerns	Low risk	No concerns	Some concerns	No concerns	No concerns	**Low**
Gefitinib:Placebo	1	Low risk	Low risk	No concerns	Major concerns	No concerns	No concerns	**Low**
Osimertinib:Osimertinib+Bevacizumab	2	Low risk	Low risk	No concerns	Major concerns	No concerns	No concerns	**Low**
Osimertinib:Osimertinib+Chemotherapy	2	Some concerns	Low risk	No concerns	Major concerns	No concerns	No concerns	**Very low**
Afatinib:Afatinib+Chemotherapy	0	Low risk	Low risk	No concerns	Major concerns	No concerns	No concerns	**Low**
Afatinib:Dacomitinib	0	Low risk	Low risk	No concerns	Major concerns	No concerns	No concerns	**Low**
Afatinib:Erlotinib+Bevacizumab	0	Low risk	Low risk	No concerns	Major concerns	No concerns	No concerns	**Low**
Afatinib:Erlotinib+Cabozantinib	0	Some concerns	Low risk	No concerns	Major concerns	No concerns	No concerns	**Very low**
Afatinib:Erlotinib+Chemotherapy	0	Some concerns	Low risk	No concerns	Major concerns	No concerns	No concerns	**Very low**
Afatinib:Erlotinib+Ramucirumab	0	Low risk	Low risk	No concerns	No concerns	No concerns	No concerns	**High**
Afatinib:Furmonertinib	0	Low risk	Low risk	No concerns	Some concerns	No concerns	No concerns	**Moderate**
Afatinib:Gefitinib+Anlotinib	0	Low risk	Low risk	No concerns	Major concerns	No concerns	No concerns	**Low**
Afatinib:Gefitinib+Chemotherapy	0	Some concerns	Low risk	No concerns	Major concerns	No concerns	No concerns	**Very low**
Afatinib:Mobocertinib	0	Low risk	Low risk	No concerns	Major concerns	No concerns	No concerns	**Low**
Afatinib:Osimertinib	0	Some concerns	Low risk	No concerns	No concerns	No concerns	No concerns	**Moderate**
Afatinib:Osimertinib+Bevacizumab	0	Some concerns	Low risk	No concerns	Major concerns	No concerns	No concerns	**Very low**
Afatinib:Osimertinib+Chemotherapy	0	Some concerns	Low risk	No concerns	Major concerns	No concerns	No concerns	**Very low**
Afatinib+Bevacizumab:Afatinib+Cetuximab	0	Some concerns	Low risk	No concerns	Major concerns	No concerns	No concerns	**Very low**
Afatinib+Bevacizumab:Afatinib+Chemotherapy	0	Low risk	Low risk	No concerns	Major concerns	No concerns	No concerns	**Low**
Afatinib+Bevacizumab:Chemotherapy	0	Some concerns	Low risk	No concerns	Major concerns	No concerns	No concerns	**Very low**
Afatinib+Bevacizumab:Dacomitinib	0	Low risk	Low risk	No concerns	Major concerns	No concerns	No concerns	**Low**
Afatinib+Bevacizumab:Erlotinib	0	Some concerns	Low risk	No concerns	No concerns	No concerns	No concerns	**Moderate**
Afatinib+Bevacizumab:Erlotinib+Bevacizumab	0	Low risk	Low risk	No concerns	Major concerns	No concerns	No concerns	**Low**
Afatinib+Bevacizumab:Erlotinib+Cabozantinib	0	Some concerns	Low risk	No concerns	Major concerns	No concerns	No concerns	**Very low**
Afatinib+Bevacizumab:Erlotinib+Chemotherapy	0	Some concerns	Low risk	No concerns	Major concerns	No concerns	No concerns	**Very low**
Afatinib+Bevacizumab:Erlotinib+Ramucirumab	0	Low risk	Low risk	No concerns	Some concerns	No concerns	No concerns	**Moderate**
Afatinib+Bevacizumab:Furmonertinib	0	Low risk	Low risk	No concerns	Some concerns	Some concerns	No concerns	**Moderate**
Afatinib+Bevacizumab:Gefitinib	0	Some concerns	Low risk	No concerns	No concerns	No concerns	No concerns	**Moderate**
Afatinib+Bevacizumab:Gefitinib+Anlotinib	0	Low risk	Low risk	No concerns	Major concerns	No concerns	No concerns	**Low**
Afatinib+Bevacizumab:Gefitinib+Chemotherapy	0	Some concerns	Low risk	No concerns	Major concerns	No concerns	No concerns	**Very low**
Afatinib+Bevacizumab:Mobocertinib	0	Low risk	Low risk	No concerns	Major concerns	No concerns	No concerns	**Low**
Afatinib+Bevacizumab:Osimertinib	0	Some concerns	Low risk	No concerns	No concerns	No concerns	No concerns	**Moderate**
Afatinib+Bevacizumab:Osimertinib+Bevacizumab	0	Some concerns	Low risk	No concerns	Major concerns	No concerns	No concerns	**Very low**
Afatinib+Bevacizumab:Osimertinib+Chemotherapy	0	Some concerns	Low risk	No concerns	Major concerns	No concerns	No concerns	**Very low**
Afatinib+Bevacizumab:Placebo	0	Low risk	Low risk	No concerns	No concerns	No concerns	No concerns	**High**
Afatinib+Cetuximab:Afatinib+Chemotherapy	0	Low risk	Low risk	No concerns	Major concerns	No concerns	No concerns	**Low**
Afatinib+Cetuximab:Chemotherapy	0	Some concerns	Low risk	No concerns	No concerns	No concerns	No concerns	**Moderate**
Afatinib+Cetuximab:Dacomitinib	0	Low risk	Low risk	No concerns	Major concerns	No concerns	No concerns	**Low**
Afatinib+Cetuximab:Erlotinib	0	Some concerns	Low risk	No concerns	No concerns	No concerns	No concerns	**Moderate**
Afatinib+Cetuximab:Erlotinib+Bevacizumab	0	Low risk	Low risk	No concerns	No concerns	Some concerns	No concerns	**Moderate**
Afatinib+Cetuximab:Erlotinib+Cabozantinib	0	Some concerns	Low risk	No concerns	Major concerns	No concerns	No concerns	**Very low**
Afatinib+Cetuximab:Erlotinib+Chemotherapy	0	Some concerns	Low risk	No concerns	No concerns	Some concerns	No concerns	**Low**
Afatinib+Cetuximab:Erlotinib+Ramucirumab	0	Low risk	Low risk	No concerns	No concerns	No concerns	No concerns	**High**
Afatinib+Cetuximab:Furmonertinib	0	Low risk	Low risk	No concerns	No concerns	No concerns	No concerns	**High**
Afatinib+Cetuximab:Gefitinib	0	Some concerns	Low risk	No concerns	No concerns	No concerns	No concerns	**Moderate**
Afatinib+Cetuximab:Gefitinib+Anlotinib	0	Low risk	Low risk	No concerns	Major concerns	No concerns	No concerns	**Low**
Afatinib+Cetuximab:Gefitinib+Chemotherapy	0	Some concerns	Low risk	No concerns	Major concerns	No concerns	No concerns	**Very low**
Afatinib+Cetuximab:Mobocertinib	0	Low risk	Low risk	No concerns	Major concerns	No concerns	No concerns	**Low**
Afatinib+Cetuximab:Osimertinib	0	Some concerns	Low risk	No concerns	No concerns	No concerns	No concerns	**Moderate**
Afatinib+Cetuximab:Osimertinib+Bevacizumab	0	Some concerns	Low risk	No concerns	Some concerns	Some concerns	No concerns	**Low**
Afatinib+Cetuximab:Osimertinib+Chemotherapy	0	Some concerns	Low risk	No concerns	Some concerns	Some concerns	No concerns	**Low**
Afatinib+Cetuximab:Placebo	0	Low risk	Low risk	No concerns	No concerns	No concerns	No concerns	**High**
Afatinib+Chemotherapy:Dacomitinib	0	Low risk	Low risk	No concerns	Major concerns	No concerns	No concerns	**Low**
Afatinib+Chemotherapy:Erlotinib	0	Low risk	Low risk	No concerns	Major concerns	No concerns	No concerns	**Low**
Afatinib+Chemotherapy:Erlotinib+Bevacizumab	0	Low risk	Low risk	No concerns	Major concerns	No concerns	No concerns	**Low**
Afatinib+Chemotherapy:Erlotinib+Cabozantinib	0	Some concerns	Low risk	No concerns	Major concerns	No concerns	No concerns	**Very low**
Afatinib+Chemotherapy:Erlotinib+Chemotherapy	0	Some concerns	Low risk	No concerns	Major concerns	No concerns	No concerns	**Very low**
Afatinib+Chemotherapy:Erlotinib+Ramucirumab	0	Low risk	Low risk	No concerns	Major concerns	No concerns	No concerns	**Low**
Afatinib+Chemotherapy:Furmonertinib	0	Low risk	Low risk	No concerns	Major concerns	No concerns	No concerns	**Low**
Afatinib+Chemotherapy:Gefitinib	0	Low risk	Low risk	No concerns	Major concerns	No concerns	No concerns	**Low**
Afatinib+Chemotherapy:Gefitinib+Anlotinib	0	Low risk	Low risk	No concerns	Major concerns	No concerns	No concerns	**Low**
Afatinib+Chemotherapy:Gefitinib+Chemotherapy	0	Low risk	Low risk	No concerns	Major concerns	No concerns	No concerns	**Low**
Afatinib+Chemotherapy:Mobocertinib	0	Low risk	Low risk	No concerns	Major concerns	No concerns	No concerns	**Low**
Afatinib+Chemotherapy:Osimertinib	0	Some concerns	Low risk	No concerns	Major concerns	No concerns	No concerns	**Very low**
Afatinib+Chemotherapy:Osimertinib+Bevacizumab	0	Low risk	Low risk	No concerns	Major concerns	No concerns	No concerns	**Low**
Afatinib+Chemotherapy:Osimertinib+Chemotherapy	0	Some concerns	Low risk	No concerns	Major concerns	No concerns	No concerns	**Very low**

Afatinib+Chemotherapy:Placebo	0	Low risk	Low risk	No concerns	Major concerns	No concerns	No concerns	**Low**
Chemotherapy:Dacomitinib	0	Some concerns	Low risk	No concerns	Major concerns	No concerns	No concerns	**Very low**
Chemotherapy:Erlotinib+Bevacizumab	0	Some concerns	Low risk	No concerns	Major concerns	No concerns	No concerns	**Very low**
Chemotherapy:Erlotinib+Cabozantinib	0	Some concerns	Low risk	No concerns	Major concerns	No concerns	No concerns	**Very low**
Chemotherapy:Erlotinib+Ramucirumab	0	Low risk	Low risk	No concerns	Major concerns	No concerns	No concerns	**Low**
Chemotherapy:Furmonertinib	0	Low risk	Low risk	No concerns	Major concerns	No concerns	No concerns	**Low**
Chemotherapy:Gefitinib+Anlotinib	0	Low risk	Low risk	No concerns	Major concerns	No concerns	No concerns	**Low**
Chemotherapy:Osimertinib+Bevacizumab	0	Some concerns	Low risk	No concerns	Major concerns	No concerns	No concerns	**Very low**
Chemotherapy:Osimertinib+Chemotherapy	0	Some concerns	Low risk	No concerns	Major concerns	No concerns	No concerns	**Very low**
Chemotherapy:Placebo	0	Low risk	Low risk	No concerns	Some concerns	No concerns	No concerns	**Moderate**
Dacomitinib:Erlotinib+Bevacizumab	0	Low risk	Low risk	No concerns	Major concerns	No concerns	No concerns	**Low**
Dacomitinib:Erlotinib+Cabozantinib	0	Some concerns	Low risk	No concerns	Major concerns	No concerns	No concerns	**Very low**
Dacomitinib:Erlotinib+Chemotherapy	0	Some concerns	Low risk	No concerns	Major concerns	No concerns	No concerns	**Very low**
Dacomitinib:Erlotinib+Ramucirumab	0	Low risk	Low risk	No concerns	No concerns	No concerns	No concerns	**High**
Dacomitinib:Furmonertinib	0	Low risk	Low risk	No concerns	Major concerns	No concerns	No concerns	**Low**
Dacomitinib:Gefitinib+Anlotinib	0	Low risk	Low risk	No concerns	Major concerns	No concerns	No concerns	**Low**
Dacomitinib:Gefitinib+Chemotherapy	0	Some concerns	Low risk	No concerns	Major concerns	No concerns	No concerns	**Very low**
Dacomitinib:Mobocertinib	0	Low risk	Low risk	No concerns	Major concerns	No concerns	No concerns	**Low**
Dacomitinib:Osimertinib	0	Some concerns	Low risk	No concerns	No concerns	Some concerns	No concerns	**Low**
Dacomitinib:Osimertinib+Bevacizumab	0	Some concerns	Low risk	No concerns	Major concerns	No concerns	No concerns	**Very low**
Dacomitinib:Osimertinib+Chemotherapy	0	Some concerns	Low risk	No concerns	Major concerns	No concerns	No concerns	**Very low**
Erlotinib:Furmonertinib	0	Low risk	Low risk	No concerns	Major concerns	No concerns	No concerns	**Low**
Erlotinib:Gefitinib	0	Some concerns	Low risk	No concerns	Major concerns	No concerns	No concerns	**Very low**
Erlotinib:Gefitinib+Anlotinib	0	Low risk	Low risk	No concerns	Major concerns	No concerns	No concerns	**Low**
Erlotinib:Gefitinib+Chemotherapy	0	Some concerns	Low risk	No concerns	Major concerns	No concerns	No concerns	**Very low**
Erlotinib:Mobocertinib	0	Low risk	Low risk	No concerns	No concerns	Some concerns	No concerns	**Moderate**
Erlotinib:Osimertinib	0	Some concerns	Low risk	No concerns	Major concerns	No concerns	No concerns	**Very low**
Erlotinib:Osimertinib+Bevacizumab	0	Some concerns	Low risk	No concerns	Major concerns	No concerns	No concerns	**Very low**
Erlotinib:Osimertinib+Chemotherapy	0	Some concerns	Low risk	No concerns	Major concerns	No concerns	No concerns	**Very low**
Erlotinib+Bevacizumab:Erlotinib+Cabozantinib	0	Some concerns	Low risk	No concerns	Major concerns	No concerns	No concerns	**Very low**
Erlotinib+Bevacizumab:Erlotinib+Chemotherapy	0	Some concerns	Low risk	No concerns	Major concerns	No concerns	No concerns	**Very low**
Erlotinib+Bevacizumab:Erlotinib+Ramucirumab	0	Low risk	Low risk	No concerns	Major concerns	No concerns	No concerns	**Low**
Erlotinib+Bevacizumab:Furmonertinib	0	Low risk	Low risk	No concerns	Major concerns	No concerns	No concerns	**Low**
Erlotinib+Bevacizumab:Gefitinib	0	Low risk	Low risk	No concerns	Major concerns	No concerns	No concerns	**Low**
Erlotinib+Bevacizumab:Gefitinib+Anlotinib	0	Low risk	Low risk	No concerns	Major concerns	No concerns	No concerns	**Low**
Erlotinib+Bevacizumab:Gefitinib+Chemotherapy	0	Some concerns	Low risk	No concerns	Major concerns	No concerns	No concerns	**Very low**
Erlotinib+Bevacizumab:Mobocertinib	0	Low risk	Low risk	No concerns	Major concerns	No concerns	No concerns	**Low**
Erlotinib+Bevacizumab:Osimertinib	0	Some concerns	Low risk	No concerns	Major concerns	No concerns	No concerns	**Very low**
Erlotinib+Bevacizumab:Osimertinib+Bevacizumab	0	Some concerns	Low risk	No concerns	Major concerns	No concerns	No concerns	**Very low**
Erlotinib+Bevacizumab:Osimertinib+Chemotherapy	0	Some concerns	Low risk	No concerns	Major concerns	No concerns	No concerns	**Very low**
Erlotinib+Bevacizumab:Placebo	0	Low risk	Low risk	No concerns	Major concerns	No concerns	No concerns	**Low**
Erlotinib+Cabozantinib:Erlotinib+Chemotherapy	0	Some concerns	Low risk	No concerns	Major concerns	No concerns	No concerns	**Very low**
Erlotinib+Cabozantinib:Erlotinib+Ramucirumab	0	Some concerns	Low risk	No concerns	Major concerns	No concerns	No concerns	**Very low**
Erlotinib+Cabozantinib:Furmonertinib	0	Some concerns	Low risk	No concerns	Major concerns	No concerns	No concerns	**Very low**
Erlotinib+Cabozantinib:Gefitinib	0	Some concerns	Low risk	No concerns	Major concerns	No concerns	No concerns	**Very low**
Erlotinib+Cabozantinib:Gefitinib+Anlotinib	0	Some concerns	Low risk	No concerns	Major concerns	No concerns	No concerns	**Very low**
Erlotinib+Cabozantinib:Gefitinib+Chemotherapy	0	Some concerns	Low risk	No concerns	Major concerns	No concerns	No concerns	**Very low**
Erlotinib+Cabozantinib:Mobocertinib	0	Some concerns	Low risk	No concerns	Major concerns	No concerns	No concerns	**Very low**
Erlotinib+Cabozantinib:Osimertinib	0	Some concerns	Low risk	No concerns	Major concerns	No concerns	No concerns	**Very low**
Erlotinib+Cabozantinib:Osimertinib+Bevacizumab	0	Some concerns	Low risk	No concerns	Major concerns	No concerns	No concerns	**Very low**
Erlotinib+Cabozantinib:Osimertinib+Chemotherapy	0	Some concerns	Low risk	No concerns	Major concerns	No concerns	No concerns	**Very low**
Erlotinib+Cabozantinib:Placebo	0	Low risk	Low risk	No concerns	Major concerns	No concerns	No concerns	**Low**
Erlotinib+Chemotherapy:Erlotinib+Ramucirumab	0	Some concerns	Low risk	No concerns	Major concerns	No concerns	No concerns	**Very low**
Erlotinib+Chemotherapy:Furmonertinib	0	Low risk	Low risk	No concerns	Major concerns	No concerns	No concerns	**Low**
Erlotinib+Chemotherapy:Gefitinib	0	Some concerns	Low risk	No concerns	Major concerns	No concerns	No concerns	**Very low**
Erlotinib+Chemotherapy:Gefitinib+Anlotinib	0	Low risk	Low risk	No concerns	Major concerns	No concerns	No concerns	**Low**
Erlotinib+Chemotherapy:Gefitinib+Chemotherapy	0	Some concerns	Low risk	No concerns	Major concerns	No concerns	No concerns	**Very low**
Erlotinib+Chemotherapy:Mobocertinib	0	Some concerns	Low risk	No concerns	Major concerns	No concerns	No concerns	**Very low**
Erlotinib+Chemotherapy:Osimertinib	0	Some concerns	Low risk	No concerns	Major concerns	No concerns	No concerns	**Very low**
Erlotinib+Chemotherapy:Osimertinib+Bevacizumab	0	Some concerns	Low risk	No concerns	Major concerns	No concerns	No concerns	**Very low**
Erlotinib+Chemotherapy:Osimertinib+Chemotherapy	0	Some concerns	Low risk	No concerns	Major concerns	No concerns	No concerns	**Very low**
Erlotinib+Chemotherapy:Placebo	0	Low risk	Low risk	No concerns	Major concerns	No concerns	No concerns	**Low**
Erlotinib+Ramucirumab:Furmonertinib	0	Low risk	Low risk	No concerns	Major concerns	No concerns	No concerns	**Low**
Erlotinib+Ramucirumab:Gefitinib	0	Low risk	Low risk	No concerns	Major concerns	No concerns	No concerns	**Low**
Erlotinib+Ramucirumab:Gefitinib+Anlotinib	0	Low risk	Low risk	No concerns	Major concerns	No concerns	No concerns	**Low**
Erlotinib+Ramucirumab:Gefitinib+Chemotherapy	0	Low risk	Low risk	No concerns	Major concerns	No concerns	No concerns	**Low**
Erlotinib+Ramucirumab:Mobocertinib	0	Low risk	Low risk	No concerns	Some concerns	Some concerns	No concerns	**Moderate**
Erlotinib+Ramucirumab:Osimertinib	0	Some concerns	Low risk	No concerns	Major concerns	No concerns	No concerns	**Very low**
Erlotinib+Ramucirumab:Osimertinib+Bevacizumab	0	Some concerns	Low risk	No concerns	Major concerns	No concerns	No concerns	**Very low**
Erlotinib+Ramucirumab:Osimertinib+Chemotherapy	0	Some concerns	Low risk	No concerns	Major concerns	No concerns	No concerns	**Very low**
Erlotinib+Ramucirumab:Placebo	0	Low risk	Low risk	No concerns	Major concerns	No concerns	No concerns	**Low**
Furmonertinib:Gefitinib+Anlotinib	0	Low risk	Low risk	No concerns	Major concerns	No concerns	No concerns	**Low**
Furmonertinib:Gefitinib+Chemotherapy	0	Low risk	Low risk	No concerns	Major concerns	No concerns	No concerns	**Low**
Furmonertinib:Mobocertinib	0	Low risk	Low risk	No concerns	No concerns	Some concerns	No concerns	**Moderate**
Furmonertinib:Osimertinib	0	Some concerns	Low risk	No concerns	Major concerns	No concerns	No concerns	**Very low**
Furmonertinib:Osimertinib+Bevacizumab	0	Some concerns	Low risk	No concerns	Major concerns	No concerns	No concerns	**Very low**
Furmonertinib:Osimertinib+Chemotherapy	0	Some concerns	Low risk	No concerns	Major concerns	No concerns	No concerns	**Very low**
Furmonertinib:Placebo	0	Low risk	Low risk	No concerns	Major concerns	No concerns	No concerns	**Low**
Gefitinib:Mobocertinib	0	Low risk	Low risk	No concerns	No concerns	No concerns	No concerns	**High**
Gefitinib:Osimertinib	0	Some concerns	Low risk	No concerns	Major concerns	No concerns	No concerns	**Very low**
Gefitinib:Osimertinib+Bevacizumab	0	Some concerns	Low risk	No concerns	Major concerns	No concerns	No concerns	**Very low**
Gefitinib:Osimertinib+Chemotherapy	0	Some concerns	Low risk	No concerns	Major concerns	No concerns	No concerns	**Very low**
Gefitinib+Anlotinib:Gefitinib+Chemotherapy	0	Low risk	Low risk	No concerns	Major concerns	No concerns	No concerns	**Low**
Gefitinib+Anlotinib:Mobocertinib	0	Low risk	Low risk	No concerns	Major concerns	No concerns	No concerns	**Low**
Gefitinib+Anlotinib:Osimertinib	0	Some concerns	Low risk	No concerns	Major concerns	No concerns	No concerns	**Very low**
Gefitinib+Anlotinib:Osimertinib+Bevacizumab	0	Some concerns	Low risk	No concerns	Major concerns	No concerns	No concerns	**Very low**
Gefitinib+Anlotinib:Osimertinib+Chemotherapy	0	Some concerns	Low risk	No concerns	Major concerns	No concerns	No concerns	**Very low**
Gefitinib+Anlotinib:Placebo	0	Low risk	Low risk	No concerns	Major concerns	No concerns	No concerns	**Low**
Gefitinib+Chemotherapy:Mobocertinib	0	Low risk	Low risk	No concerns	Major concerns	No concerns	No concerns	**Low**
Gefitinib+Chemotherapy:Osimertinib	0	Some concerns	Low risk	No concerns	Major concerns	No concerns	No concerns	**Very low**
Gefitinib+Chemotherapy:Osimertinib+Bevacizumab	0	Some concerns	Low risk	No concerns	Major concerns	No concerns	No concerns	**Very low**
Gefitinib+Chemotherapy:Osimertinib+Chemotherapy	0	Some concerns	Low risk	No concerns	Major concerns	No concerns	No concerns	**Very low**
Gefitinib+Chemotherapy:Placebo	0	Low risk	Low risk	No concerns	No concerns	No concerns	No concerns	**High**
Mobocertinib:Osimertinib	0	Some concerns	Low risk	No concerns	No concerns	No concerns	No concerns	**Moderate**
Mobocertinib:Osimertinib+Bevacizumab	0	Low risk	Low risk	No concerns	Some concerns	Some concerns	No concerns	**Moderate**

Mobocertinib:Osimertinib+Chemotherapy	0	Some concerns	Low risk	No concerns	Some concerns	Some concerns	No concerns	**Low**
Mobocertinib:Placebo	0	Low risk	Low risk	No concerns	No concerns	No concerns	No concerns	**High**
Osimertinib:Placebo	0	Some concerns	Low risk	No concerns	Major concerns	No concerns	No concerns	**Very low**
Osimertinib+Bevacizumab:Osimertinib+Chemotherapy	0	Some concerns	Low risk	No concerns	Major concerns	No concerns	No concerns	**Very low**
Osimertinib+Bevacizumab:Placebo	0	Some concerns	Low risk	No concerns	Major concerns	No concerns	No concerns	**Very low**
Osimertinib+Chemotherapy:Placebo	0	Some concerns	Low risk	No concerns	Major concerns	No concerns	No concerns	**Very low**
