# Supplementary material for: Oral mucositis risk with EGFR tyrosine kinase inhibitor monotherapies and combination regimens in non-small cell lung cancer: a systematic review and network meta-analysis
Source: Front Immunol. 2026 Sep 18;17:1957825. doi: 10.3389/fimmu.2026.1957825 (PMC13631102; doi:10.3389/fimmu.2026.1957825)
Supplement: Supplementary file 3 [file DataSheet3.pdf]

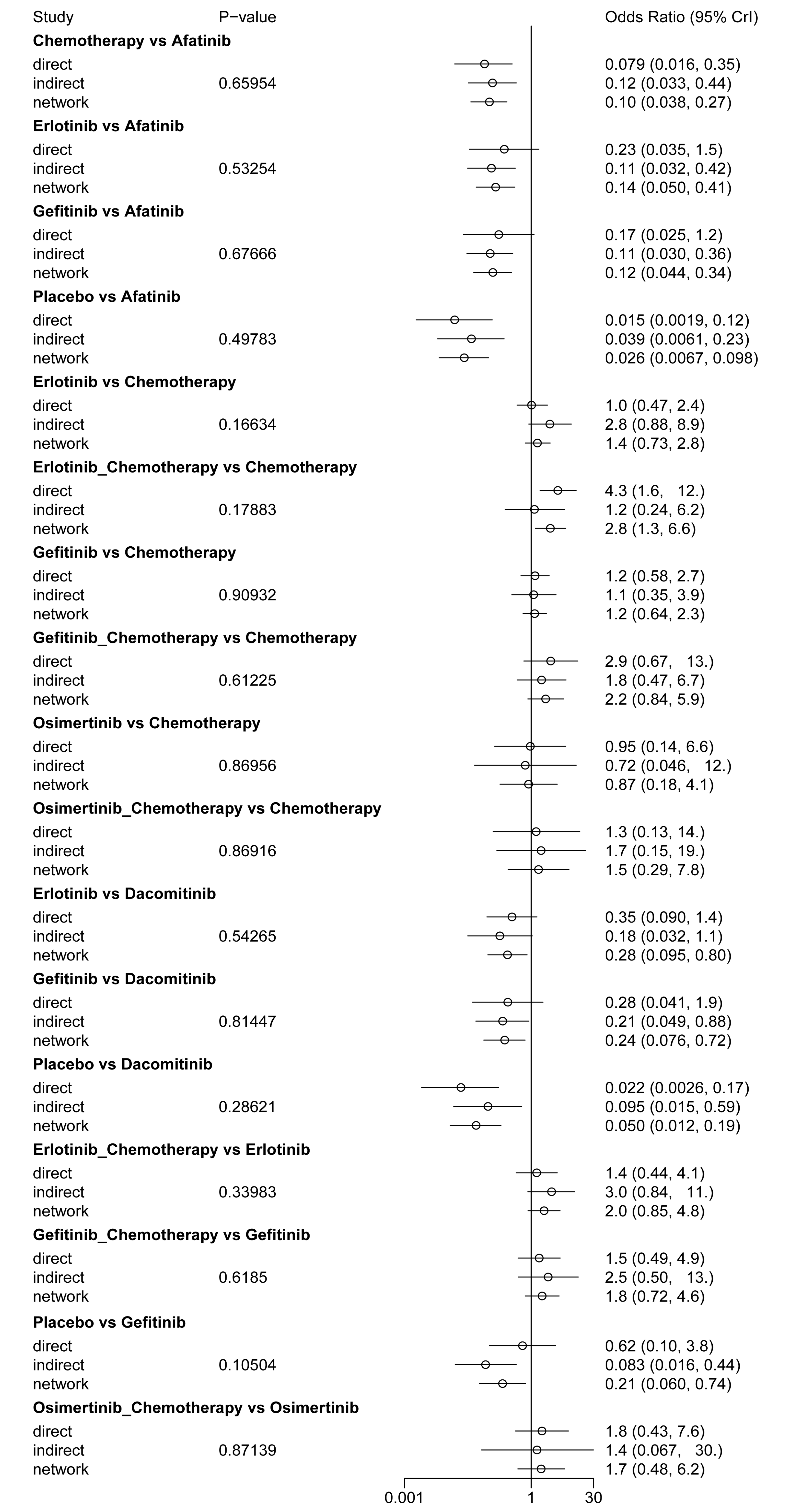

Supplementary Figure3.1 Node-splitting analyses of local inconsistency for any-grade.

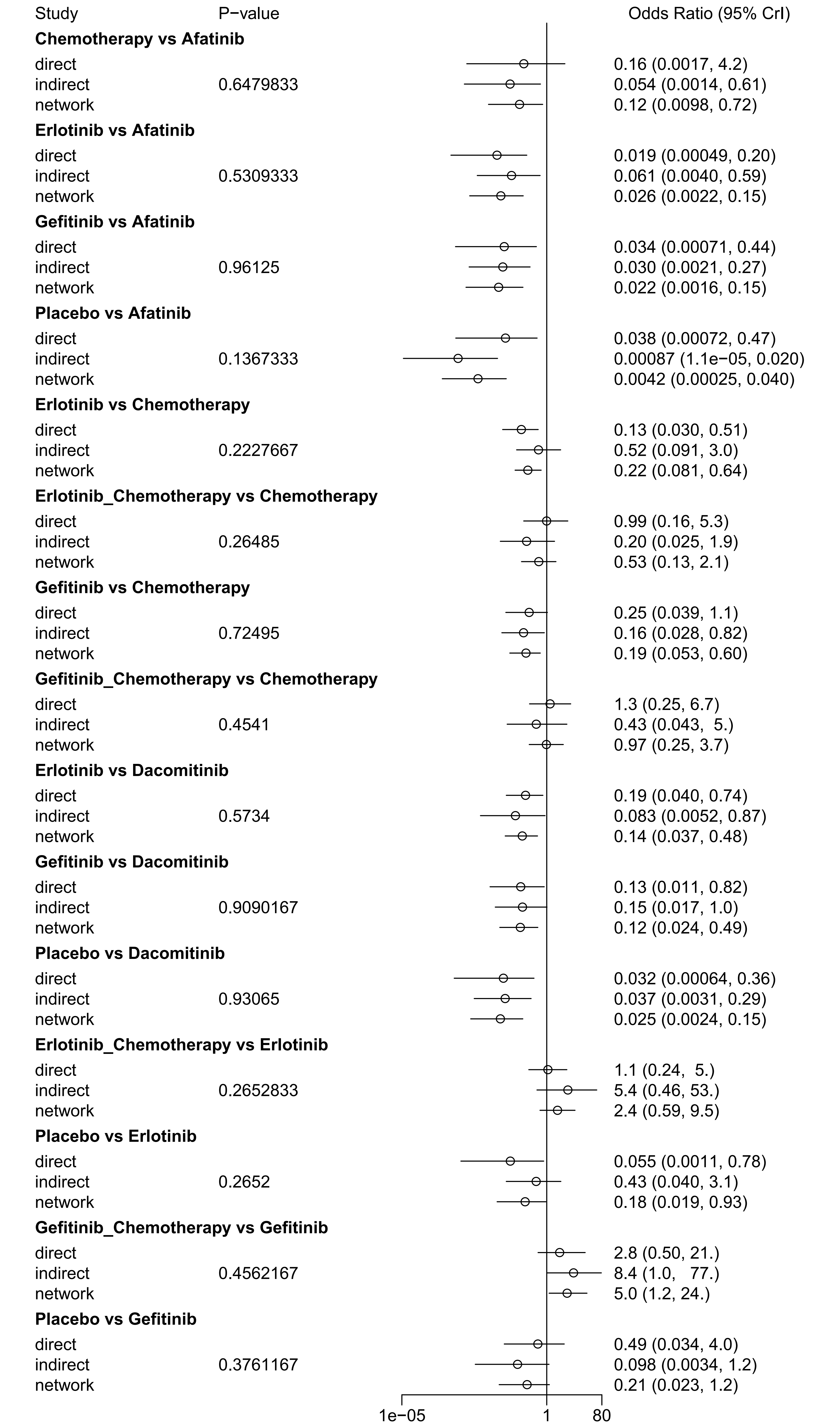

Supplementary Figure3.2 Node-splitting analyses of local inconsistency for grade ≥3 oral mucositi
