# Supplementary material for: Oral mucositis risk with EGFR tyrosine kinase inhibitor monotherapies and combination regimens in non-small cell lung cancer: a systematic review and network meta-analysis
Source: Front Immunol. 2026 Sep 18;17:1957825. doi: 10.3389/fimmu.2026.1957825 (PMC13631102; doi:10.3389/fimmu.2026.1957825)
Supplement: Supplementary file 4 [file DataSheet4.pdf]

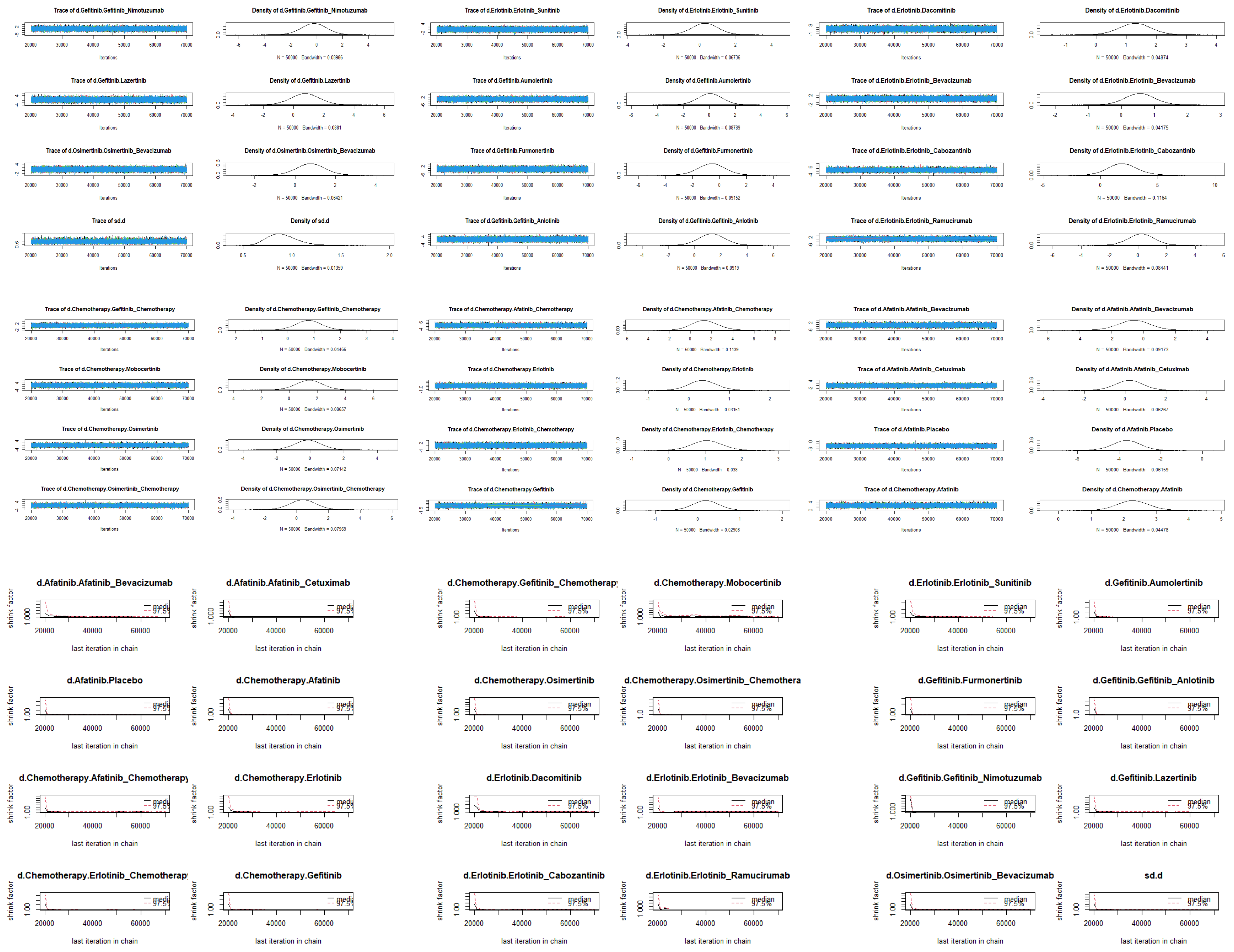

Supplementary Figure4.1 Trace and posterior density plots for the Bayesian network meta-analysis of any-grade oral mucositis.

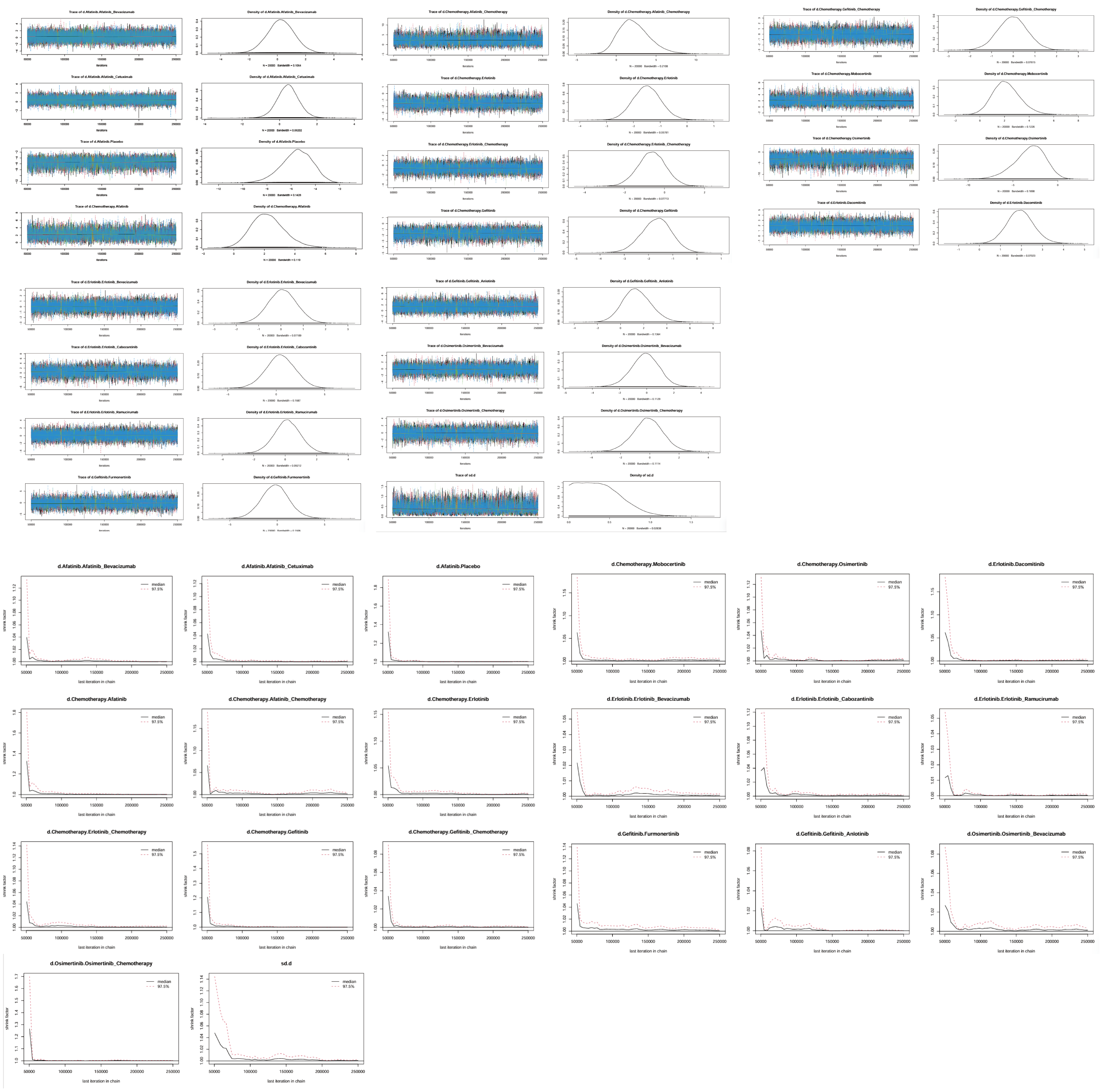

Supplementary Figure4.2 Trace and posterior density plots for the Bayesian network meta-analysis of grade ≥3 oral mucositis.
