# Supplementary material for: Oral mucositis risk with EGFR tyrosine kinase inhibitor monotherapies and combination regimens in non-small cell lung cancer: a systematic review and network meta-analysis
Source: Front Immunol. 2026 Sep 18;17:1957825. doi: 10.3389/fimmu.2026.1957825 (PMC13631102; doi:10.3389/fimmu.2026.1957825)
Supplement: Supplementary file 5 [file DataSheet5.pdf]

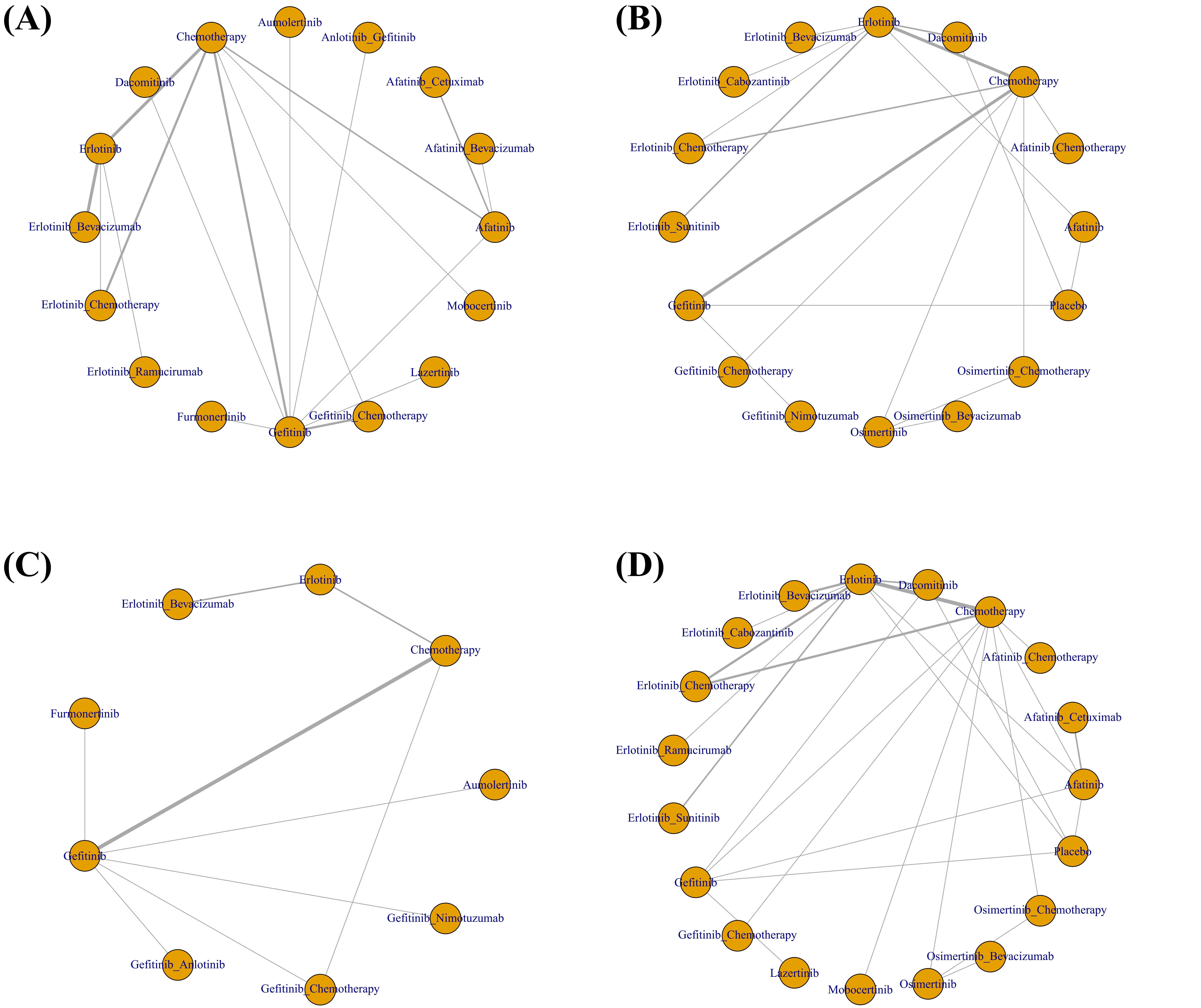

Supplementary Figure5. Network plots of subgroup analyses for any-grade oral mucositis: (A) first-line treatment; (B) second-line or later treatment (≥2 lines); (C) exclusively Asian populations; and (D) non-exclusively Asian populations.
