# Supplementary material for: Oral mucositis risk with EGFR tyrosine kinase inhibitor monotherapies and combination regimens in non-small cell lung cancer: a systematic review and network meta-analysis
Source: Front Immunol. 2026 Sep 18;17:1957825. doi: 10.3389/fimmu.2026.1957825 (PMC13631102; doi:10.3389/fimmu.2026.1957825)
Supplement: Supplementary file 6 [file DataSheet6.pdf]

(A)

Afatinib_Cetuximab	0.83 (0.47–1.49)	0.57 (0.18–1.89)	0.56 (0.20–1.50)	0.57 (0.17–2.00)	0.47 (0.15–1.42)	0.38 (0.11–1.35)	0.33 (0.11–1.00)	0.33 (0.11–0.95)	0.31 (0.11–0.95)	0.30 (0.11–0.82)	0.25 (0.10–0.59)	0.16 (0.05–0.49)	0.16 (0.07–0.33)	0.09 (0.03–0.29)	0.08 (0.04–0.17)
1.20 (0.67–2.11)	Afatinib	0.68 (0.25–1.97)	0.67 (0.29–1.51)	0.68 (0.23–2.11)	0.56 (0.21–1.44)	0.46 (0.16–1.42)	0.40 (0.16–1.03)	0.40 (0.16–0.96)	0.37 (0.15–0.98)	0.36 (0.16–0.82)	0.30 (0.15–0.58)	0.20 (0.08–0.50)	0.19 (0.11–0.31)	0.10 (0.03–0.30)	0.10 (0.05–0.16)
1.76 (0.53–5.56)	1.48 (0.51–4.05)	Erlotinib_Bevacizumab	0.99 (0.30–2.97)	1.00 (0.26–3.93)	0.82 (0.20–3.40)	0.68 (0.32–1.46)	0.59 (0.16–2.00)	0.59 (0.18–1.76)	0.55 (0.34–0.89)	0.53 (0.21–1.30)	0.45 (0.15–1.21)	0.29 (0.08–0.96)	0.27 (0.10–0.70)	0.15 (0.04–0.57)	0.14 (0.05–0.33)
1.80 (0.67–4.91)	1.50 (0.66–3.44)	1.02 (0.34–3.34)	Dacomitinib	1.02 (0.33–3.37)	0.84 (0.24–2.94)	0.69 (0.21–2.38)	0.60 (0.22–1.67)	0.59 (0.21–1.65)	0.56 (0.21–1.68)	0.53 (0.21–1.44)	0.45 (0.21–0.95)	0.29 (0.11–0.80)	0.28 (0.15–0.52)	0.16 (0.05–0.47)	0.14 (0.07–0.30)
1.74 (0.50–6.04)	1.46 (0.47–4.28)	1.00 (0.25–3.85)	0.98 (0.30–3.01)	Anlotinib_Gefitinib	0.82 (0.19–3.38)	0.67 (0.16–2.73)	0.59 (0.16–1.98)	0.59 (0.16–1.96)	0.55 (0.15–1.98)	0.52 (0.15–1.73)	0.45 (0.15–1.21)	0.29 (0.08–0.98)	0.27 (0.10–0.69)	0.15 (0.04–0.56)	0.14 (0.05–0.38)
2.14 (0.70–6.63)	1.79 (0.70–4.72)	1.23 (0.29–5.08)	1.20 (0.34–4.19)	1.23 (0.30–5.34)	Afatinib_Bevacizumab	0.83 (0.20–3.59)	0.72 (0.19–2.76)	0.71 (0.19–2.60)	0.68 (0.18–2.60)	0.64 (0.18–2.33)	0.54 (0.17–1.70)	0.35 (0.09–1.35)	0.33 (0.11–0.99)	0.19 (0.04–0.78)	0.17 (0.06–0.50)
2.60 (0.74–8.72)	2.17 (0.71–6.29)	1.48 (0.68–3.15)	1.46 (0.42–4.69)	1.49 (0.37–6.07)	1.21 (0.28–5.02)	Erlotinib_Ramucirumab	0.87 (0.23–3.11)	0.86 (0.26–2.74)	0.82 (0.45–1.49)	0.78 (0.29–2.06)	0.66 (0.21–1.87)	0.43 (0.11–1.50)	0.41 (0.14–1.09)	0.23 (0.05–0.88)	0.21 (0.08–0.52)
3.00 (1.00–9.00)	2.50 (0.97–6.40)	1.70 (0.50–6.07)	1.67 (0.60–4.55)	1.71 (0.50–6.11)	1.39 (0.36–5.31)	1.15 (0.32–4.26)	Lazertinib	1.00 (0.32–2.99)	0.94 (0.31–3.04)	0.90 (0.30–2.60)	0.76 (0.30–1.81)	0.49 (0.16–1.48)	0.46 (0.21–1.01)	0.26 (0.08–0.86)	0.24 (0.10–0.56)
3.02 (1.06–8.79)	2.52 (1.05–6.19)	1.71 (0.57–5.46)	1.68 (0.61–4.69)	1.70 (0.51–6.25)	1.40 (0.39–5.25)	1.16 (0.37–3.90)	1.00 (0.33–3.15)	Mobocertinib	0.95 (0.35–2.73)	0.90 (0.36–2.32)	0.76 (0.31–1.81)	0.49 (0.16–1.53)	0.47 (0.21–1.04)	0.26 (0.07–0.88)	0.24 (0.12–0.48)
3.19 (1.05–9.13)	2.67 (1.02–6.51)	1.80 (1.12–2.90)	1.78 (0.60–4.83)	1.81 (0.51–6.49)	1.47 (0.38–5.57)	1.22 (0.67–2.24)	1.06 (0.33–3.24)	1.05 (0.37–2.84)	Erlotinib	0.95 (0.42–2.05)	0.81 (0.30–1.93)	0.52 (0.16–1.57)	0.50 (0.20–1.11)	0.28 (0.07–0.95)	0.25 (0.11–0.52)
3.35 (1.22–9.09)	2.80 (1.22–6.33)	1.90 (0.77–4.83)	1.87 (0.69–4.84)	1.92 (0.58–6.48)	1.57 (0.43–5.46)	1.29 (0.48–3.49)	1.11 (0.38–3.39)	1.11 (0.43–2.81)	1.05 (0.49–2.37)	Erlotinib_Chemotherapy	0.85 (0.36–1.87)	0.55 (0.19–1.57)	0.52 (0.24–1.06)	0.29 (0.08–0.95)	0.27 (0.14–0.49)
3.96 (1.69–9.70)	3.31 (1.74–6.56)	2.24 (0.83–6.75)	2.21 (1.06–4.83)	2.25 (0.83–6.80)	1.85 (0.59–5.98)	1.52 (0.53–4.83)	1.32 (0.55–3.38)	1.32 (0.55–3.20)	1.24 (0.52–3.39)	1.18 (0.54–2.78)	Gefitinib_Chemotherapy	0.65 (0.27–1.61)	0.61 (0.40–0.95)	0.35 (0.12–0.99)	0.31 (0.19–0.54)
6.08 (2.04–18.55)	5.11 (1.98–13.22)	3.46 (1.04–12.45)	3.41 (1.25–9.26)	3.48 (1.02–12.28)	2.86 (0.74–10.86)	2.34 (0.67–8.81)	2.04 (0.67–6.15)	2.03 (0.65–6.21)	1.91 (0.64–6.37)	1.82 (0.64–5.40)	1.54 (0.62–3.68)	Aumolertinib	0.95 (0.43–2.06)	0.53 (0.15–1.78)	0.48 (0.20–1.15)
6.44 (2.99–14.15)	5.39 (3.22–9.24)	3.65 (1.42–10.07)	3.59 (1.92–6.79)	3.67 (1.45–10.05)	3.01 (1.01–8.97)	2.47 (0.91–7.26)	2.15 (0.99–4.83)	2.14 (0.96–4.75)	2.01 (0.90–4.99)	1.92 (0.94–4.14)	1.63 (1.05–2.48)	1.06 (0.49–2.31)	Gefitinib	0.56 (0.21–1.42)	0.51 (0.35–0.74)
11.49 (3.44–39.77)	9.64 (3.36–29.20)	6.55 (1.74–26.10)	6.40 (2.11–20.80)	6.58 (1.78–25.36)	5.37 (1.28–23.24)	4.42 (1.14–18.72)	3.85 (1.16–13.30)	3.80 (1.14–13.38)	3.63 (1.05–13.55)	3.40 (1.05–11.86)	2.89 (1.01–8.27)	1.88 (0.56–6.59)	1.77 (0.70–4.76)	Furmonertinib	0.91 (0.34–2.58)
12.57 (5.82–28.18)	10.49 (6.29–18.45)	7.12 (3.03–18.32)	7.03 (3.38–14.88)	7.16 (2.65–21.18)	5.87 (1.98–17.78)	4.82 (1.92–13.12)	4.21 (1.78–10.34)	4.18 (2.08–8.50)	3.95 (1.92–8.95)	3.76 (2.05–7.22)	3.18 (1.86–5.31)	2.06 (0.87–4.97)	1.95 (1.34–2.87)	1.10 (0.39–2.95)	Chemotherapy

(B)

Afatinib	1.11 (0.01–102.38)	0.83 (0.03–25.15)	0.92 (0.01–67.44)	0.94 (0.01–78.33)	0.57 (0.03–10.47)	0.57 (0.00–82.71)	0.46 (0.01–22.49)	0.28 (0.02–4.58)	0.23 (0.01–10.47)	0.21 (0.00–11.43)	0.18 (0.01–5.50)	0.13 (0.00–9.52)	0.16 (0.01–2.95)	0.14 (0.01–1.67)	0.03 (0.00–0.35)
0.90 (0.01–76.11)	Afatinib_Chemotherapy	0.75 (0.01–46.54)	0.83 (0.01–95.52)	0.86 (0.00–162.94)	0.51 (0.01–33.92)	0.51 (0.00–112.25)	0.42 (0.00–34.67)	0.26 (0.01–8.13)	0.21 (0.00–16.19)	0.19 (0.00–25.97)	0.16 (0.00–14.10)	0.12 (0.00–16.11)	0.14 (0.00–6.13)	0.12 (0.00–5.45)	0.02 (0.00–1.71)
1.20 (0.04–32.86)	1.33 (0.02–84.26)	Erlotinib_Chemotherapy	1.11 (0.02–52.78)	1.13 (0.02–83.61)	0.68 (0.03–13.36)	0.70 (0.01–70.44)	0.56 (0.02–17.45)	0.35 (0.04–2.93)	0.28 (0.01–8.06)	0.26 (0.00–12.08)	0.22 (0.01–5.80)	0.16 (0.00–8.89)	0.19 (0.01–2.51)	0.16 (0.01–1.70)	0.03 (0.00–0.71)
1.09 (0.01–80.58)	1.21 (0.01–154.94)	0.90 (0.02–49.05)	Gefitinib_Chemotherapy	1.03 (0.01–176.66)	0.62 (0.01–36.49)	0.62 (0.00–118.55)	0.50 (0.01–35.58)	0.31 (0.01–8.12)	0.25 (0.00–16.41)	0.23 (0.00–27.37)	0.20 (0.00–14.69)	0.14 (0.00–16.80)	0.17 (0.00–6.26)	0.15 (0.00–5.66)	0.03 (0.00–1.75)
1.07 (0.01–78.46)	1.17 (0.01–221.12)	0.88 (0.01–62.71)	0.97 (0.01–152.45)	Erlotinib_Cabozantinib	0.60 (0.01–33.22)	0.60 (0.00–168.02)	0.49 (0.00–53.32)	0.30 (0.01–13.85)	0.25 (0.00–24.62)	0.22 (0.00–24.23)	0.19 (0.00–12.71)	0.14 (0.00–23.17)	0.17 (0.00–9.60)	0.15 (0.00–4.75)	0.03 (0.00–1.95)
1.77 (0.10–33.08)	1.96 (0.03–144.47)	1.47 (0.07–31.86)	1.63 (0.03–93.89)	1.66 (0.03–105.43)	Dacomitinib	1.01 (0.01–119.72)	0.82 (0.02–30.12)	0.51 (0.05–5.46)	0.41 (0.01–13.94)	0.37 (0.01–14.76)	0.32 (0.02–6.70)	0.23 (0.00–13.61)	0.28 (0.02–3.77)	0.24 (0.03–1.76)	0.05 (0.00–0.55)
1.74 (0.01–251.89)	1.94 (0.01–452.06)	1.44 (0.01–168.29)	1.60 (0.01–307.89)	1.66 (0.01–472.67)	0.99 (0.01–117.39)	Osimertinib_Bevacizumab	0.81 (0.01–52.19)	0.50 (0.01–30.76)	0.41 (0.02–9.84)	0.37 (0.00–82.51)	0.32 (0.00–46.65)	0.23 (0.00–51.79)	0.28 (0.00–22.43)	0.24 (0.00–19.48)	0.05 (0.00–5.81)
2.16 (0.04–105.31)	2.40 (0.03–213.09)	1.78 (0.06–62.98)	1.99 (0.03–138.28)	2.04 (0.02–248.79)	1.22 (0.03–45.79)	1.24 (0.02–79.87)	Osimertinib_Chemotherapy	0.62 (0.04–9.26)	0.50 (0.03–7.29)	0.46 (0.01–38.09)	0.39 (0.01–19.31)	0.28 (0.00–24.29)	0.34 (0.02–7.68)	0.29 (0.01–6.70)	0.06 (0.00–2.27)
3.51 (0.22–56.72)	3.86 (0.12–139.98)	2.89 (0.34–27.64)	3.20 (0.12–85.07)	3.31 (0.07–177.12)	1.98 (0.18–21.84)	2.00 (0.03–125.30)	1.62 (0.11–25.04)	Chemotherapy	0.81 (0.06–11.13)	0.74 (0.02–24.23)	0.64 (0.04–10.47)	0.46 (0.01–15.30)	0.56 (0.12–2.53)	0.48 (0.10–2.30)	0.10 (0.01–1.09)
4.32 (0.10–191.48)	4.77 (0.06–406.85)	3.56 (0.12–117.42)	3.95 (0.06–258.94)	4.06 (0.04–451.83)	2.43 (0.07–84.49)	2.45 (0.10–60.49)	2.00 (0.14–29.64)	1.23 (0.09–16.94)	Osimertinib	0.91 (0.01–71.66)	0.79 (0.02–35.86)	0.56 (0.01–45.20)	0.68 (0.03–14.06)	0.59 (0.03–12.42)	0.12 (0.00–4.24)
4.75 (0.09–258.91)	5.25 (0.04–774.45)	3.91 (0.08–209.84)	4.35 (0.04–513.10)	4.48 (0.04–536.11)	2.69 (0.07–109.40)	2.72 (0.01–594.50)	2.19 (0.03–180.29)	1.35 (0.04–43.93)	1.10 (0.01–84.68)	Erlotinib_Bevacizumab	0.86 (0.02–42.85)	0.62 (0.00–80.62)	0.75 (0.02–30.94)	0.64 (0.03–14.63)	0.13 (0.00–6.40)
5.45 (0.18–164.28)	6.08 (0.07–563.20)	4.53 (0.17–130.10)	5.01 (0.07–368.30)	5.15 (0.08–373.64)	3.08 (0.15–63.99)	3.11 (0.02–451.70)	2.54 (0.05–125.81)	1.56 (0.10–24.99)	1.27 (0.03–56.36)	1.16 (0.02–54.03)	Erlotinib_Sunitinib	0.72 (0.01–57.84)	0.87 (0.04–18.57)	0.74 (0.07–7.38)	0.15 (0.01–4.00)
7.62 (0.10–565.40)	8.46 (0.06–1230.24)	6.33 (0.11–421.05)	7.01 (0.06–854.52)	7.25 (0.04–1340.40)	4.30 (0.07–265.49)	4.39 (0.02–988.52)	3.54 (0.04–303.06)	2.18 (0.07–73.14)	1.77 (0.02–141.29)	1.61 (0.01–214.13)	1.40 (0.02–112.82)	Gefitinib_Nimotuzumab	1.21 (0.05–28.61)	1.04 (0.02–44.93)	0.21 (0.00–11.06)
6.29 (0.34–117.18)	6.95 (0.16–337.78)	5.21 (0.40–76.65)	5.75 (0.16–211.52)	5.94 (0.10–393.69)	3.55 (0.26–49.24)	3.59 (0.04–296.22)	2.91 (0.13–66.46)	1.80 (0.40–8.19)	1.46 (0.07–30.00)	1.33 (0.03–55.59)	1.15 (0.05–25.06)	0.83 (0.03–19.60)	Gefitinib	0.85 (0.11–6.59)	0.17 (0.02–1.92)
7.36 (0.60–89.85)	8.14 (0.18–403.38)	6.09 (0.59–69.57)	6.71 (0.18–257.76)	6.87 (0.21–263.43)	4.16 (0.57–30.30)	4.20 (0.05–351.55)	3.41 (0.15–77.67)	2.10 (0.43–9.95)	1.70 (0.08–35.46)	1.55 (0.07–34.48)	1.34 (0.14–13.53)	0.96 (0.02–40.87)	1.17 (0.15–8.99)	Erlotinib	0.20 (0.02–2.11)
35.96 (2.89–465.78)	40.09 (0.59–3027.03)	29.76 (1.40–747.10)	32.88 (0.57–2010.17)	33.88 (0.51–2576.56)	20.31 (1.80–236.53)	20.65 (0.17–2539.65)	16.63 (0.44–660.01)	10.22 (0.92–118.82)	8.37 (0.24–303.10)	7.56 (0.16–381.26)	6.57 (0.25–182.86)	4.69 (0.09–250.75)	5.72 (0.52–64.60)	4.88 (0.47–52.50)	Placebo

(C)

Erlotinib_Bevacizumab	0.77 (0.06–9.43)	0.44 (0.05–3.88)	0.46 (0.15–1.40)	0.22 (0.02–2.65)	0.21 (0.03–1.44)	0.17 (0.01–2.06)	0.16 (0.02–1.01)	0.12 (0.01–1.44)
1.30 (0.11–17.97)	Gefitinib_Anlotinib	0.58 (0.08–4.34)	0.59 (0.06–6.27)	0.29 (0.03–2.76)	0.27 (0.05–1.39)	0.22 (0.02–2.21)	0.21 (0.04–1.30)	0.15 (0.01–1.50)
2.26 (0.26–21.28)	1.72 (0.23–12.86)	Gefitinib_Chemotherapy	1.02 (0.16–7.21)	0.50 (0.07–3.48)	0.47 (0.14–1.51)	0.39 (0.05–2.79)	0.36 (0.11–1.23)	0.26 (0.03–1.93)
2.19 (0.71–6.83)	1.70 (0.16–16.37)	0.98 (0.14–6.21)	Erlotinib	0.48 (0.05–4.41)	0.46 (0.08–2.23)	0.38 (0.04–3.49)	0.36 (0.08–1.50)	0.26 (0.02–2.44)
4.54 (0.38–58.97)	3.49 (0.36–33.65)	2.01 (0.29–14.18)	2.06 (0.23–20.66)	Aumolertinib	0.95 (0.20–4.49)	0.77 (0.08–7.32)	0.73 (0.14–4.25)	0.53 (0.05–5.00)
4.77 (0.69–37.28)	3.67 (0.72–19.19)	2.12 (0.66–6.98)	2.16 (0.45–12.05)	1.06 (0.22–5.07)	Gefitinib	0.82 (0.17–4.07)	0.77 (0.39–1.64)	0.56 (0.11–2.80)
5.84 (0.48–78.47)	4.50 (0.45–44.91)	2.59 (0.36–19.23)	2.65 (0.29–27.30)	1.29 (0.14–12.14)	1.22 (0.25–6.00)	Gefitinib_Nimotuzumab	0.94 (0.17–5.64)	0.68 (0.07–6.68)
6.15 (0.99–41.67)	4.74 (0.77–27.33)	2.75 (0.81–8.77)	2.78 (0.66–12.94)	1.38 (0.24–7.40)	1.30 (0.61–2.54)	1.06 (0.18–5.92)	Chemotherapy	0.72 (0.11–4.05)
8.54 (0.69–119.06)	6.53 (0.67–67.43)	3.80 (0.52–28.89)	3.88 (0.41–41.97)	1.90 (0.20–18.54)	1.79 (0.36–9.26)	1.47 (0.15–14.67)	1.38 (0.25–8.70)	Furmonertinib

(D)

Afatinib_Cetuximab	0.82 (0.15–4.64)	1.09 (0.03–49.67)	0.77 (0.03–21.27)	0.72 (0.02–30.71)	0.59 (0.02–18.68)	0.46 (0.04–5.39)	0.39 (0.03–4.85)	0.42 (0.01–21.82)	0.25 (0.01–5.62)	0.23 (0.02–3.59)	0.21 (0.01–4.03)	0.19 (0.01–5.49)	0.19 (0.01–5.37)	0.17 (0.01–3.60)	0.18 (0.02–1.81)	0.16 (0.02–1.58)	0.09 (0.01–0.91)	0.02 (0.00–0.18)
1.22 (0.22–6.87)	Afatinib	1.33 (0.06–40.06)	0.95 (0.06–16.01)	0.88 (0.04–24.78)	0.72 (0.04–14.74)	0.56 (0.10–3.27)	0.47 (0.08–2.90)	0.51 (0.02–17.71)	0.30 (0.02–4.09)	0.28 (0.04–2.39)	0.26 (0.03–2.75)	0.23 (0.01–4.13)	0.23 (0.01–3.97)	0.21 (0.02–2.57)	0.23 (0.05–1.00)	0.19 (0.05–0.87)	0.11 (0.02–0.51)	0.02 (0.00–0.11)
0.92 (0.02–33.66)	0.75 (0.02–17.89)	Erlotinib_Cabozantinib	0.70 (0.01–32.77)	0.66 (0.01–45.95)	0.54 (0.01–28.36)	0.42 (0.01–10.13)	0.36 (0.01–7.86)	0.38 (0.00–30.93)	0.23 (0.00–9.01)	0.21 (0.01–5.32)	0.19 (0.01–5.72)	0.17 (0.00–9.68)	0.18 (0.00–7.27)	0.16 (0.00–5.77)	0.17 (0.01–3.38)	0.14 (0.01–2.49)	0.08 (0.00–2.02)	0.02 (0.00–0.41)
1.30 (0.05–36.13)	1.06 (0.06–18.11)	1.42 (0.03–81.31)	Mobocertinib	0.94 (0.02–43.60)	0.77 (0.02–26.64)	0.60 (0.03–10.85)	0.50 (0.03–7.78)	0.54 (0.01–30.59)	0.32 (0.01–7.95)	0.30 (0.02–6.41)	0.27 (0.01–6.87)	0.25 (0.01–10.62)	0.25 (0.01–8.84)	0.22 (0.01–5.22)	0.24 (0.02–2.71)	0.20 (0.02–2.89)	0.12 (0.01–2.03)	0.02 (0.00–0.43)
1.39 (0.03–51.63)	1.13 (0.04–26.70)	1.51 (0.02–107.87)	1.07 (0.02–43.01)	Afatinib_Chemotherapy	0.82 (0.02–36.74)	0.64 (0.02–16.23)	0.54 (0.02–11.69)	0.57 (0.01–41.32)	0.34 (0.01–11.70)	0.32 (0.01–9.24)	0.29 (0.01–9.63)	0.26 (0.00–14.59)	0.26 (0.01–12.29)	0.24 (0.01–7.50)	0.26 (0.01–4.14)	0.22 (0.01–4.35)	0.12 (0.00–2.94)	0.02 (0.00–0.63)
1.69 (0.05–51.73)	1.39 (0.07–26.62)	1.85 (0.04–112.04)	1.30 (0.04–43.56)	1.22 (0.03–62.32)	Gefitinib_Chemotherapy	0.78 (0.04–15.86)	0.65 (0.04–11.51)	0.71 (0.01–43.09)	0.42 (0.01–12.00)	0.39 (0.02–9.22)	0.35 (0.01–9.89)	0.32 (0.01–15.01)	0.32 (0.01–12.51)	0.29 (0.01–7.56)	0.31 (0.02–4.07)	0.26 (0.02–4.22)	0.15 (0.01–2.91)	0.03 (0.00–0.62)
2.17 (0.19–24.94)	1.78 (0.31–10.02)	2.37 (0.10–68.94)	1.66 (0.09–30.29)	1.56 (0.06–45.85)	1.28 (0.06–26.59)	Dacomitinib	0.84 (0.14–5.21)	0.90 (0.03–32.71)	0.54 (0.04–7.58)	0.50 (0.07–4.01)	0.46 (0.05–4.65)	0.41 (0.02–7.44)	0.42 (0.03–6.71)	0.37 (0.03–4.78)	0.40 (0.08–1.96)	0.34 (0.09–1.43)	0.19 (0.04–0.91)	0.04 (0.01–0.19)
2.60 (0.21–29.83)	2.12 (0.34–12.17)	2.81 (0.13–74.06)	2.00 (0.13–28.89)	1.85 (0.09–46.66)	1.53 (0.09–26.14)	1.19 (0.19–7.03)	Erlotinib_Chemotherapy	1.08 (0.03–33.01)	0.64 (0.05–7.35)	0.60 (0.09–4.07)	0.54 (0.06–4.77)	0.50 (0.02–9.90)	0.50 (0.03–6.99)	0.44 (0.04–4.57)	0.48 (0.13–1.59)	0.41 (0.12–1.34)	0.23 (0.03–1.37)	0.04 (0.01–0.31)
2.39 (0.05–121.13)	1.96 (0.06–66.56)	2.63 (0.03–245.09)	1.84 (0.03–102.86)	1.74 (0.02–140.09)	1.42 (0.02–88.33)	1.11 (0.03–39.55)	0.93 (0.03–29.58)	Osimertinib_Bevacizumab	0.59 (0.02–14.31)	0.55 (0.01–22.18)	0.50 (0.01–23.31)	0.46 (0.01–34.13)	0.46 (0.01–28.49)	0.41 (0.03–4.92)	0.44 (0.02–10.80)	0.38 (0.01–10.92)	0.21 (0.01–7.32)	0.04 (0.00–1.52)
4.03 (0.18–92.70)	3.31 (0.24–45.17)	4.42 (0.11–210.21)	3.10 (0.13–78.66)	2.92 (0.09–115.16)	2.39 (0.08–68.80)	1.87 (0.13–27.10)	1.56 (0.14–18.89)	1.68 (0.07–40.51)	Osimertinib_Chemotherapy	0.93 (0.06–15.78)	0.85 (0.04–17.03)	0.77 (0.02–27.56)	0.77 (0.03–22.80)	0.69 (0.09–5.12)	0.75 (0.09–6.32)	0.63 (0.06–6.87)	0.36 (0.03–4.89)	0.07 (0.00–1.06)
4.35 (0.28–62.73)	3.57 (0.42–27.67)	4.76 (0.19–139.31)	3.35 (0.16–65.71)	3.12 (0.11–99.38)	2.58 (0.11–58.57)	2.00 (0.25–15.02)	1.68 (0.25–11.32)	1.82 (0.05–69.76)	1.08 (0.06–16.93)	Erlotinib_Bevacizumab	0.91 (0.09–9.48)	0.83 (0.03–20.18)	0.84 (0.05–13.64)	0.75 (0.05–10.69)	0.80 (0.13–4.66)	0.68 (0.15–2.98)	0.39 (0.04–3.13)	0.07 (0.01–0.66)
4.76 (0.25–85.96)	3.90 (0.36–39.20)	5.18 (0.17–181.65)	3.65 (0.15–87.40)	3.41 (0.10–130.00)	2.84 (0.10–77.31)	2.19 (0.22–21.44)	1.84 (0.21–16.54)	1.99 (0.04–88.95)	1.18 (0.06–22.96)	1.09 (0.11–11.61)	Erlotinib_Sunitinib	0.91 (0.03–26.81)	0.91 (0.05–18.09)	0.82 (0.04–14.45)	0.88 (0.11–6.87)	0.74 (0.12–4.63)	0.42 (0.04–4.41)	0.08 (0.01–0.92)
5.24 (0.18–149.96)	4.29 (0.24–76.50)	5.73 (0.10–394.49)	4.03 (0.09–176.55)	3.79 (0.07–241.09)	3.10 (0.07–149.53)	2.42 (0.13–43.65)	2.01 (0.10–44.91)	2.17 (0.03–164.86)	1.30 (0.04–47.69)	1.20 (0.05–32.77)	1.10 (0.04–34.63)	Lazertinib	1.01 (0.02–44.23)	0.90 (0.03–30.20)	0.96 (0.05–17.32)	0.82 (0.05–15.19)	0.47 (0.04–5.28)	0.09 (0.00–1.56)
5.23 (0.19–136.67)	4.28 (0.25–67.45)	5.71 (0.14–268.74)	4.01 (0.11–134.35)	3.79 (0.08–190.35)	3.10 (0.08–114.21)	2.40 (0.15–37.72)	2.01 (0.14–29.29)	2.19 (0.04–129.56)	1.30 (0.04–35.57)	1.20 (0.07–19.98)	1.10 (0.06–21.70)	0.99 (0.02–40.51)	Erlotinib_Ramucirumab	0.90 (0.03–22.33)	0.96 (0.07–12.36)	0.81 (0.08–8.87)	0.47 (0.03–7.57)	0.09 (0.00–1.56)
5.81 (0.28–123.32)	4.77 (0.39–58.27)	6.39 (0.17–283.07)	4.48 (0.19–104.07)	4.19 (0.13–153.48)	3.44 (0.13–93.14)	2.69 (0.21–35.10)	2.25 (0.22–24.75)	2.43 (0.20–29.80)	1.44 (0.20–10.75)	1.34 (0.09–20.80)	1.22 (0.07–22.56)	1.11 (0.03–37.43)	1.11 (0.04–30.13)	Osimertinib	1.07 (0.14–8.08)	0.91 (0.10–8.97)	0.52 (0.04–6.38)	0.10 (0.01–1.39)
5.44 (0.55–52.70)	4.44 (1.00–19.67)	5.88 (0.30–147.75)	4.18 (0.37–46.22)	3.87 (0.24–78.22)	3.21 (0.25–43.02)	2.50 (0.51–12.46)	2.09 (0.63–7.49)	2.26 (0.09–56.62)	1.34 (0.16–11.41)	1.25 (0.21–7.86)	1.14 (0.15–9.37)	1.04 (0.06–18.20)	1.04 (0.08–14.04)	0.93 (0.12–7.01)	Chemotherapy	0.85 (0.32–2.39)	0.48 (0.10–2.20)	0.09 (0.01–0.53)
6.38 (0.63–59.35)	5.23 (1.16–21.93)	6.93 (0.40–145.65)	4.91 (0.35–65.19)	4.57 (0.23–105.53)	3.78 (0.24–59.20)	2.95 (0.70–11.75)	2.47 (0.74–8.28)	2.66 (0.09–74.87)	1.58 (0.15–16.35)	1.47 (0.34–6.57)	1.34 (0.22–8.25)	1.22 (0.07–20.97)	1.23 (0.11–13.10)	1.09 (0.11–10.14)	1.18 (0.42–3.14)	Erlotinib	0.57 (0.11–2.56)	0.11 (0.02–0.56)
11.26 (1.10–116.51)	9.20 (1.97–44.01)	12.30 (0.49–382.94)	8.69 (0.49–153.82)	8.11 (0.34–234.38)	6.65 (0.34–136.86)	5.18 (1.09–25.73)	4.33 (0.73–28.84)	4.68 (0.14–170.69)	2.79 (0.20–39.03)	2.58 (0.32–23.31)	2.36 (0.23–27.01)	2.14 (0.19–24.85)	2.15 (0.13–38.76)	1.93 (0.16–24.84)	2.08 (0.46–9.82)	1.76 (0.39–8.70)	Gefitinib	0.19 (0.04–0.93)
58.43 (5.52–697.51)	47.82 (9.37–276.29)	64.20 (2.43–2263.48)	45.02 (2.32–967.44)	42.33 (1.59–1421.34)	34.59 (1.61–841.29)	26.88 (5.31–158.24)	22.53 (3.25–187.43)	24.33 (0.66–1045.65)	14.48 (0.94–252.53)	13.44 (1.52–147.14)	12.26 (1.09–165.86)	11.12 (0.64–219.26)	11.15 (0.64–232.77)	10.02 (0.72–159.49)	10.79 (1.90–69.10)	9.14 (1.78–57.02)	5.20 (1.08–27.69)	Placebo

Supplementary Figure6. League tables of subgroup analyses for any-grade oral mucositis: (A) first-line treatment; (B) second-line or later treatment (≥2 lines); (C) exclusively Asian populations; and (D) non-exclusively Asian populations.
