# Supplementary material for: Oral mucositis risk with EGFR tyrosine kinase inhibitor monotherapies and combination regimens in non-small cell lung cancer: a systematic review and network meta-analysis
Source: Front Immunol. 2026 Sep 18;17:1957825. doi: 10.3389/fimmu.2026.1957825 (PMC13631102; doi:10.3389/fimmu.2026.1957825)
Supplement: Supplementary file 7 [file Table1.docx]

**Supplementary Tables**

**Supplementary Table S1.** Complete electronic search strategies for the five databases (from inception to June 13, 2026).

| **Search line** | **Search strategy** | **Records** |
| --- | --- | --- |
| **PubMed** | | |
| #1 | ((randomized controlled trial[Publication Type]) OR (randomized[Title/Abstract])) OR (placebo[Title/Abstract]) | 1,202,707 |
| #2 | ("Carcinoma, Non-Small-Cell Lung"[Mesh]) AND ((((((((((((Carcinoma, Non-Small-Cell Lung[Title/Abstract]) OR (Carcinoma, Non Small Cell Lung[Title/Abstract])) OR (Carcinomas, Non-Small-Cell Lung[Title/Abstract])) OR (Lung Carcinoma, Non-Small-Cell[Title/Abstract])) OR (Lung Carcinomas, Non-Small-Cell[Title/Abstract])) OR (Non-Small-Cell Lung Carcinomas[Title/Abstract])) OR (Carcinoma, Non-Small Cell Lung[Title/Abstract])) OR (Non-Small Cell Lung Cancer[Title/Abstract])) OR (Non-Small-Cell Lung Carcinoma[Title/Abstract])) OR (Non Small Cell Lung Carcinoma[Title/Abstract])) OR (Nonsmall Cell Lung Cancer[Title/Abstract])) OR (Non-Small Cell Lung Carcinoma[Title/Abstract])) | 68,300 |
| #3 | ("Gefitinib"[Mesh]) OR ((((Gefitinib[Title/Abstract]) OR (ZD1839[Title/Abstract])) OR (ZD 1839[Title/Abstract])) OR (Iressa[Title/Abstract])) | 9,577 |
| #4 | ("Erlotinib Hydrochloride"[Mesh]) OR ((((((((((((((((((Erlotinib Hydrochloride[Title/Abstract]) OR (Hydrochloride, Erlotinib[Title/Abstract])) OR (Erlotinib HCl[Title/Abstract])) OR (HCl, Erlotinib[Title/Abstract])) OR (Erlotinib[Title/Abstract])) OR (OSI-774[Title/Abstract])) OR (OSI774[Title/Abstract])) OR (OSI 774[Title/Abstract])) OR (Tarceva[Title/Abstract])) OR (CP 358774[Title/Abstract])) OR (358774, CP[Title/Abstract])) OR (CP-358774[Title/Abstract])) OR (CP358774[Title/Abstract])) OR (CP 358,774[Title/Abstract])) OR (CP-358,774[Title/Abstract])) OR (CP358,774[Title/Abstract])) OR (11C-erlotinib[Title/Abstract])) OR (11C erlotinib[Title/Abstract])) | 8,966 |
| #5 | icotinib[Title/Abstract] | 437 |
| #6 | ("Afatinib"[Mesh]) OR (((((((((Afatinib[Title/Abstract]) OR (BIBW 2992[Title/Abstract])) OR (BIBW-2992[Title/Abstract])) OR (BIBW2992[Title/Abstract])) OR (Gilotrif[Title/Abstract])) OR (Afatinib Dimaleate[Title/Abstract])) OR (BIBW 2992 MA2[Title/Abstract])) OR (BIBW2992 MA2[Title/Abstract])) OR (Afatinib Maleate[Title/Abstract])) | 2,656 |
| #7 | (((dacomitinib[Title/Abstract]) OR (PF 00299804[Title/Abstract])) OR (PF-00299804[Title/Abstract])) OR (PF00299804[Title/Abstract]) | 449 |
| #8 | Mifanertinib[Title/Abstract] | 1 |
| #9 | ((((((((osimertinib[Title/Abstract]) OR (AZD9291[Title/Abstract])) OR (AZD-9291[Title/Abstract])) OR (osimertinib mesylate[Title/Abstract])) OR (mereletinib mesylate[Title/Abstract])) OR (osimertinib mesilate[Title/Abstract])) OR (mereletinib mesilate[Title/Abstract])) OR (AZD-9291 mesylate[Title/Abstract])) OR (AZD9291 mesylate[Title/Abstract]) | 4,355 |
| #10 | ((aumolertinib[Title/Abstract]) OR (almonertinib[Title/Abstract])) OR (HS-10296[Title/Abstract]) | 199 |
| #11 | (((furmonertinib[Title/Abstract]) OR (furmonertinib mesylate[Title/Abstract])) OR (Ivesa[Title/Abstract])) OR (AST2818[Title/Abstract]) | 128 |
| #12 | (Befotertinib[Title/Abstract]) OR (D-0316[Title/Abstract]) | 27 |
| #13 | (rezivertinib[Title/Abstract]) OR (BPI-7711[Title/Abstract]) | 18 |
| #14 | ((Rilertinib[Title/Abstract]) OR (Oritinib[Title/Abstract])) OR (SH-1028[Title/Abstract]) | 10 |
| #15 | Limertinib[Title/Abstract] | 12 |
| #16 | (((((((abivertinib[Title/Abstract]) OR (avitinib[Title/Abstract])) OR (AC0010[Title/Abstract])) OR (abivertinib maleate[Title/Abstract])) OR (abivertinib maleate dihydrate[Title/Abstract])) OR (abivertinib maleate anhydrous[Title/Abstract])) OR (avitinib maleate[Title/Abstract])) OR (AC-0010 maleate dihydrate[Title/Abstract]) | 43 |
| #17 | ((sunvozertinib[Title/Abstract]) OR (Zegfrovy[Title/Abstract])) OR (DZD9008[Title/Abstract]) | 51 |
| #18 | ((Mobocertinib[Title/Abstract]) OR (TAK-788[Title/Abstract])) OR (Exkivity[Title/Abstract]) | 123 |
| #19 | (((lazertinib[Title/Abstract]) OR (Leclaza[Title/Abstract])) OR (Lazcluze[Title/Abstract])) OR (lazertinib mesylate[Title/Abstract]) | 168 |
| #20 | #3 OR #4 OR #5 OR #6 OR #7 OR #8 OR #9 OR #10 OR #11 OR #12 OR #13 OR #14 OR #15 OR #16 OR #17 OR #18 OR #19 | 21,249 |
| **#21** | **#1 AND #2 AND #20** | **898** |
| **Embase** | | |
| #1 | 'gefitinib'/exp | 32,931 |
| #2 | 'gefitinib':ab,ti OR 'zd1839':ab,ti OR 'zd 1839':ab,ti OR 'iressa':ab,ti | 14,488 |
| #3 | 'erlotinib'/exp | 36,647 |
| #4 | 'erlotinib hydrochloride':ab,ti OR 'hydrochloride, erlotinib':ab,ti OR 'erlotinib hcl':ab,ti OR 'hcl, erlotinib':ab,ti OR 'erlotinib':ab,ti OR 'osi-774':ab,ti OR 'osi774':ab,ti OR 'osi 774':ab,ti OR 'tarceva':ab,ti OR 'cp 358774':ab,ti OR '358774, cp':ab,ti OR 'cp-358774':ab,ti OR 'cp358774':ab,ti OR 'cp 358,774':ab,ti OR 'cp-358,774':ab,ti OR 'cp358,774':ab,ti OR '11c-erlotinib':ab,ti OR '11c erlotinib':ab,ti | 16,066 |
| #5 | 'icotinib'/exp | 1,774 |
| #6 | 'icotinib':ab,ti | 815 |
| #7 | 'afatinib'/exp | 10,741 |
| #8 | 'afatinib':ab,ti OR 'bibw 2992':ab,ti OR 'bibw-2992':ab,ti OR 'bibw2992':ab,ti OR 'gilotrif':ab,ti OR 'afatinib dimaleate':ab,ti OR 'bibw 2992 ma2':ab,ti OR 'bibw2992 ma2':ab,ti OR 'afatinib maleate':ab,ti | 5,108 |
| #9 | 'dacomitinib'/exp | 2,412 |
| #10 | 'dacomitinib':ab,ti OR 'pf 00299804':ab,ti OR 'pf-00299804':ab,ti OR 'pf00299804':ab,ti | 887 |
| #11 | 'mifanertinib'/exp OR 'mifanertinib':ab,ti | 1 |
| #12 | 'osimertinib'/exp | 12,679 |
| #13 | 'osimertinib':ab,ti OR 'azd9291':ab,ti OR 'azd-9291':ab,ti OR 'osimertinib mesylate':ab,ti OR 'mereletinib mesylate':ab,ti OR 'osimertinib mesilate':ab,ti OR 'mereletinib mesilate':ab,ti OR 'azd-9291 mesylate':ab,ti OR 'azd9291 mesylate':ab,ti | 8,148 |
| #14 | 'lazertinib'/exp | 631 |
| #15 | 'lazertinib':ab,ti OR 'leclaza':ab,ti OR 'lazcluze':ab,ti OR 'lazertinib mesylate':ab,ti | 374 |
| #16 | 'aumolertinib'/exp OR 'aumolertinib' OR 'aumolertinib':ab,ti OR 'almonertinib':ab,ti OR 'hs-10296':ab,ti | 569 |
| #17 | 'firmonertinib'/exp OR 'firmonertinib' OR 'furmonertinib':ab,ti OR 'furmonertinib mesylate':ab,ti OR 'ivesa':ab,ti OR 'ast2818':ab,ti | 505 |
| #18 | 'befotertinib'/exp OR 'befotertinib' OR 'befotertinib':ab,ti OR 'd-0316':ab,ti | 67 |
| #19 | 'rezivertinib'/exp OR 'rezivertinib' OR 'rezivertinib':ab,ti OR 'bpi-7711':ab,ti | 53 |
| #20 | 'rilertinib':ab,ti OR 'oritinib':ab,ti OR 'sh-1028':ab,ti | 22 |
| #21 | 'limertinib'/exp OR 'limertinib' OR 'limertinib':ab,ti | 28 |
| #22 | 'abivertinib'/exp OR 'abivertinib' OR 'abivertinib':ab,ti OR 'avitinib':ab,ti OR 'ac0010':ab,ti OR 'abivertinib maleate':ab,ti OR 'abivertinib maleate dihydrate':ab,ti OR 'abivertinib maleate anhydrous':ab,ti OR 'avitinib maleate':ab,ti OR 'ac-0010 maleate dihydrate':ab,ti | 121 |
| #23 | 'sunvozertinib'/exp OR 'sunvozertinib' OR 'sunvozertinib':ab,ti OR 'zegfrovy':ab,ti OR 'dzd9008':ab,ti | 173 |
| #24 | 'mobocertinib'/exp OR 'mobocertinib' OR 'mobocertinib':ab,ti OR 'tak-788':ab,ti OR 'exkivity':ab,ti | 501 |
| #25 | #1 OR #2 OR #3 OR #4 OR #5 OR #6 OR #7 OR #8 OR #9 OR #10 OR #11 OR #12 OR #13 OR #14 OR #15 OR #16 OR #17 OR #18 OR #19 OR #20 OR #21 OR #22 OR #23 OR #24 | 67,784 |
| #26 | 'non small cell lung cancer'/exp | 279,749 |
| #27 | 'carcinoma, non-small-cell lung':ab,ti OR 'carcinoma, non small cell lung':ab,ti OR 'carcinomas, non-small-cell lung':ab,ti OR 'lung carcinoma, non-small-cell':ab,ti OR 'lung carcinomas, non-small-cell':ab,ti OR 'non-small-cell lung carcinomas':ab,ti OR 'non-small cell lung cancer':ab,ti OR 'non-small-cell lung carcinoma':ab,ti OR 'non small cell lung carcinoma':ab,ti OR 'nonsmall cell lung cancer':ab,ti | 170,030 |
| #28 | #26 OR #27 | 292,623 |
| #29 | 'randomized controlled trial':ab,ti OR randomized:ab,ti OR placebo:ab,ti | 1,611,628 |
| **#30** | **#25 AND #28 AND #29** | **3,522** |
| **Web of Science Core Collection** | | |
| #1 | Carcinoma, Non-Small-Cell Lung (Topic) OR Carcinoma, Non-Small-Cell Lung (Topic) OR Lung Carcinoma, Non-Small-Cell (Topic) OR Lung Carcinomas, Non-Small-Cell (Topic) OR Non-Small-Cell Lung Carcinomas (Topic) OR Carcinoma, Non-Small Cell Lung (Topic) OR Non-Small Cell Lung Cancer (Topic) OR Non-Small-Cell Lung Carcinoma (Topic) OR Non Small Cell Lung Carcinoma (Topic) OR Nonsmall Cell Lung Cancer (Topic) OR Non-Small Cell Lung Carcinoma (Topic) | 115,697 |
| #2 | randomized controlled trial (Topic) OR randomized (Topic) OR placebo (Topic) | 966,191 |
| #3 | Gefitinib (Topic) OR ZD1839 (Topic) OR ZD 1839 (Topic) OR Iressa (Topic) | 15,423 |
| #4 | Erlotinib Hydrochloride (Topic) OR Hydrochloride, Erlotinib (Topic) OR Erlotinib HCl (Topic) OR HCl, Erlotinib (Topic) OR Erlotinib (Topic) OR OSI-774 (Topic) OR OSI774 (Topic) OR OSI 774 (Topic) OR Tarceva (Topic) OR CP 358774 (Topic) OR 358774, CP (Topic) OR CP-358774 (Topic) OR CP358774 (Topic) OR CP 358,774 (Topic) OR CP-358,774 (Topic) OR CP358,774 (Topic) OR 11C-erlotinib (Topic) OR 11C erlotinib (Topic) | 13,562 |
| #5 | icotinib (Topic) | 560 |
| #6 | Afatinib (Topic) OR BIBW 2992 (Topic) OR BIBW-2992 (Topic) OR BIBW2992 (Topic) OR Gilotrif (Topic) OR Afatinib Dimaleate (Topic) OR BIBW 2992 MA2 (Topic) OR BIBW2992 MA2 (Topic) OR Afatinib Maleate (Topic) | 3,885 |
| #7 | dacomitinib (Topic) OR PF 00299804 (Topic) OR PF-00299804 (Topic) OR PF00299804 (Topic) | 617 |
| #8 | Mifanertinib (Topic) | 1 |
| #9 | osimertinib (Topic) OR AZD9291 (Topic) OR AZD-9291 (Topic) OR osimertinib mesylate (Topic) OR mereletinib mesylate (Topic) OR osimertinib mesilate (Topic) OR mereletinib mesilate (Topic) OR AZD-9291 mesylate (Topic) OR AZD9291 mesylate (Topic) | 6,698 |
| #10 | lazertinib (Topic) OR Leclaza (Topic) OR GNS-1480 (Topic) OR JNJ-73841937-ZCY (Topic) OR YH25448AM (Topic) OR Lazcluze (Topic) OR JNJ-73841937-AAA (Topic) OR R-605024 (Topic) OR YH-25448 (Topic) OR lazertinib mesylate (Topic) | 260 |
| #11 | aumolertinib (Topic) OR almonertinib (Topic) OR HS-10296 (Topic) | 340 |
| #12 | furmonertinib (Topic) OR AST2818 (Topic) OR furmonertinib mesylate (Topic) OR furmonertinib (Topic) OR Ivesa (Topic) | 182 |
| #13 | Befotertinib (Topic) OR D-0316 (Topic) | 30 |
| #14 | rezivertinib (Topic) OR BPI-7711 (Topic) | 27 |
| #15 | Rilertinib (Topic) OR Oritinib (Topic) OR SH-1028 (Topic) | 12 |
| #16 | Limertinib (Topic) | 14 |
| #17 | abivertinib (Topic) OR avitinib (Topic) OR abivertinib maleate (Topic) OR AC0010 (Topic) OR abivertinib maleate dihydrate (Topic) OR abivertinib maleate anhydrous (Topic) OR avitinib maleate (Topic) OR AC-0010 maleate dihydrate (Topic) | 52 |
| #18 | sunvozertinib (Topic) OR Zegfrovy (Topic) OR DZD9008 (Topic) | 67 |
| #19 | Mobocertinib (Topic) OR TAK-788 (Topic) OR Exkivity (Topic) | 160 |
| #20 | #3 OR #4 OR #5 OR #6 OR #7 OR #8 OR #9 OR #10 OR #11 OR #12 OR #13 OR #14 OR #15 OR #16 OR #17 OR #18 OR #19 | 31,041 |
| **#21** | **#20 AND #1 AND #2** | **1,981** |
| **Cochrane Library** | | |
| #1 | MeSH descriptor: [Carcinoma, Non-Small-Cell Lung] explode all trees | 7,108 |
| #2 | (Carcinoma, Non-Small-Cell Lung):ti,ab,kw OR (Carcinoma, Non Small Cell Lung):ti,ab,kw OR (Carcinomas, Non-Small-Cell Lung):ti,ab,kw OR (Lung Carcinoma, Non-Small-Cell):ti,ab,kw OR (Lung Carcinomas, Non-Small-Cell):ti,ab,kw OR (Non-Small-Cell Lung Carcinomas):ti,ab,kw OR (Carcinoma, Non-Small Cell Lung):ti,ab,kw OR (Non-Small Cell Lung Cancer):ti,ab,kw OR (Non-Small-Cell Lung Carcinoma):ti,ab,kw OR (Non Small Cell Lung Carcinoma):ti,ab,kw OR (Nonsmall Cell Lung Cancer):ti,ab,kw OR (Non-Small Cell Lung Carcinoma):ti,ab,kw | 19,119 |
| #3 | #1 OR #2 | 19,119 |
| #4 | MeSH descriptor: [Randomized Controlled Trial] explode all trees | 34 |
| #5 | (randomized controlled trial ):ti,ab,kw OR (randomized):ti,ab,kw OR (placebo):ti,ab,kw | 1,452,542 |
| #6 | #4 OR #5 | 1,452,542 |
| #7 | MeSH descriptor: [Gefitinib] explode all trees | 419 |
| #8 | (Gefitinib):ti,ab,kw OR (ZD1839):ti,ab,kw OR (ZD 1839):ti,ab,kw OR (Iressa):ti,ab,kw | 1,225 |
| #9 | #7 OR #8 | 1,225 |
| #10 | MeSH descriptor: [Erlotinib Hydrochloride] explode all trees | 703 |
| #11 | (Erlotinib Hydrochloride):ti,ab,kw OR (Hydrochloride, Erlotinib):ti,ab,kw OR (Erlotinib HCl):ti,ab,kw OR (HCl, Erlotinib):ti,ab,kw OR (Erlotinib):ti,ab,kw OR (OSI-774):ti,ab,kw OR (OSI774):ti,ab,kw OR (OSI 774):ti,ab,kw OR (Tarceva):ti,ab,kw OR (CP 358774):ti,ab,kw OR (358774, CP):ti,ab,kw OR (CP-358774):ti,ab,kw OR (CP358774):ti,ab,kw OR (CP 358,774):ti,ab,kw OR (CP-358,774):ti,ab,kw OR (CP358,774):ti,ab,kw OR (11C-erlotinib):ti,ab,kw OR (11C erlotinib):ti,ab,kw | 1,893 |
| #12 | #10 OR #11 | 1,893 |
| #13 | (Icotinib):ti,ab,kw | 146 |
| #14 | MeSH descriptor: [Afatinib] explode all trees | 121 |
| #15 | (Afatinib):ti,ab,kw OR (BIBW 2992):ti,ab,kw OR (BIBW-2992):ti,ab,kw OR (BIBW2992):ti,ab,kw OR (Gilotrif):ti,ab,kw OR (Afatinib Dimaleate):ti,ab,kw OR (BIBW 2992 MA2):ti,ab,kw OR (BIBW2992 MA2):ti,ab,kw OR (Afatinib Maleate):ti,ab,kw | 508 |
| #16 | #14 OR #15 | 508 |
| #17 | (Dacomitinib):ti,ab,kw OR (PF 00299804):ti,ab,kw OR (PF-00299804):ti,ab,kw OR (PF00299804):ti,ab,kw | 109 |
| #18 | (Mifanertinib):ti,ab,kw | 0 |
| #19 | (Osimertinib):ti,ab,kw OR (AZD9291):ti,ab,kw OR (AZD-9291):ti,ab,kw OR (osimertinib mesylate):ti,ab,kw OR (mereletinib mesylate):ti,ab,kw OR (osimertinib mesilate):ti,ab,kw OR (mereletinib mesilate):ti,ab,kw OR (AZD-9291 mesylate):ti,ab,kw OR (AZD9291 mesylate):ti,ab,kw | 697 |
| #20 | (Lazertinib):ti,ab,kw OR (Leclaza):ti,ab,kw OR (Lazcluze):ti,ab,kw OR (lazertinib mesylate):ti,ab,kw | 105 |
| #21 | (Aumolertinib):ti,ab,kw OR (almonertinib):ti,ab,kw OR (HS-10296):ti,ab,kw | 75 |
| #22 | (Furmonertinib):ti,ab,kw OR (furmonertinib mesylate):ti,ab,kw OR (Ivesa):ti,ab,kw OR (AST2818):ti,ab,kw | 47 |
| #23 | (Befotertinib):ti,ab,kw OR (D-0316):ti,ab,kw | 9 |
| #24 | (rezivertinib):ti,ab,kw OR (BPI-7711):ti,ab,kw | 6 |
| #25 | (Rilertinib):ti,ab,kw OR (Oritinib):ti,ab,kw OR (SH-1028):ti,ab,kw | 4 |
| #26 | (Limertinib):ti,ab,kw | 7 |
| #27 | (abivertinib):ti,ab,kw OR (avitinib):ti,ab,kw OR (AC0010):ti,ab,kw OR (abivertinib maleate):ti,ab,kw OR (abivertinib maleate dihydrate):ti,ab,kw OR (abivertinib maleate anhydrous):ti,ab,kw OR (avitinib maleate):ti,ab,kw OR (AC-0010 maleate dihydrate):ti,ab,kw | 6 |
| #28 | (sunvozertinib):ti,ab,kw OR (Zegfrovy):ti,ab,kw OR (DZD9008):ti,ab,kw | 10 |
| #29 | (Mobocertinib):ti,ab,kw OR (TAK-788):ti,ab,kw OR (Exkivity):ti,ab,kw | 14 |
| #30 | #9 OR #12 OR #13 OR #16 OR #17 OR #18 OR #19 OR #20 OR #21 OR #22 OR #23 OR #24 OR #25 OR #26 OR #27 OR #28 OR #29 | 3,895 |
| **#31** | **#30 AND #3 AND #6** | **1,977** |
| **ClinicalTrials.gov** | | |
| **Final query** | "Carcinoma, Non-Small-Cell Lung" OR "Carcinoma, Non Small Cell Lung" OR "Carcinomas, Non-Small-Cell Lung" OR "Lung Carcinoma, Non-Small-Cell" OR "Lung Carcinomas, Non-Small-Cell" OR "Non-Small-Cell Lung Carcinomas" OR "Carcinoma, Non-Small Cell Lung" OR "Non Small Cell Lung Cancer" OR "Non-Small-Cell Lung Carcinoma" OR "Non Small Cell Lung Carcinoma" OR "Nonsmall Cell Lung Cancer" \| "Gefitinib" OR "ZD1839" OR "ZD 1839" OR "Iressa" OR "Erlotinib Hydrochloride" OR "Hydrochloride, Erlotinib" OR "Erlotinib HCl" OR "HCl, Erlotinib" OR "Erlotinib" OR "OSI-774" OR "OSI774" OR "OSI 774" OR "Tarceva" OR "CP 358774" OR "358774, CP" OR "CP-358774" OR "CP358774" OR "CP 358,774" OR "CP-358,774" OR "CP358,774" OR "11C-erlotinib" OR "11C erlotinib" OR "Icotinib" OR "Afatinib" OR "BIBW 2992" OR "BIBW-2992" OR "BIBW2992" OR "Gilotrif" OR "Afatinib Dimaleate" OR "BIBW 2992 MA2" OR "BIBW2992 MA2" OR "Afatinib Maleate" OR "dacomitinib" OR "PF 00299804" OR "PF-00299804" OR "PF00299804" OR "Mifanertinib" OR "osimertinib" OR "AZD9291" OR "AZD-9291" OR "osimertinib mesylate" OR "mereletinib mesylate" OR "osimertinib mesilate" OR "mereletinib mesilate" OR "AZD-9291 mesylate" OR "AZD9291 mesylate" OR "lazertinib" OR "Leclaza" OR "Lazcluze" OR "lazertinib mesylate" OR "aumolertinib" OR "almonertinib" OR "HS-10296" OR "furmonertinib" OR "furmonertinib mesylate" OR "Ivesa" OR "AST2818" OR "Befotertinib" OR "D-0316" OR "rezivertinib" OR "BPI-7711" OR "Rilertinib" OR "Oritinib" OR "SH-1028" OR "Limertinib" OR "abivertinib" OR "avitinib" OR "AC0010" OR "abivertinib maleate" OR "abivertinib maleate dihydrate" OR "abivertinib maleate anhydrous" OR "avitinib maleate" OR "AC-0010 maleate dihydrate" OR "sunvozertinib" OR "Zegfrovy" OR "DZD9008" OR "Mobocertinib" OR "TAK-788" OR "Exkivity" Filters: Completed or terminated studies; Phase 2, 3, or 4; interventional studies; studies with results. | **237** |

**Supplementary Table S2.** Baseline characteristics of the included randomized controlled trials.

| **Study, year** | **Registration no.** | **Phase** | **Region** | **Population** | **Line** | **Experimental arm(s)** | **Control arm(s)** | **Randomized, n** | **Median age, y** | **Female, n** | **OM outcomes** | **Original oral AE terminology** | **AE grading system/version** |
| --- | --- | --- | --- | --- | --- | --- | --- | --- | --- | --- | --- | --- | --- |
| LUX-Lung 6, 2014 (Wu et al.) | NCT01121393 | III | Multicountry (Asian) | IIIB/IV NSCLC | 1L | Afatinib 40 mg PO QD | Gemcitabine 1000 mg/m² IV d1,8 + cisplatin 75 mg/m² IV d1 q3w, ≤6 cycles (chemo) | 242 / 122 | 56.7 / 55.6 | 155 / 83 | Any-grade only | Stomatitis | NCI CTCAE v3.0 |
| LUX‑Lung 3, 2023 (Sequist et al.) | NCT00949650 | III | Multicountry | IIIB/IV adenocarcinoma | 1L | Afatinib 40 mg PO QD | Cisplatin 75 mg/m² IV + pemetrexed 500 mg/m² IV d1 q3w, ≤6 cycles (chemo) | 230 / 115 | 61.5 / 61 | 147 / 77 | Both | Stomatitis | NCI CTCAE v3.0 |
| LUX-Lung 8, 2015 (Soria et al.) | NCT01523587 | III | Multicountry | IIIB/IV squamous cell carcinoma | 2L | Afatinib 40 mg PO QD | Erlotinib 150 mg PO QD | 398 / 397 | 64.9 / 63.4 | 63 / 66 | Both | Stomatitis | NCI CTCAE v3.0 |
| LUX-Lung 7, 2017 (Paz-Ares et al.) | NCT01466660 | II | Multicountry | IIIB/IV NSCLC | 1L | Afatinib 40 mg PO QD | Gefitinib 250 mg PO QD | 160 / 159 | 61.7 / 63 | 91 / 106 | Both | Stomatitis | NCI CTCAE v3.0 |
| LUX‑Lung 1, 2012 (Mille et al.) | NCT00656136 | III | Multicountry | IIIB/IV adenocarcinoma | 3L | Afatinib 50mg PO QD | Placebo PO QD | 390 / 195 | 58 / 59 | 231 / 117 | Both | Stomatitis | NCI CTCAE v3.0 |
| LUX-Lung 5, 2016 (Schuler et al.) | NCT01085136 | III | Multicountry | IIIB/IV NSCLC | 4L | Afatinib 40 mg PO QD + paclitaxel 80 mg/m² IV weekly (7 weeks on/1 week off) | Investigator's-choice chemotherapy (regimen/dose NR) (chemo) | 134 / 68 | 60 / 60.5 | 65 / 34 | Both | Stomatitis | NCI CTCAE v3.0 |
| AENEAS, 2022 (Lu et al.) | NCT03849768 | III | China | Advanced/metastatic NSCLC | 1L | Aumolertinib 110 mg PO QD | Gefitinib 250 mg PO QD | 214 / 215 | 59 / 62 | 134 / 135 | Any-grade only | Oral mucositis | NCI CTCAE v4.03 |
| Ramalingam et al., 2012 | NCT00769067 | II | Multicountry | Advanced NSCLC | 2L | Dacomitinib 45 mg PO QD (28-day cycle) | Erlotinib 150 mg PO QD (28-day cycle) | 94 / 94 | 60 / 62 | 38 / 39 | Both | Stomatitis | — |
| ARCHER 1009, 2014 (Ramalingam et al.) | NCT01360554 | III | Multicountry | Advanced NSCLC | 2-3L | Dacomitinib 45 mg PO QD (28-day cycle) | Erlotinib 150 mg PO QD (28-day cycle) | 439 / 439 | 64 / 62 | 151 / 162 | Both | Stomatitis | NCI CTCAE v4.0 |
| ARCHER 1050, 2017 (Wu et al.) | NCT01774721 | III | Multicountry | Advanced NSCLC | 1L | Dacomitinib 45 mg PO QD (28-day cycle) | Gefitinib 250 mg PO QD (28-day cycle) | 227 / 225 | 62 / 61 | 146 / 125 | Both | Stomatitis | NCI CTCAE v4.03 |
| NCIC CTG BR.26, 2014 (Ellis et al.) | NCT01000025 | III | Multicountry | Advanced/metastatic NSCLC | ≥2L | Dacomitinib 45 mg PO QD | Placebo PO QD | 480 / 240 | 63.5 / 65.5 | 236 / 120 | Both | Oral mucositis | NCI CTCAE v4.0 |
| Lilenbaum et al., 2008 | NCT00085839 | II | US | IIIB/IV NSCLC | 1L | Erlotinib 150 mg PO QD | Paclitaxel 200 mg/m² IV + carboplatin AUC 6 IV d1 q3w, 4 cycles (chemo) | 52 / 51 | 69 / 67 | 29 / 23 | Both | Stomatitis | NCI CTCAE v3.0 |
| OPTIMAL,CTONG-0802, 2011 (Zhou et al.) | NCT00874419 | III | China | IIIB/IV/recurrent non-squamous NSCLC | 1L | Erlotinib 150 mg PO QD | Gemcitabine 1000 mg/m² IV d1,8 + carboplatin AUC 5 IV d1 q3w, ≤4 cycles (chemo) | 82 / 72 | 57 / 59 | 48 / 43 | Both | Stomatitis | NCI CTCAE v3.0 |
| PDX‑012, 2012 (Kelly et al.) | NCT00606502 | II | Multicountry | IIIB/IV NSCLC | ≥2L | Erlotinib 150 mg PO QD (28-day cycle) | Pralatrexate 230→190 mg/m² IV d1,15 q4w (chemo) | 101 / 100 | 62 / 63 | 33 / 31 | Both | Stomatitis | NCI CTCAE v3.0 |
| TITAN, 2012 (Ciuleanu et al.) | NCT00556322 | III | Multicountry | Advanced/recurrent/metastatic NSCLC | 2L | Erlotinib 150 mg PO QD | Pemetrexed 500 mg/m² IV q3w or docetaxel 75 mg/m² IV q3w (chemo) | 203 / 221 | 59 / 59 | 42 / 61 | Both | Stomatitis | NCI CTCAE v3.0 |
| ENSURE, 2015 (Wu et al.) | NCT01342965 | III | Multicountry (Asian) | IIIB/IV NSCLC | 1L | Erlotinib 150 mg PO QD | Gemcitabine 1250 mg/m² IV d1,8 + cisplatin 75 mg/m² IV d1 q3w, ≤4 cycles (chemo) | 110 / 107 | 56.7 / 55.8 | 68 / 65 | Any-grade only | Stomatitis | NCI CTCAE v4.0 |
| Registry study, 2015 | NCT01652469 | III | Multicountry | IIIB/IV NSCLC | ≥2L | Erlotinib 150 mg PO QD (21-day cycle) | Docetaxel 75 mg/m² IV q3w (chemo) | 38 / 42 | 66.7 / 70.1 | 7 / 7 | Any-grade only | Oral mucositis | NCI CTCAE v4.0 |
| Registry study, 2017 | NCT00126581 | II | US | III/IV adenocarcinoma NSCLC | NR | Erlotinib PO QD d1-21 q3w (dose NR) | Erlotinib PO QD + paclitaxel IV + carboplatin IV d1 q3w, ≤6 cycles (doses NR) | 81 / 100 | 58 / 60 | 49 / 58 | Both | Stomatitis | NCI CTCAE v3.0 |
| TOPICAL, 2012 (Lee et al.) | ISRCTN 77383050 | III | US | IIIB/IV NSCLC | 1L | Erlotinib 150 mg PO QD | Placebo PO QD | 350 / 320 | 77 / 77 | 135 / 126 | Grade ≥3 only | Stomatitis | NCI CTCAE v4.0 |
| Dittrich et al., 2014 | NCT00447057 | II | Multicountry | IIIA/IIIB/IV non-squamous NSCLC | 2L | Erlotinib 150 mg PO QD + pemetrexed 500 mg/m² IV d1 q3w | Pemetrexed 500 mg/m² IV d1 q3w (chemo) | 102 / 102 | 64.2 / 60.2 | 32 / 34 | Both | Stomatitis | NCI CTCAE v3.0 |
| FAST-ACT, 2009 (Mok et al.) | — | II | Multicountry (Asian) | IIIB/IV NSCLC | 1L | Erlotinib 150 mg PO d15-28 + gemcitabine 1250 mg/m² IV d1,8 + cisplatin 75 mg/m² or carboplatin AUC 5 IV d1 q4w | Placebo PO d15-28 + the same platinum-gemcitabine regimen q4w (chemo) | 76 / 78 | 57.5 / 57 | 22 / 24 | Both | Stomatitis | NCI CTCAE v3.0 |
| Registry study, 2011 | NCT01998919 | II | Multicountry | IIIB/IV NSCLC | 1L | Erlotinib 150 mg PO d15-28 + gemcitabine 1250 mg/m² IV d1,8 + cisplatin 75 mg/m² or carboplatin AUC 5 IV d1 q4w | Placebo PO d15-28 + the same platinum-gemcitabine regimen q4w (chemo) | 76 / 78 | 56.4 / 56.5 | 22 / 24 | Any-grade only | Stomatitis | — |
| FASTACT-2, 2013 (Wu et al.) | NCT00883779 | III | Multicountry (Asian) | IIIB/IV NSCLC | 1L | Erlotinib 150 mg PO d15-28 + gemcitabine 1250 mg/m² IV d1,8 + cisplatin 75 mg/m² or carboplatin AUC 5 IV d1 q4w | Placebo PO d15-28 + the same platinum-gemcitabine regimen q4w (chemo) | 226 / 225 | 59 / 57.3 | 94 / 85 | Both | Stomatitis | NCI CTCAE v3.0 |
| Registry study, 2009 | NCT00294762 | II | Multicountry | NSCLC | 1L | Erlotinib 150 mg PO d2-15 + paclitaxel 200 mg/m² IV + carboplatin AUC 6 IV d1 q3w, 4 cycles; then erlotinib QD | Erlotinib 150 mg PO QD | 71 / 72 | 63 / 63 | 31 / 44 | Any-grade only | Stomatitis | — |
| S103, 2013 (Lee et al.) | NCT00550173 | II | Multicountry | Advanced/metastatic nonsquamous NSCLC | 2L | Pemetrexed 500 mg/m² IV d1 + erlotinib 150 mg PO d2-14 q3w | Erlotinib 150 mg PO QD / pemetrexed 500 mg/m² IV d1 q3w (three-arm trial) | 78 / 82 / 80 | 56 / 53.9 / 56.1 | 58 / 54 / 45 | Both | Stomatitis | NCI CTCAE v3.0 |
| FURLONG, 2022 (Shi et al.) | NCT03787992 | III | China | IIIB-IIIC/IV NSCLC | 1L | Furmonertinib 80 mg PO QD (21-day cycle) | Gefitinib 250 mg PO QD (21-day cycle) | 178 / 179 | 59 / 60 | 116 / 111 | Both | Stomatitis | NCI CTCAE v5.0 |
| V-15-32, 2008 (Maruyama et al.) | NCT00252707 | III | Japan | IIIB-IV/postoperative recurrent NSCLC | ≥2L | Gefitinib 250 mg PO QD | Docetaxel 60 mg/m² IV q3w (chemo) | 245 / 244 | NR | 94 / 93 | Both | Stomatitis | NCI CTCAE v2.0 |
| INTEREST, 2008 (Kim et al.) | NCT00076388 | III | Multicountry | Advanced/metastatic NSCLC | ≥2L | Gefitinib 250 mg PO QD | Docetaxel 75 mg/m² IV q3w (chemo) | 733 / 733 | 61 / 60 | 267 / 245 | Both | Stomatitis | NCI CTCAE v2.0 |
| IPASS, 2009 (Mok et al.) | NCT00322452 | III | Multicountry (Asian) | IIIB/IV adenocarcinoma | 1L | Gefitinib 250 mg PO QD | Paclitaxel 200 mg/m² IV + carboplatin AUC 5-6 IV d1 q3w (chemo) | 609 / 608 | 57 / 57 | 484 / 481 | Both | Stomatitis | NCI CTCAE v2.0 |
| WJTOG3405, 2010 (Mitsudomi et al.) | — | III | Japan | IIIB/IV NSCLC or postoperative recurrence NSCLC | 1L | Gefitinib 250 mg PO QD | Docetaxel 60 mg/m² IV + cisplatin 80 mg/m² IV d1 q3w, 3-6 cycles (chemo) | 86 / 86 | NR | 59 / 60 | Both | Stomatitis | NCI CTCAE v3.0 |
| ISTANA, 2010 (Lee et al.) | D7913L00039 | III | South Korea | IIIB/IV NSCLC | 2L | Gefitinib 250 mg PO QD | Docetaxel 75 mg/m² IV d1 q3w (chemo) | 82 / 79 | 57 / 58 | 27 / 34 | Both | Stomatitis | NCI CTCAE v3.0 |
| NEJ002, 2010 (Maemondo et al.) | UMIN C000000376 | III | Japan | IIIB/IV/postoperative relapse NSCLC | 1L | Gefitinib 250 mg PO QD | Paclitaxel 200 mg/m² IV + carboplatin AUC 6 IV d1 q3w (chemo) | 114 / 114 | 63.9 / 62.6 | 72 / 73 | Both | Stomatitis | NCI CTCAE v3.0 |
| Kim et al., 2016 | NCT01783834 | II | South Korea | IIIB/IV/recurrent NSCLC | ≥2L | Gefitinib 250 mg PO QD | Pemetrexed 500 mg/m² IV q3w (chemo) | 106 / 87 | 59 / 59 | 63 / 50 | Both | Stomatitis | NCI CTCAE v3.0 |
| NEJ009, 2020 (Hosomi et al.) | — | III | Japan | IIIB/IV/relapsed nonsquamous NSCLC | 1L | Gefitinib 250 mg PO QD | Gefitinib 250 mg PO QD + carboplatin AUC 5 IV + pemetrexed 500 mg/m² IV q3w, ≤6 cycles; then gefitinib + pemetrexed maintenance | 172 / 170 | 64 / 64.8 | 108 / 114 | Both | Stomatitis | NCI CTCAE v4.0 |
| ISEL, 2005 (Thatcher et al.) | NCT00242801 | III | Multicountry | Advanced/metastatic NSCLC | ≥2L | Gefitinib 250 mg PO QD | Placebo PO QD | 1129 / 563 | 62 / 61 | 368 / 185 | Both | Stomatitis | NCI CTCAE v2.0 |
| Yu et al., 2014 | NCT01769066 | II | China | IIIB/IV nonsquamous NSCLC | 1L | Gefitinib 250 mg PO d3-16 + pemetrexed 500 mg/m² IV d2 + cisplatin 75 mg/m² or carboplatin AUC 5 IV d1 q3w | Pemetrexed 500 mg/m² IV d2 + cisplatin 75 mg/m² or carboplatin AUC 5 IV d1 q3w (chemo) | 58 / 59 | 55.3 / 54.9 | 25 / 34 | Both | Stomatitis | NCI CTCAE v3.0 |
| IMPRESS, 2015 (Soria et al.) | NCT01544179 | III | Multicountry | Advanced NSCLC | 2L | Gefitinib 250 mg PO QD + cisplatin 75 mg/m² + pemetrexed 500 mg/m² | Placebo PO QD + cisplatin 75 mg/m² + pemetrexed 500 mg/m² (chemo) | 133 / 132 | 60 / 58 | 87 / 84 | Both | Stomatitis | NCI CTCAE v4.0 |
| An et al., 2016 | — | II | China | IIIB/IV nonsquamous NSCLC | 1L | Gefitinib 250 mg PO d2-16 + pemetrexed 500 mg/m² IV d1 q3w | Placebo PO d2-16 + pemetrexed 500 mg/m² IV d1 q3w (chemo) | 45 / 45 | 65.72 / 66.89 | 20 / 20 | Grade ≥3 only | Oral mucositis | NCI CTCAE v4.0 |
| Yang et al., 2014 | NCT01017874 | III | Multicountry (Asian) | Advanced/metastatic nonsquamous NSCLC | 1L | Cisplatin 75 mg/m² IV + pemetrexed 500 mg/m² IV d1 q3w, 6 cycles; then gefitinib 250 mg PO QD maintenance | Gefitinib 250 mg PO QD | 118 / 118 | 58.53 / 59.36 | 88 / 89 | Any-grade only | Stomatitis | NCI CTCAE v3.0 |
| Cheng et al., 2016 | NCT01469000 | II | Multicountry (Asian) | IV/Recurrent NSCLC | 1L | Gefitinib 250 mg PO QD + pemetrexed 500 mg/m² IV q3w | Gefitinib 250 mg PO QD | 126 / 65 | 62 / 62 | 82 / 41 | Both | Stomatitis | NCI CTCAE v4.0 |
| LASER301, 2023 (Cho et al.) | NCT04248829 | III | Multicountry | Advanced/metastatic adenocarcinoma | 1L | Lazertinib 240 mg PO QD | Gefitinib 250 mg PO QD | 196 / 197 | 67 / 64 | 132 / 119 | Any-grade only | Stomatitis | NCI CTCAE v5.0 |
| EXCLAIM-2, 2025 (Jänne et al.) | NCT04129502 | III | Multicountry | Advanced/metastatic nonsquamous NSCLC | 1L | Mobocertinib 160 mg PO QD | Pemetrexed + cisplatin or carboplatin IV q3w, 4 cycles; then pemetrexed maintenance (chemo; doses partly NR) | 179 / 175 | 64 / 62 | 108 / 116 | Both | Stomatitis | NCI CTCAE v5.0 |
| AURA3, 2017 (Mok et al.) | NCT02151981 | III | Multicountry | Advanced/metastatic NSCLC | 2L | Osimertinib 80 mg PO QD | Pemetrexed 500 mg/m² IV + carboplatin AUC 5 or cisplatin 75 mg/m² IV q3w (chemo) | 279 / 140 | 62 / 63 | 172 / 97 | Both | Stomatitis | NCI CTCAE v4.0 |
| COMPEL, 2025 (Peled et al.) | NCT04765059 | III | Multicountry | IIIB-IVB/recurrent NSCLC | 2L | Osimertinib 80 mg PO QD + pemetrexed 500 mg/m² IV + cisplatin 75 mg/m² or carboplatin IV d1 q3w, 4 cycles; then osimertinib + pemetrexed maintenance | Placebo PO QD + the same platinum-pemetrexed regimen, followed by placebo + pemetrexed maintenance (chemo) | 49 / 49 | 63 / 61 | 30 / 39 | Both | Stomatitis | NCI CTCAE v5.0 |
| LOGIK1604/NEJ032A, TAKUMI, 2021 (Tanaka et al.) | jRCTs071180062 | II | Japan | IIIB/IV nonsquamous NSCLC | 2L | Osimertinib 80 mg PO QD + carboplatin AUC 5 IV + pemetrexed 500 mg/m² IV q3w, 4 cycles; then osimertinib + pemetrexed maintenance | Osimertinib 80 mg PO QD | 31 / 31 | 68 / 68 | 15 / 14 | Both | Stomatitis | — |
| FLAURA2, 2023 (Planchard et al.) | NCT04035486 | III | Multicountry | Advanced/metastatic NSCLC | 1L | Osimertinib 80 mg PO QD + pemetrexed 500 mg/m² IV + cisplatin 75 mg/m² or carboplatin IV q3w | Osimertinib 80 mg PO QD | 279 / 278 | 61 / 62 | 173 / 169 | Both | Stomatitis | NCI CTCAE v5.0 |
| Registry study, 2010 | NCT01196078 | II | China | Advanced NSCLC | 1L | Erlotinib 150 mg PO QD | Vinorelbine 60 mg/m² PO d1,8 in cycle 1, then 80 mg/m² PO d1,8 q3w (chemo) | 57 / 56 | 78 / 78 | 10 / 11 | Any-grade only | Stomatitis | — |
| Ninomiya et al., 2023 | — | II | Japan | IIIB-IV/recurrent non-squamous NSCLC | 1L | Afatinib 30 mg PO QD + bevacizumab 15 mg/kg IV d1 q3w | Afatinib 40 mg PO QD | 49 / 50 | 69 / 71 | 27 / 28 | Both | Stomatitis | NCI CTCAE v4.0 |
| SWOG S1403, 2020 (Goldberg et al.) | NCT02438722 | II/III | Multicountry | IV or Recurrent NSCLC | 1L | Afatinib 40 mg PO QD + cetuximab 500 mg/m² IV q2w | Afatinib PO QD (dose NR) | 83 / 85 | 65.5 / 66.3 | 59 / 53 | Both | Oral mucositis | NCI CTCAE v4.0 |
| IFCT-1503 ACE-Lung, 2021 (Cortot et al.) | NCT02716311 | II | France | IIIB/IV nonsquamous NSCLC | 1L | Afatinib 40 mg PO QD + cetuximab 500 mg/m² IV q2w for 6 months (first dose 250 mg/m² on d15) | Afatinib 40 mg PO QD | 58 / 59 | 63.8 / 68.1 | 41 / 43 | Both | Stomatitis | NCI CTCAE v4.0 |
| FL-ALTER, 2024 (Zhou et al.) | NCT04028778 | III | China | IIIB/IV NSCLC | 1L | Gefitinib 250 mg PO QD + anlotinib 12 mg PO d1-14 q3w | Gefitinib 250 mg PO QD + placebo d1-14 q3w | 155 / 155 | 59 / 59 | 96 / 96 | Both | Oral mucositis | NCI CTCAE v4.0 |
| ECOG-ACRIN 1512, 2017 (Neal et al.) | NCT01708954 | II | US | Metastatic or recurrent nonsquamous NSCLC | ≥2L | Cabozantinib 60 mg PO QD; cabozantinib60 mg + erlotinib150 mg PO QD | Erlotinib 150 mg PO QD (three-arm trial) | 38 / 35 / NR† | 66.3 / 63.5 | 20 / 17 | Both | Oral mucositis | NCI CTCAE v4.0 |
| BeTa, 2011 (Herbst et al.) | NCT00130728 | III | Multicountry | Advanced or recurrent NSCLC | 2L | Erlotinib 150 mg PO QD + bevacizumab 15 mg/kg IV d1 q3w | Erlotinib 150 mg PO QD + placebo IV d1 q3w | 319 / 317 | 64.8 / 65 | 148 / 170 | Any-grade only | Stomatitis | NCI CTCAE v3.0 |
| NEJ026, 2019 (Saito et al.) | jRCTs031180056 | III | Japan | IIIB-IV nonsquamous NSCLC | 1L | Erlotinib 150 mg PO QD + bevacizumab 15 mg/kg IV q3w | Erlotinib 150 mg PO QD | 112 / 112 | 67 / 68 | 71 / 73 | Both | Stomatitis | NCI CTCAE v4.0 |
| Lee et al., 2023 | NCT03126799 | II | South Korea | IIIB/IV NSCLC | 1L | Erlotinib 150 mg PO QD + bevacizumab 15 mg/kg IV d1 q3w | Erlotinib 150 mg PO QD | 64 / 63 | NR | 44 / 40 | Both | Oral mucositis | NCI CTCAE v4.0 |
| BEVERLY, 2022 (Piccirillo et al.) | NCT02633189 | III | Italy | Advanced/metastatic nonsquamous NSCLC | 1L | Erlotinib 150 mg PO QD + bevacizumab 15 mg/kg IV q3w | Erlotinib 150 mg PO QD | 80 / 80 | 65.9 / 67.7 | 52 / 50 | Both | Oral mucositis | NCI CTCAE v4.03 |
| Stinchcomb et al., 2019 | NCT01532089 | II | US | IV nonsquamous NSCLC | 1L | Erlotinib 150 mg PO QD + bevacizumab 15 mg/kg IV d1 q3w | Erlotinib 150 mg PO QD | 43 / 45 | 65 / 63 | 31 / 31 | Any-grade only | Oral mucositis | NCI CTCAE v4.0 |
| BOOSTER, 2022 (Soo et al.) | NCT03133546 | II | Multicountry | IIIb-IVb nonsquamous NSCLC | 2L | Osimertinib 80 mg PO QD + bevacizumab 15 mg/kg IV d1 q3w | Osimertinib 80 mg PO QD | 78 / 77 | 68 / 66 | 47 / 49 | Both | Oral mucositis | NCI CTCAE v4.0 |
| WJOG9717L, 2022 (Kenmots et al.) | — | II | Japan | IIIB-IIIC/IV/postoperative recurrence nonsquamous NSCLC | 1L | Osimertinib 80 mg PO QD + bevacizumab 15 mg/kg IV d1 q3w | Osimertinib 80 mg PO QD | 61 / 61 | 67 / 66 | 37 / 38 | Both | Oral mucositis | NCI CTCAE v4.0 |
| RELAY, 2019 (Nakagawa et al.) | NCT02411448 | III | Multicountry | IV NSCLC | 1L | Erlotinib 150 mg PO QD + ramucirumab 10 mg/kg IV q2w | Erlotinib 150 mg PO QD + placebo IV q2w | 224 / 225 | 65 / 64 | 141 / 142 | Both | Stomatitis | NCI CTCAE v4.0 |
| Groen et al., 2013 | NCT00265317 | II | Multicountry | IIIB/IV NSCLC | ≥2L | Erlotinib 150 mg PO QD + sunitinib 37.5 mg PO QD (4-week cycle) | Erlotinib 150 mg PO QD + placebo PO QD (4-week cycle) | 65 / 67 | 59 / 61 | 25 / 22 | Any-grade only | Stomatitis | NCI CTCAE v3.0 |
| SUN1087, 2012 (Scagliotti et al.) | NCT00457392 | III | Multicountry | IIIB/IV NSCLC | ≥2L | Erlotinib 150 mg PO QD + sunitinib 37.5 mg PO QD (4-week cycle) | Erlotinib 150 mg PO QD + placebo PO QD (4-week cycle) | 480 / 480 | 61 / 61 | 173 / 196 | Any-grade only | Stomatitis | NCI CTCAE v3.0 |
| KCSG LU12-01, 2017 (Kim et al.) | NCT01498562 | II | South Korea | IV NSCLC | ≥2L | Gefitinib 250 mg PO QD + nimotuzumab 200 mg IV weekly | Gefitinib 250 mg PO QD | 80 / 80 | 63 / 63 | 26 / 32 | Both | Stomatitis | NCI CTCAE v4.0 |

**Supplementary Table S3.** SUCRA-based risk rankings for any-grade and grade ≥3 oral mucositis.

**Panel A. Any-grade OM**

| **Rank** | **Treatment** | **SUCRA (%)** |
| --- | --- | --- |
| 1 | Afatinib + cetuximab | 89.0 |
| 2 | Afatinib | 88.4 |
| 3 | Erlotinib + cabozantinib | 81.2 |
| 4 | Dacomitinib | 75.2 |
| 5 | Afatinib + bevacizumab | 71.7 |
| 6 | Gefitinib + anlotinib | 67.9 |
| 7 | Mobocertinib | 66.8 |
| 8 | Afatinib + chemotherapy | 63.4 |
| 9 | Erlotinib + chemotherapy | 59.6 |
| 10 | Lazertinib | 54.8 |
| 11 | Erlotinib + bevacizumab | 54.5 |
| 12 | Gefitinib + chemotherapy | 51.2 |
| 13 | Osimertinib + bevacizumab | 46.5 |
| 14 | Erlotinib + sunitinib | 45.6 |
| 15 | Erlotinib + ramucirumab | 43.7 |
| 16 | Osimertinib + chemotherapy | 39.5 |
| 17 | Aumolertinib | 35.6 |
| 18 | Erlotinib | 35.1 |
| 19 | Gefitinib | 30.1 |
| 20 | Gefitinib + nimotuzumab | 29.5 |
| 21 | Osimertinib | 23.2 |
| 22 | Chemotherapy | 22.9 |
| 23 | Furmonertinib | 21.0 |
| 24 | Placebo | 3.5 |

**Panel B. Grade ≥3 OM**

| **Rank** | **Treatment** | **SUCRA (%)** |
| --- | --- | --- |
| 1 | Afatinib + cetuximab | 91.6 |
| 2 | Afatinib + bevacizumab | 88.4 |
| 3 | Mobocertinib | 86.4 |
| 4 | Afatinib | 86.3 |
| 5 | Afatinib + chemotherapy | 84.0 |
| 6 | Dacomitinib | 68.7 |
| 7 | Chemotherapy | 61.9 |
| 8 | Gefitinib + chemotherapy | 60.6 |
| 9 | Gefitinib + anlotinib | 55.3 |
| 10 | Erlotinib + chemotherapy | 49.8 |
| 11 | Erlotinib + cabozantinib | 42.8 |
| 12 | Erlotinib + ramucirumab | 36.5 |
| 13 | Erlotinib + bevacizumab | 34.8 |
| 14 | Erlotinib | 32.8 |
| 15 | Furmonertinib | 30.0 |
| 16 | Gefitinib | 29.8 |
| 17 | Osimertinib + bevacizumab | 16.9 |
| 18 | Osimertinib + chemotherapy | 16.6 |
| 19 | Osimertinib | 16.6 |
| 20 | Placebo | 10.2 |

**Supplementary Table S4.** Model fit and between-study heterogeneity for any-grade and grade ≥3 oral mucositis.

| **Outcome** | **Model** | **Dbar** | **pD** | **DIC** | **Data points** | **Residual deviance ratio** | **τ, median (95% CrI)** | **ΔDIC vs random effects** |
| --- | --- | --- | --- | --- | --- | --- | --- | --- |
| Any-grade OM | Random effects | 126.907 | 112.985 | 239.892 | 123 | 1.032 | 0.890 (0.648–1.238) | 0.000 |
| Any-grade OM | Fixed effect | 301.398 | 84.854 | 386.252 | 123 | 2.450 | — | 146.360 |
| Grade ≥3 OM | Random effects | 92.589 | 47.099 | 139.688 | 85 | 1.089 | 0.361 (0.020–0.952) | 0.000 |
| Grade ≥3 OM | Fixed effect | 96.008 | 45.251 | 141.259 | 85 | 1.130 | — | 1.571 |

**Supplementary Table S5.** Global model comparison and summary of local inconsistency assessments.

| **Outcome** | **Model** | **Dbar** | **pD** | **DIC** | **Data points** | **Residual deviance ratio** | **ΔDIC vs consistency** |
| --- | --- | --- | --- | --- | --- | --- | --- |
| Any-grade OM | Consistency, random effects | 126.907 | 112.985 | 239.892 | 123 | 1.032 | 0.000 |
| Any-grade OM | UME, random effects | 127.160 | 115.304 | 242.464 | 123 | 1.034 | 2.572 |
| Grade ≥3 OM | Consistency, random effects | 92.589 | 47.099 | 139.688 | 85 | 1.089 | 0.000 |
| Grade ≥3 OM | UME, random effects | 95.018 | 49.292 | 144.309 | 85 | 1.118 | 4.621 |

**Supplementary Table S6.** Node-splitting summary

| **Outcome** | **Evaluable comparisons** | **Minimum P value** | **Interpretation** |
| --- | --- | --- | --- |
| Any-grade OM | 17 | 0.105 | No comparison showed evidence of important local inconsistency |
| Grade ≥3 OM | 15 | 0.137 | No comparison showed evidence of important local inconsistency |

**Supplementary Table S7.** Convergence diagnostics for the primary Bayesian network meta-analysis models.

| **Outcome** | **MCMC sampling** | **Maximum R-hat point estimate** | **Maximum R-hat upper limit** | **Effective sample size range** | **Interpretation** |
| --- | --- | --- | --- | --- | --- |
| Any-grade OM | 4 chains; 20,000 adaptation iterations; 50,000 sampling iterations; thin=1 | 1.000450 | 1.001268 | Not exported in supplied files | Satisfactory |
| Grade ≥3 OM | 4 chains; 20,000 retained samples per chain; thin=10 | 1.001346 | 1.003888 | 4,114–22,574 | Satisfactory |

**Supplementary Table S8.** Outcome-specific sensitivity analyses using alternative prior distributions and model specifications.

**Panel A. Any-grade OM**

| **Analysis** | **Prior/model specification** | **DIC** | **τ, median (95% CrI)** | **Same OR direction vs primary*** | **Same 95% CrI conclusion*** | **SUCRA stability** |
| --- | --- | --- | --- | --- | --- | --- |
| Primary random-effects model | τ ~ Uniform(0, 3.981) | 239.892 | 0.890 (0.648–1.238) | Reference | Reference | Reference |
| Alternative random-effects model | 1/τ² ~ Gamma(0.01,0.01) | 240.136 | 0.868 (0.633–1.200) | 23/23 | 23/23 | ρ=1.000; max Δ=0.45 percentage points |
| Alternative random-effects model | τ ~ Half-Normal(0,1) | 239.688 | 0.873 (0.640–1.199) | 23/23 | 23/23 | ρ=1.000; max Δ=0.34 percentage points |
| Fixed-effect model | No between-study heterogeneity | 386.252 | — | Not used as prior-sensitivity evidence | Not used as prior-sensitivity evidence | Model fit was markedly worse |

**Counts refer to all 23 chemotherapy-referenced treatment comparisons. Under both alternative random-effects priors, all 23 comparisons retained the same effect direction and the same conclusion regarding whether the 95% CrI included 1. SUCRA rankings were unchanged (Spearman ρ=1.000).*

**Panel B. Grade ≥3 OM**

| **Analysis** | **Prior/model specification** | **DIC** | **Residual deviance ratio** | **Same OR direction vs primary*** | **Same 95% CrI conclusion*** | **Comparisons with changed CrI conclusion** |
| --- | --- | --- | --- | --- | --- | --- |
| Primary random-effects model | Effect SD 2.82; τ ~ Half-Normal(0,0.5) | 139.688 | 1.089 | Reference | Reference | — |
| Alternative heterogeneity prior | Effect SD 2.82; τ ~ Half-Normal(0,1) | 139.458 | 1.076 | 19/19 | 18/19 | Mobocertinib |
| More regularizing effect prior | Effect SD 2.0; τ ~ Half-Normal(0,0.5) | 146.253 | 1.238 | 19/19 | 17/19 | Erlotinib_Bevacizumab; Mobocertinib |
| Fixed-effect model | Effect SD 2.82; no τ | 141.259 | 1.130 | 19/19 | 18/19 | Erlotinib_Bevacizumab |

**Counts refer to 19 chemotherapy-referenced comparisons. All alternative analyses preserved the effect direction for all 19 comparisons. The small number of changes in whether the 95% CrI included 1 occurred mainly for mobocertinib and erlotinib plus bevacizumab.*

**Supplementary Table S9.** Network characteristics, model fit, and between-study heterogeneity for subgroup analyses of any-grade oral mucositis.

| **Subgroup factor** | **Subgroup** | **RCTs** | **Participants** | **Treatment nodes** | **DIC** | **Residual deviance ratio** | **τ, median (95% CrI)** |
| --- | --- | --- | --- | --- | --- | --- | --- |
| Treatment line | First-line | 32 | 8,668 | 16 | 115.334 | 1.016 | 0.155 (0.011–0.518) |
| Treatment line | ≥2-line | 26 | 11,361 | 16 | 105.255 | 1.011 | 1.415 (0.880–2.492) |
| Population composition | Exclusively Asian | 15 | 3,559 | 9 | 60.116 | 1.103 | 0.430 (0.019–1.624) |
| Population composition | Non-exclusively Asian | 37 | 14,901 | 19 | 146.960 | 1.015 | 1.117 (0.739–1.759) |

**Supplementary Table S10.** SUCRA-based risk rankings in subgroup analyses of any-grade oral mucositis.

**Panel A. First-line subgroup**

| **Rank** | **Treatment** | **SUCRA (%)** | **Mean rank** | **95% rank interval** |
| --- | --- | --- | --- | --- |
| 1 | Afatinib + cetuximab | 93.7 | 1.95 | 1–6 |
| 2 | Afatinib | 89.1 | 2.63 | 1–6 |
| 3 | Erlotinib + bevacizumab | 75.6 | 4.65 | 1–10 |
| 4 | Dacomitinib | 73.5 | 4.98 | 1–10 |
| 5 | Gefitinib + anlotinib | 73.2 | 5.01 | 1–12 |
| 6 | Afatinib + bevacizumab | 64.0 | 6.40 | 1–13 |
| 7 | Erlotinib + ramucirumab | 57.0 | 7.46 | 2–13 |
| 8 | Lazertinib | 50.2 | 8.47 | 3–13 |
| 9 | Mobocertinib | 49.6 | 8.55 | 3–13 |
| 10 | Erlotinib | 45.6 | 9.15 | 4–13 |
| 11 | Erlotinib + chemotherapy | 44.2 | 9.37 | 5–13 |
| 12 | Gefitinib + chemotherapy | 36.4 | 10.53 | 6–13 |
| 13 | Aumolertinib | 21.1 | 12.83 | 7–16 |
| 14 | Gefitinib | 17.0 | 13.45 | 12–15 |
| 15 | Furmonertinib | 6.4 | 15.03 | 12–16 |
| 16 | Chemotherapy | 3.2 | 15.52 | 14–16 |

**Panel B. ≥2-line subgroup**

| **Rank** | **Treatment** | **SUCRA (%)** | **Mean rank** | **95% rank interval** |
| --- | --- | --- | --- | --- |
| 1 | Afatinib | 73.1 | 5.04 | 1–13 |
| 2 | Afatinib + chemotherapy | 71.2 | 5.32 | 1–15 |
| 3 | Erlotinib + chemotherapy | 70.5 | 5.42 | 1–13 |
| 4 | Gefitinib + chemotherapy | 68.9 | 5.67 | 1–15 |
| 5 | Erlotinib + cabozantinib | 68.5 | 5.73 | 1–15 |
| 6 | Dacomitinib | 62.8 | 6.57 | 1–14 |
| 7 | Osimertinib + bevacizumab | 59.8 | 7.03 | 1–16 |
| 8 | Osimertinib + chemotherapy | 57.4 | 7.38 | 1–15 |
| 9 | Chemotherapy | 47.0 | 8.95 | 5–13 |
| 10 | Osimertinib | 41.8 | 9.74 | 3–16 |
| 11 | Erlotinib + bevacizumab | 41.5 | 9.78 | 1–16 |
| 12 | Erlotinib + sunitinib | 37.6 | 10.37 | 3–16 |
| 13 | Gefitinib + nimotuzumab | 33.1 | 11.04 | 2–16 |
| 14 | Gefitinib | 32.3 | 11.15 | 5–15 |
| 15 | Erlotinib | 27.7 | 11.84 | 7–15 |
| 16 | Placebo | 7.0 | 14.96 | 10–16 |

**Panel C. Exclusively Asian subgroup**

| **Rank** | **Treatment** | **SUCRA (%)** | **Mean rank** | **95% rank interval** |
| --- | --- | --- | --- | --- |
| 1 | Erlotinib + bevacizumab | 88.6 | 1.91 | 1–6 |
| 2 | Gefitinib + anlotinib | 82.3 | 2.42 | 1–7 |
| 3 | Gefitinib + chemotherapy | 68.4 | 3.53 | 1–7 |
| 4 | Erlotinib | 64.8 | 3.82 | 2–8 |
| 5 | Aumolertinib | 40.1 | 5.79 | 2–9 |
| 6 | Gefitinib | 37.9 | 5.97 | 4–8 |
| 7 | Gefitinib + nimotuzumab | 29.4 | 6.65 | 2–9 |
| 8 | Chemotherapy | 22.5 | 7.20 | 4–9 |
| 9 | Furmonertinib | 16.1 | 7.71 | 3–9 |

**Panel D. Non-exclusively Asian subgroup**

| **Rank** | **Treatment** | **SUCRA (%)** | **Mean rank** | **95% rank interval** |
| --- | --- | --- | --- | --- |
| 1 | Afatinib + cetuximab | 78.0 | 4.96 | 1–15 |
| 2 | Afatinib | 76.9 | 5.15 | 1–12 |
| 3 | Erlotinib + cabozantinib | 76.0 | 5.32 | 1–17 |
| 4 | Mobocertinib | 71.6 | 6.12 | 1–17 |
| 5 | Afatinib + chemotherapy | 68.6 | 6.65 | 1–18 |
| 6 | Gefitinib + chemotherapy | 65.6 | 7.20 | 1–18 |
| 7 | Dacomitinib | 63.1 | 7.64 | 2–15 |
| 8 | Erlotinib + chemotherapy | 58.8 | 8.41 | 3–15 |
| 9 | Osimertinib + bevacizumab | 57.7 | 8.62 | 1–19 |
| 10 | Osimertinib + chemotherapy | 45.9 | 10.73 | 2–18 |
| 11 | Erlotinib + bevacizumab | 43.6 | 11.16 | 3–18 |
| 12 | Erlotinib + sunitinib | 41.4 | 11.54 | 3–18 |
| 13 | Lazertinib | 40.6 | 11.69 | 2–19 |
| 14 | Erlotinib + ramucirumab | 40.0 | 11.81 | 2–19 |
| 15 | Osimertinib | 35.8 | 12.56 | 4–18 |
| 16 | Chemotherapy | 35.5 | 12.61 | 8–17 |
| 17 | Erlotinib | 30.4 | 13.54 | 9–17 |
| 18 | Gefitinib | 18.9 | 15.61 | 9–18 |
| 19 | Placebo | 1.7 | 18.69 | 16–19 |
